# Supplementary figures and images for: mTOR controls ependymal cell differentiation by targeting the alternative cell cycle and centrosomal proteins (part 1 of 2)
Source: EMBO Rep. 2025 Apr 30;26(12):3075–105. doi: 10.1038/s44319-025-00460-2 (PMC12187940; doi:10.1038/s44319-025-00460-2)

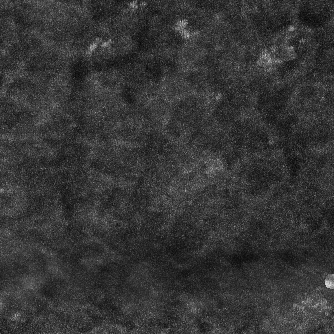

Supplement: Supplementary file 6 — Source data Fig. 1 [file 44319_2025_460_MOESM6_ESM.zip › Figure1/1A/1A_highMag.tif]

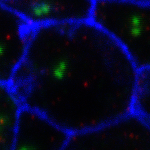

Supplement: Supplementary file 6 — Source data Fig. 1 [file 44319_2025_460_MOESM6_ESM.zip › Figure1/1C/1C1.tif]

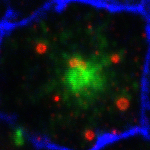

Supplement: Supplementary file 6 — Source data Fig. 1 [file 44319_2025_460_MOESM6_ESM.zip › Figure1/1C/1C2.tif]

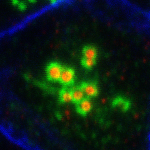

Supplement: Supplementary file 6 — Source data Fig. 1 [file 44319_2025_460_MOESM6_ESM.zip › Figure1/1C/1C3.tif]

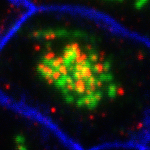

Supplement: Supplementary file 6 — Source data Fig. 1 [file 44319_2025_460_MOESM6_ESM.zip › Figure1/1C/1C4.tif]

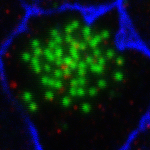

Supplement: Supplementary file 6 — Source data Fig. 1 [file 44319_2025_460_MOESM6_ESM.zip › Figure1/1C/1C5.tif]

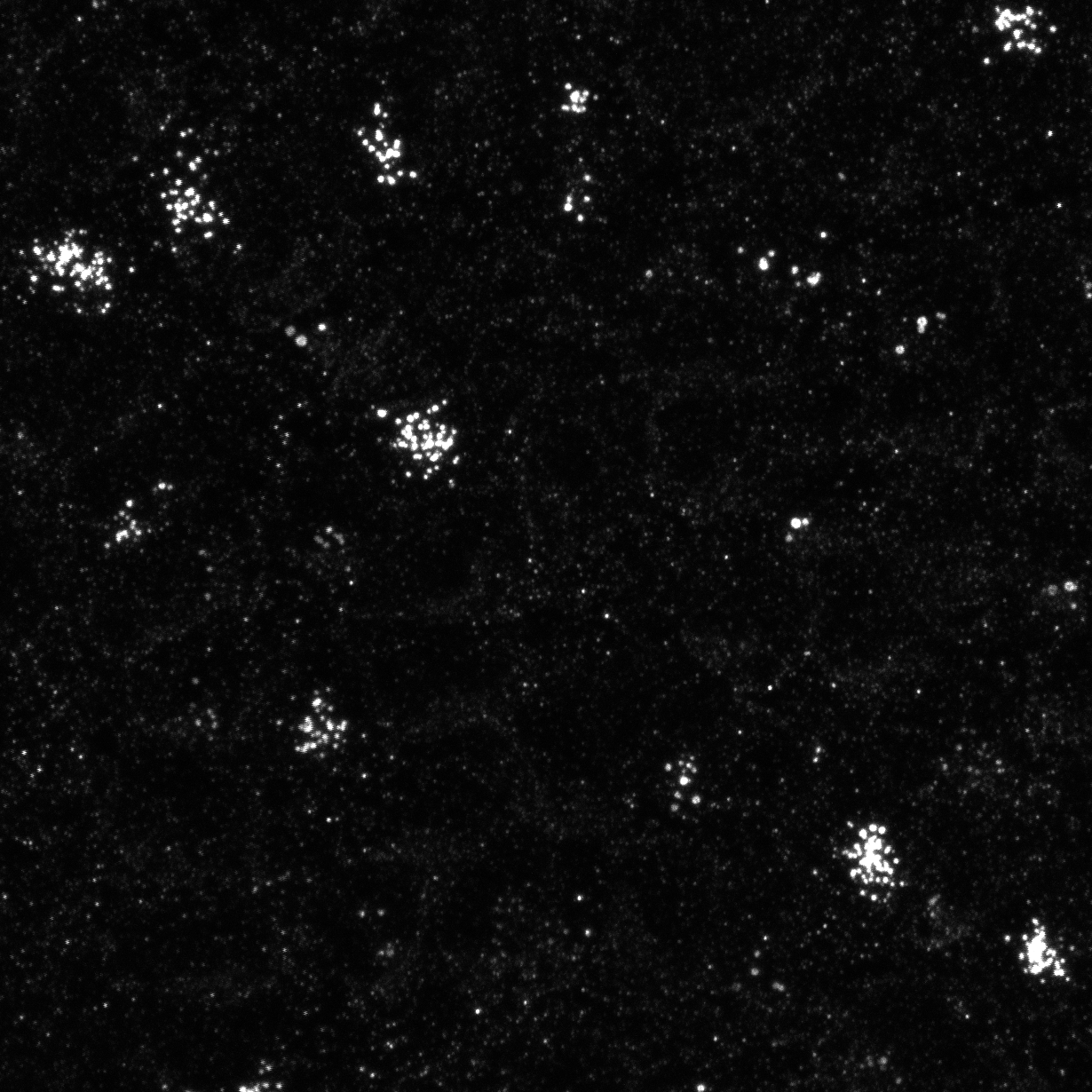

Supplement: Supplementary file 7 — Source data Fig. 2 [file 44319_2025_460_MOESM7_ESM.zip › Figure 2/2A/2A1.tif]

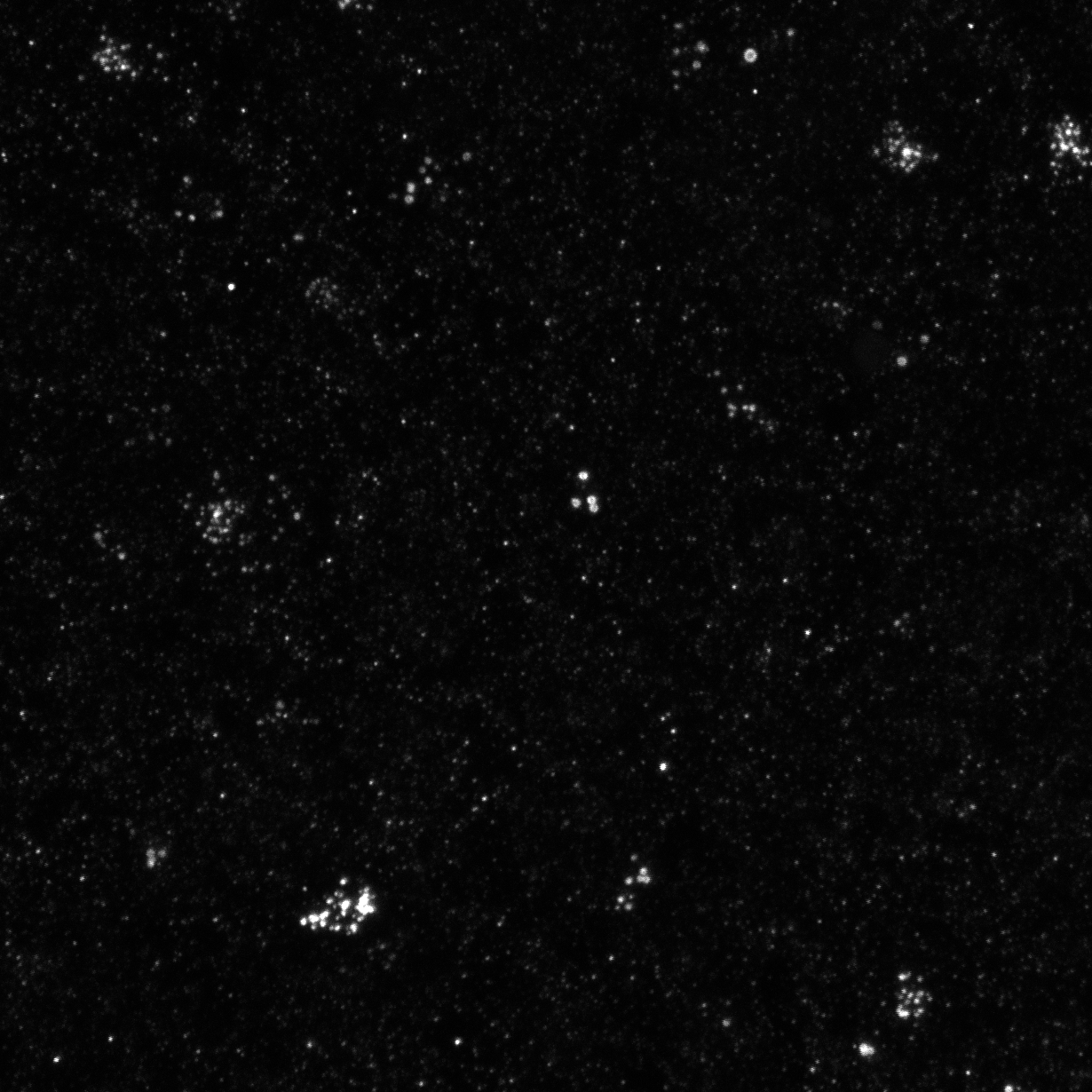

Supplement: Supplementary file 7 — Source data Fig. 2 [file 44319_2025_460_MOESM7_ESM.zip › Figure 2/2A/2A2.tif]

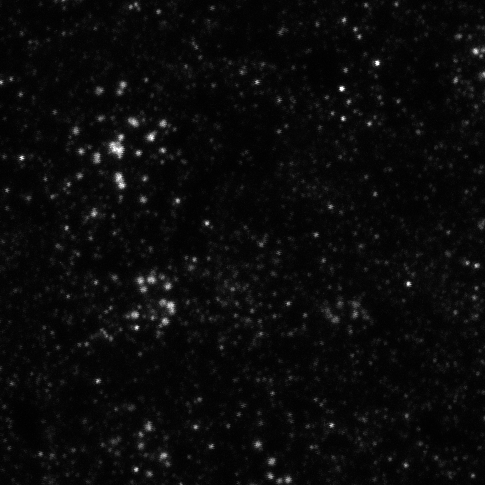

Supplement: Supplementary file 7 — Source data Fig. 2 [file 44319_2025_460_MOESM7_ESM.zip › Figure 2/2D/2D1.tif]

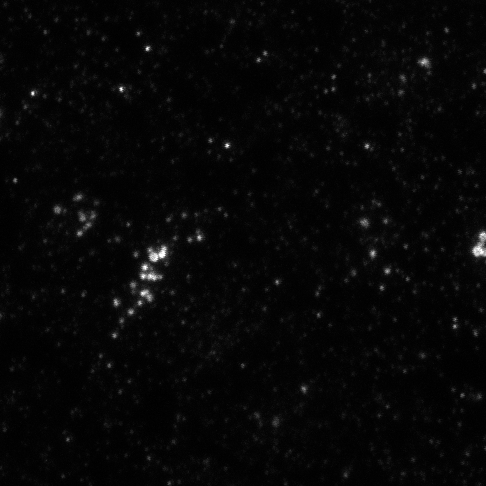

Supplement: Supplementary file 7 — Source data Fig. 2 [file 44319_2025_460_MOESM7_ESM.zip › Figure 2/2D/2D2.tif]

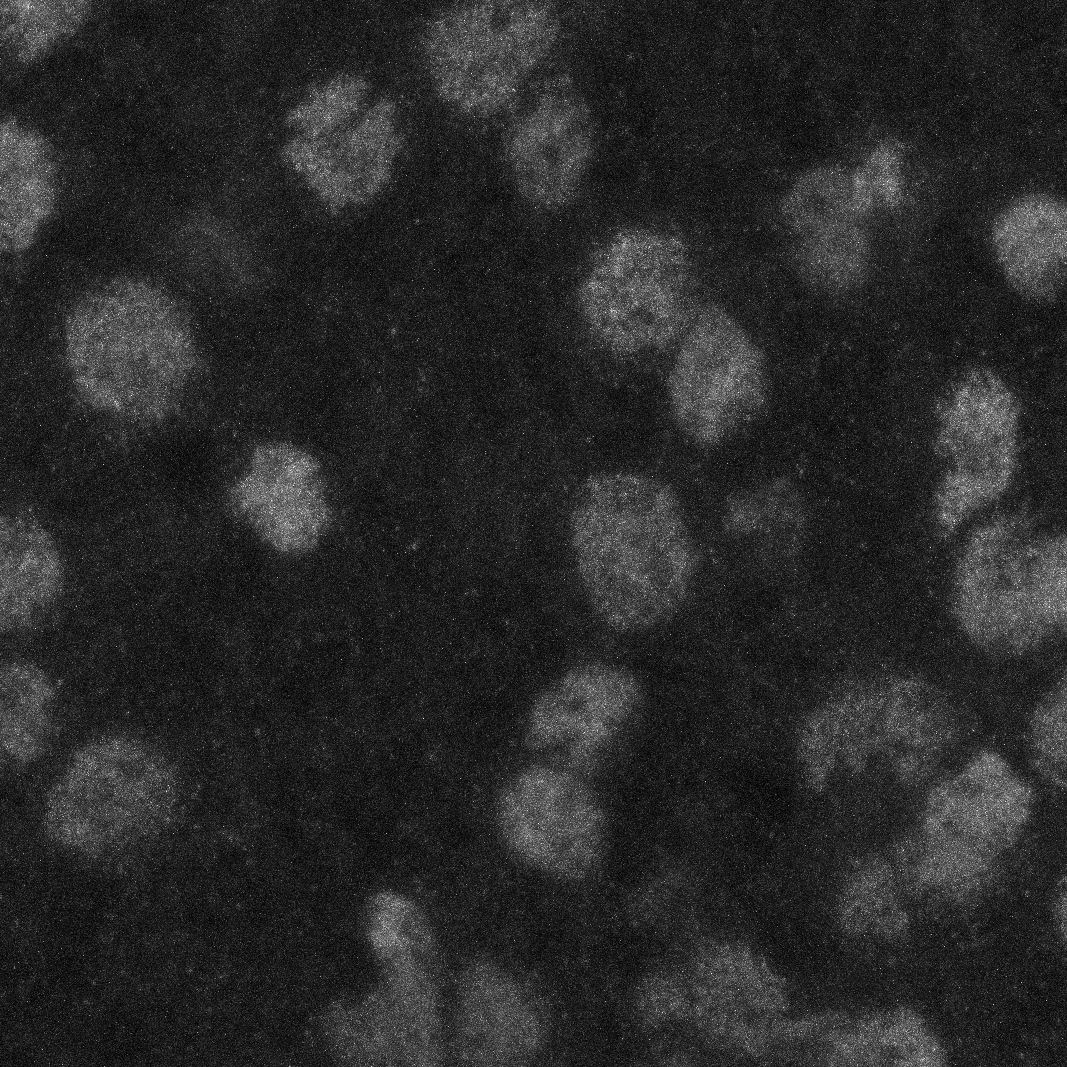

Supplement: Supplementary file 7 — Source data Fig. 2 [file 44319_2025_460_MOESM7_ESM.zip › Figure 2/2G/2G1.tif]

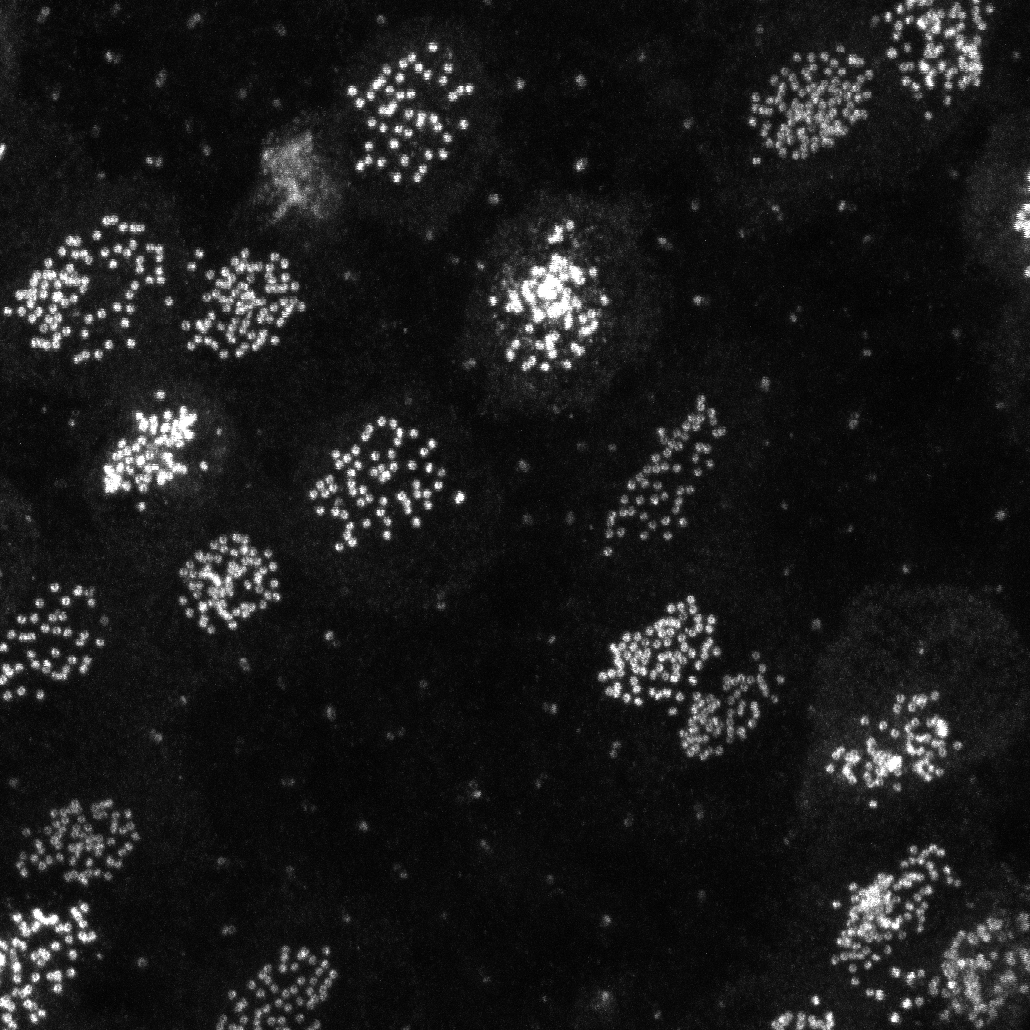

Supplement: Supplementary file 7 — Source data Fig. 2 [file 44319_2025_460_MOESM7_ESM.zip › Figure 2/2G/2G2.tif]

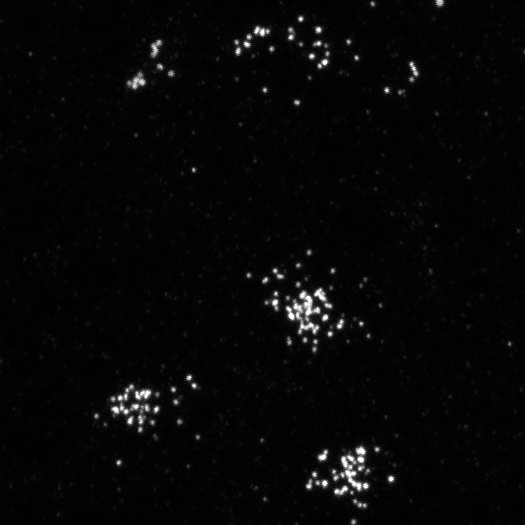

Supplement: Supplementary file 7 — Source data Fig. 2 [file 44319_2025_460_MOESM7_ESM.zip › Figure 2/2I/2I1.tif]

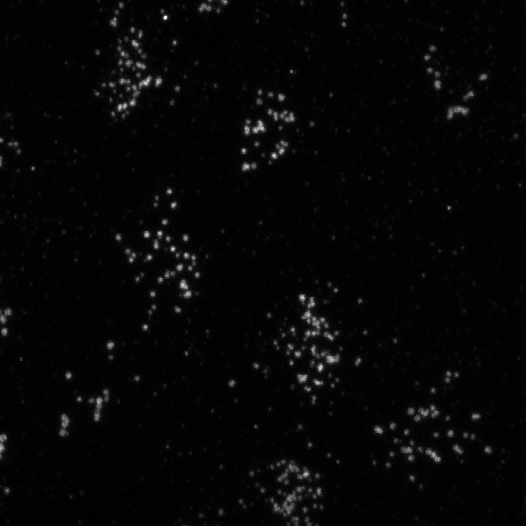

Supplement: Supplementary file 7 — Source data Fig. 2 [file 44319_2025_460_MOESM7_ESM.zip › Figure 2/2I/2I2.tif]

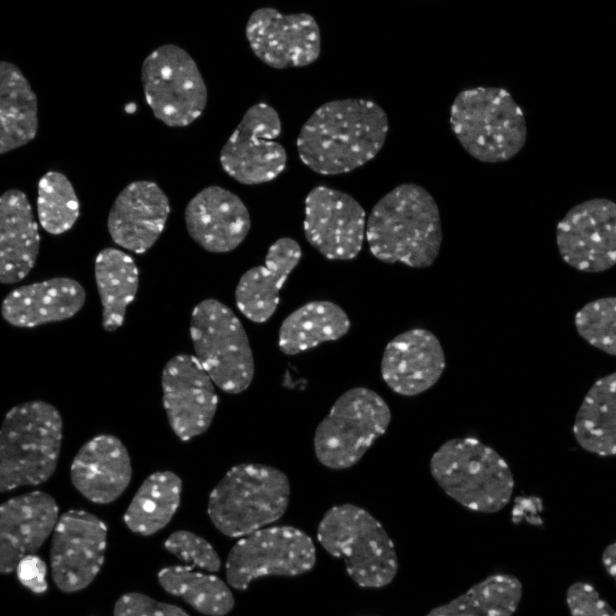

Supplement: Supplementary file 8 — Source data Fig. 3 [file 44319_2025_460_MOESM8_ESM.zip › Figure 3/3A/3A1.tif]

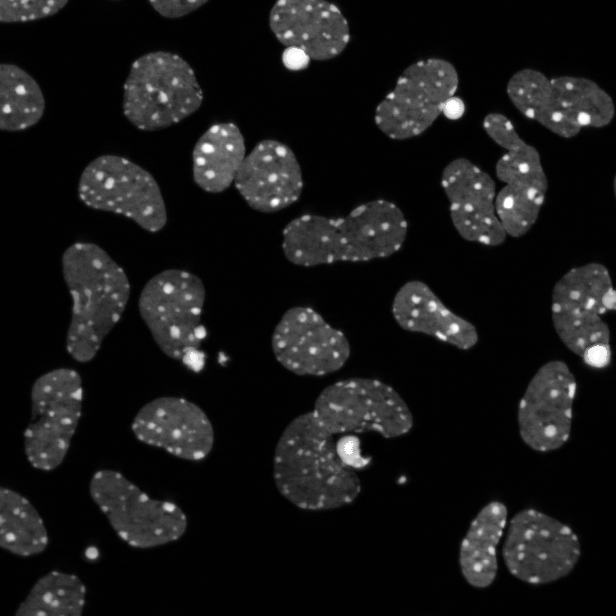

Supplement: Supplementary file 8 — Source data Fig. 3 [file 44319_2025_460_MOESM8_ESM.zip › Figure 3/3A/3A2.tif]

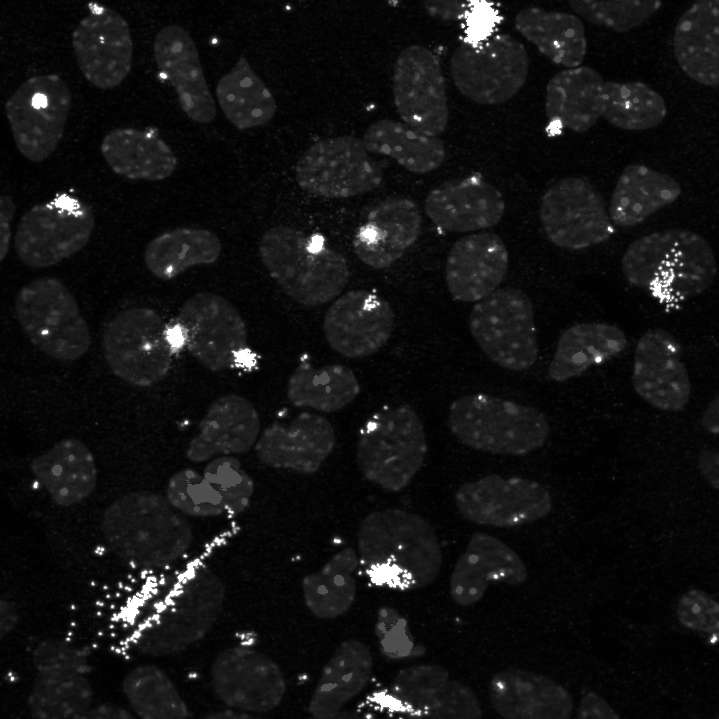

Supplement: Supplementary file 8 — Source data Fig. 3 [file 44319_2025_460_MOESM8_ESM.zip › Figure 3/3C/3C1.tif]

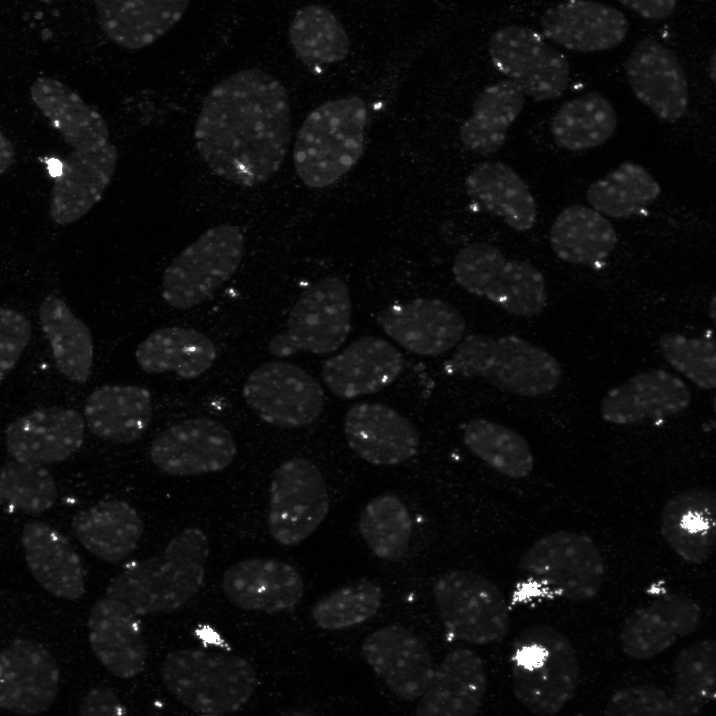

Supplement: Supplementary file 8 — Source data Fig. 3 [file 44319_2025_460_MOESM8_ESM.zip › Figure 3/3C/3C2.tif]

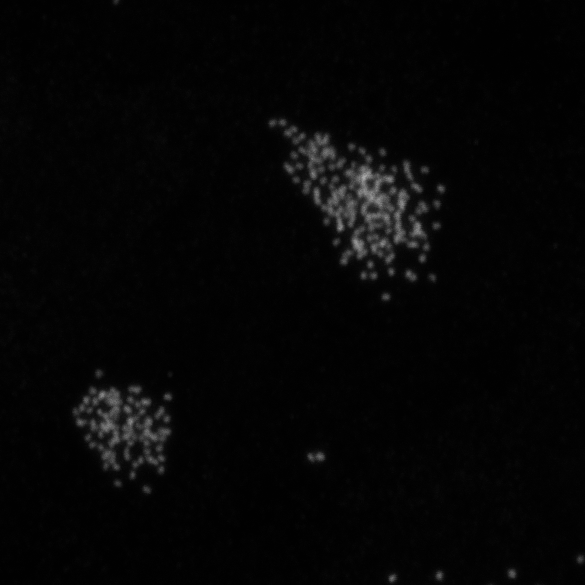

Supplement: Supplementary file 8 — Source data Fig. 3 [file 44319_2025_460_MOESM8_ESM.zip › Figure 3/3E/3E1.tif]

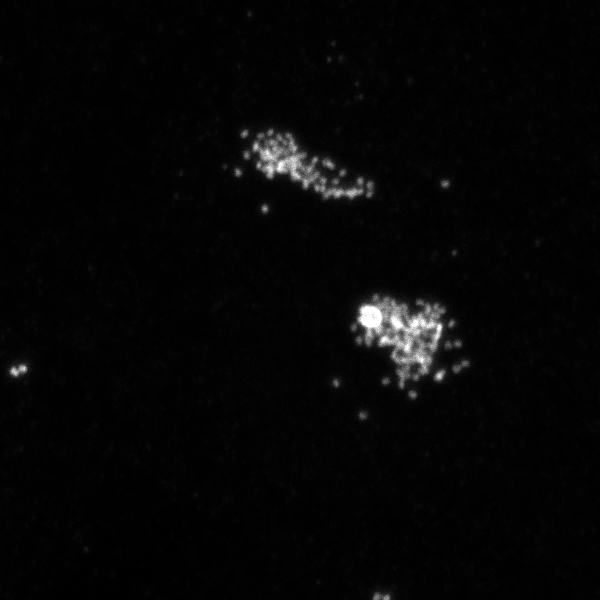

Supplement: Supplementary file 8 — Source data Fig. 3 [file 44319_2025_460_MOESM8_ESM.zip › Figure 3/3E/3E2.tif]

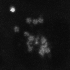

Supplement: Supplementary file 8 — Source data Fig. 3 [file 44319_2025_460_MOESM8_ESM.zip › Figure 3/3H/Deuterosome Regrouping - 1.tif]

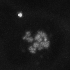

Supplement: Supplementary file 8 — Source data Fig. 3 [file 44319_2025_460_MOESM8_ESM.zip › Figure 3/3H/Deuterosome Regrouping - 2.tif]

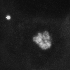

Supplement: Supplementary file 8 — Source data Fig. 3 [file 44319_2025_460_MOESM8_ESM.zip › Figure 3/3H/Deuterosome Regrouping - 3.tif]

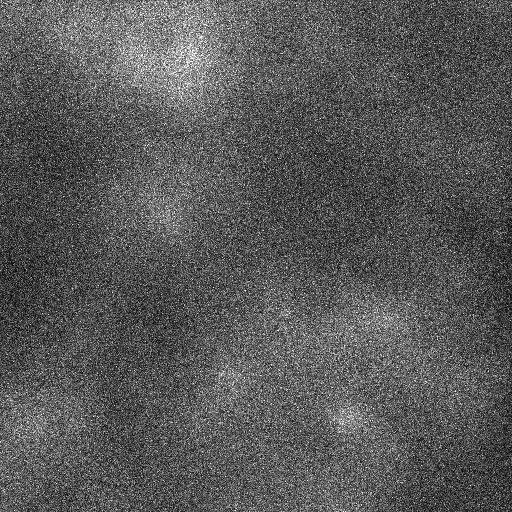

Supplement: Supplementary file 8 — Source data Fig. 3 [file 44319_2025_460_MOESM8_ESM.zip › Figure 3/3H/Normal disengagement - 3H1.TIF]

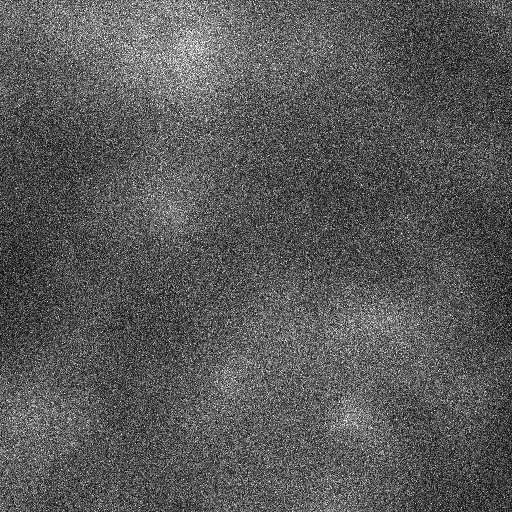

Supplement: Supplementary file 8 — Source data Fig. 3 [file 44319_2025_460_MOESM8_ESM.zip › Figure 3/3H/Normal disengagement - 3H2.TIF]

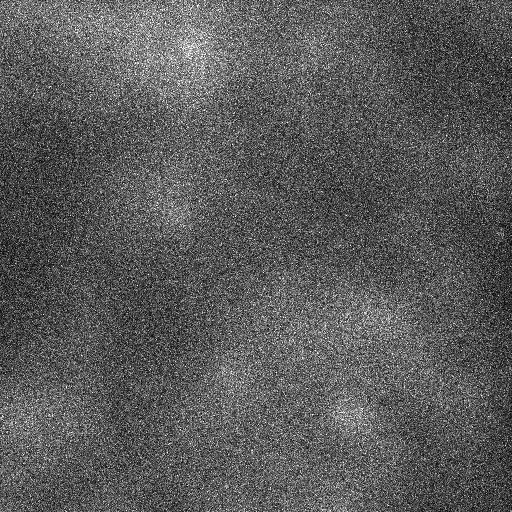

Supplement: Supplementary file 8 — Source data Fig. 3 [file 44319_2025_460_MOESM8_ESM.zip › Figure 3/3H/Normal disengagement - 3H3.TIF]

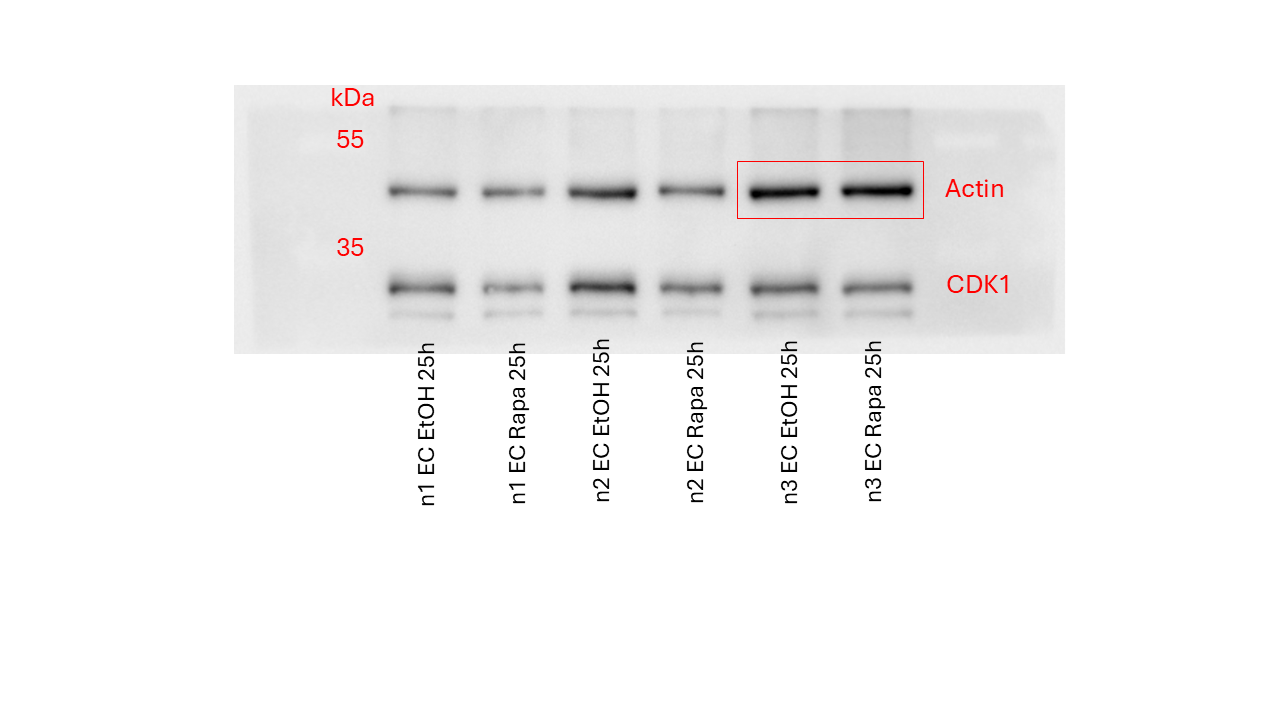

Supplement: Supplementary file 9 — Source data Fig. 5 [file 44319_2025_460_MOESM9_ESM.zip › Figure 5/A/Actin for CDK1 and pWee1.tif]

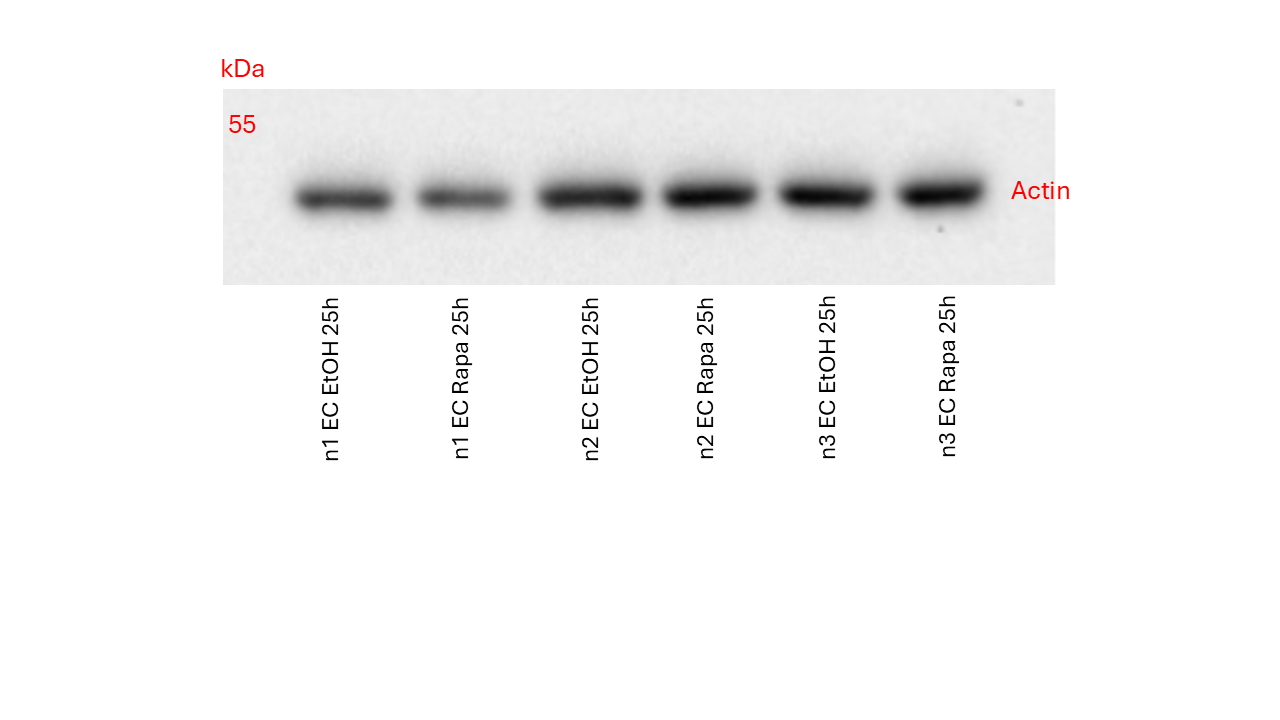

Supplement: Supplementary file 9 — Source data Fig. 5 [file 44319_2025_460_MOESM9_ESM.zip › Figure 5/A/Actin for pCDK1.tif]

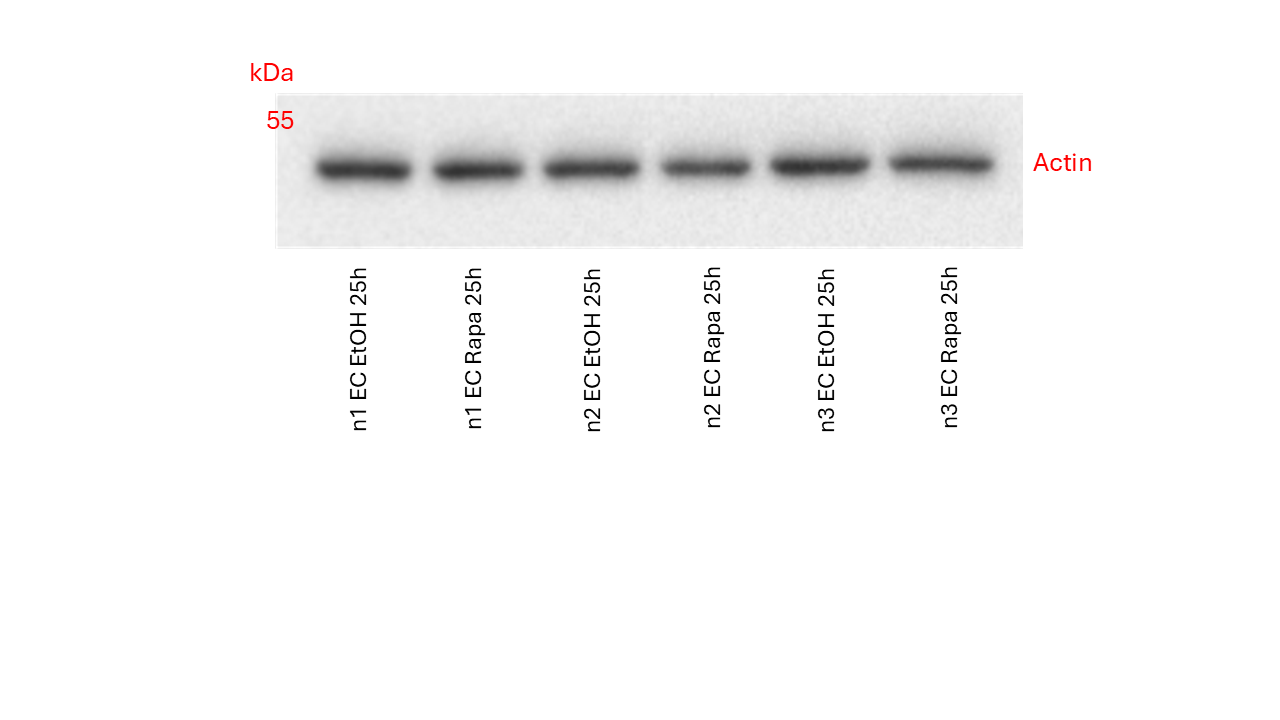

Supplement: Supplementary file 9 — Source data Fig. 5 [file 44319_2025_460_MOESM9_ESM.zip › Figure 5/A/Actin for Wee1.tif]

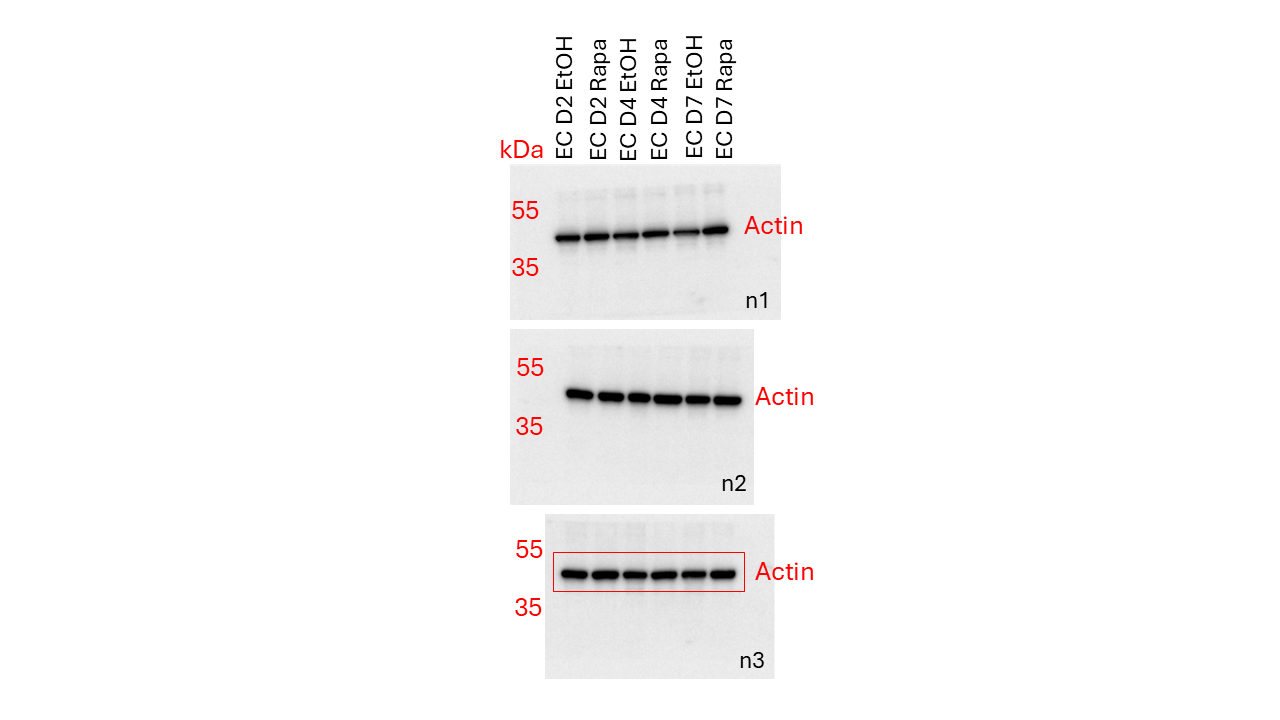

Supplement: Supplementary file 9 — Source data Fig. 5 [file 44319_2025_460_MOESM9_ESM.zip › Figure 5/A/Actins for Cyclin O.tif]

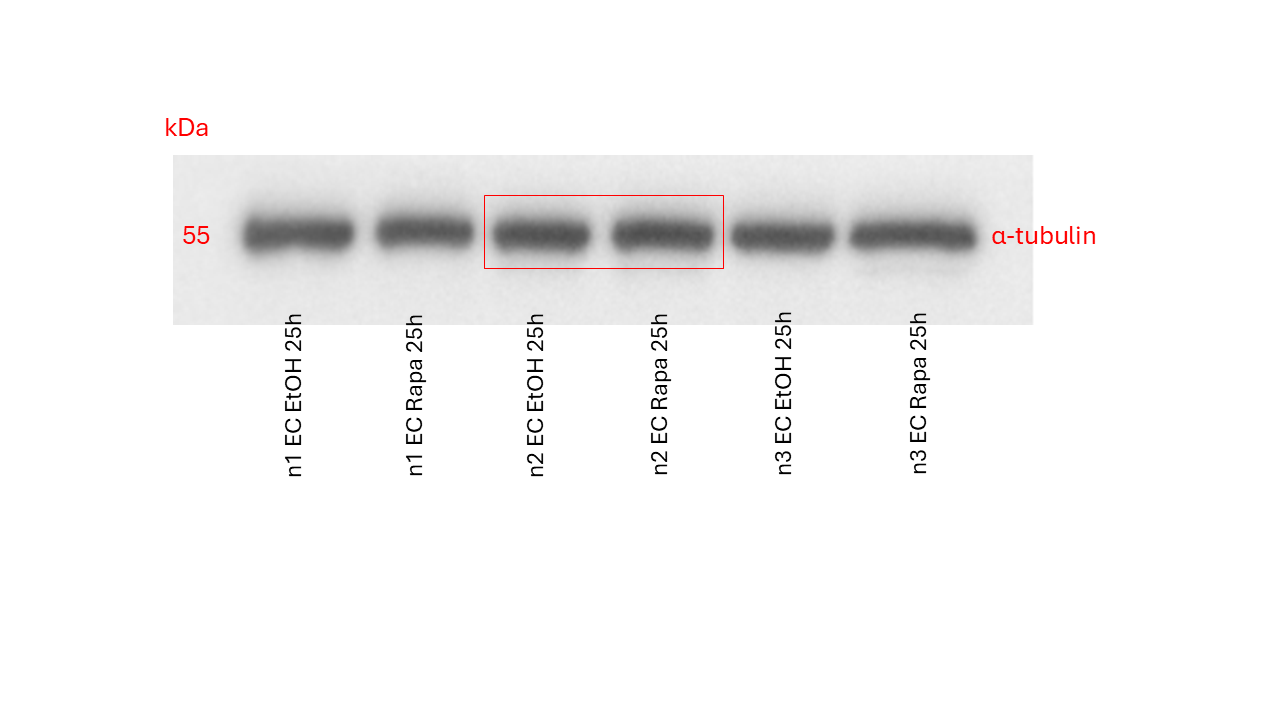

Supplement: Supplementary file 9 — Source data Fig. 5 [file 44319_2025_460_MOESM9_ESM.zip › Figure 5/A/alpha tubulin for p21.tif]

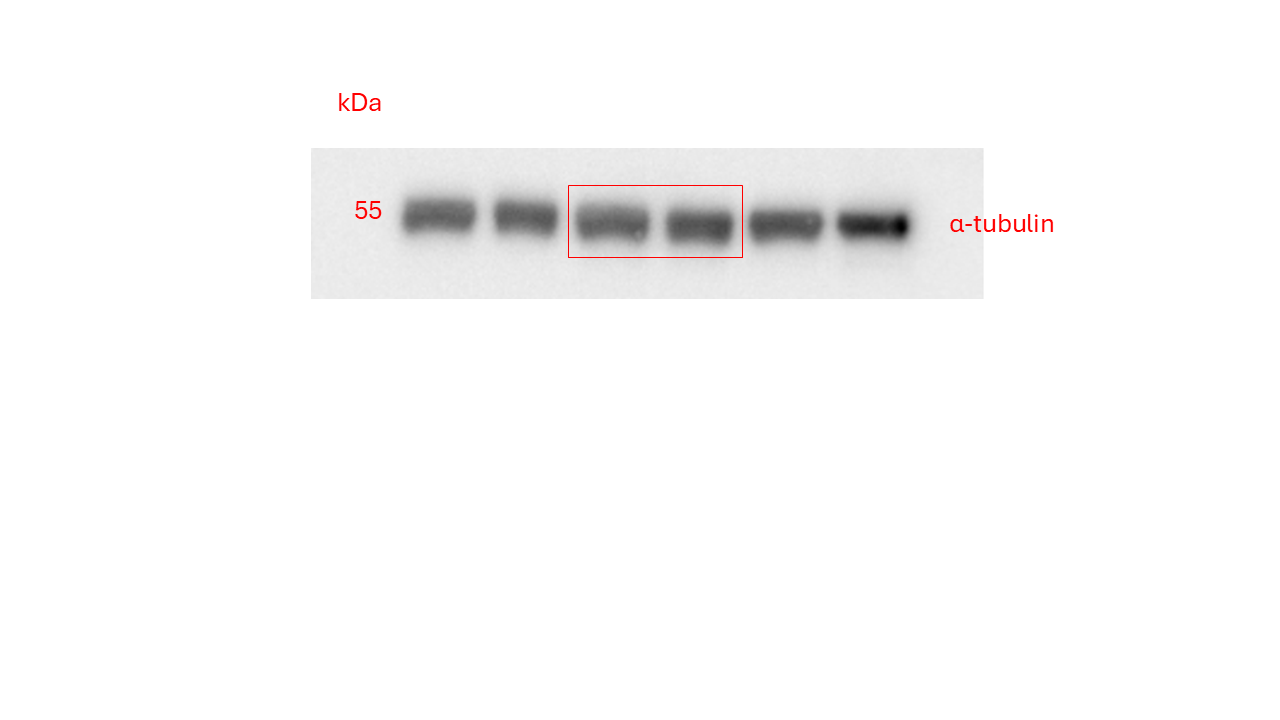

Supplement: Supplementary file 9 — Source data Fig. 5 [file 44319_2025_460_MOESM9_ESM.zip › Figure 5/A/alpha tubulin for pRb Cyclin A and D1.tif]

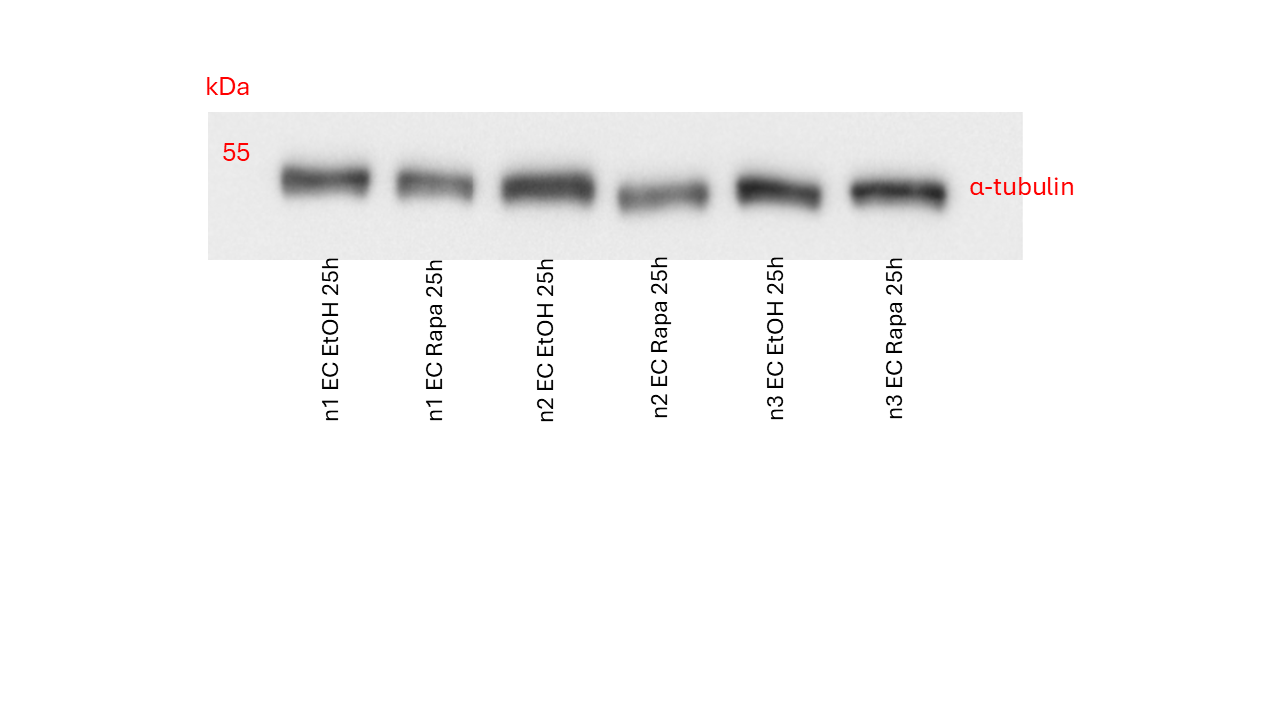

Supplement: Supplementary file 9 — Source data Fig. 5 [file 44319_2025_460_MOESM9_ESM.zip › Figure 5/A/alpha tubulin for Rb.tif]

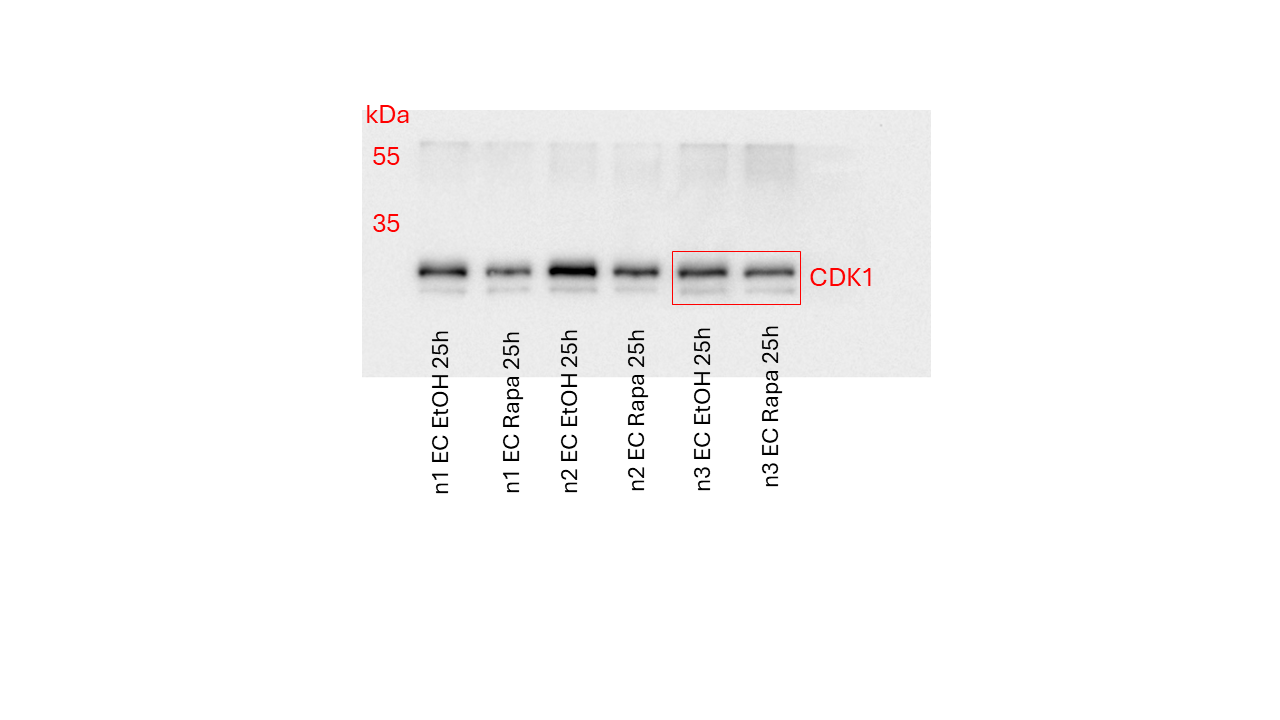

Supplement: Supplementary file 9 — Source data Fig. 5 [file 44319_2025_460_MOESM9_ESM.zip › Figure 5/A/CDK1.tif]

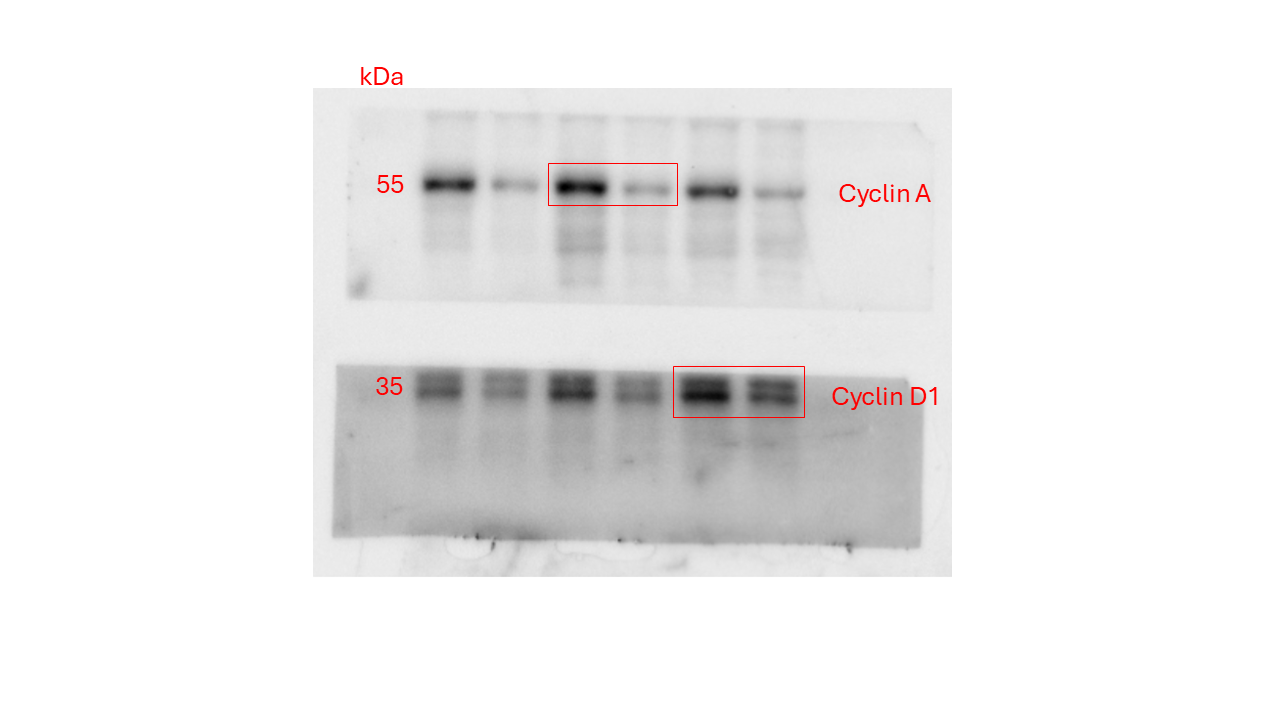

Supplement: Supplementary file 9 — Source data Fig. 5 [file 44319_2025_460_MOESM9_ESM.zip › Figure 5/A/cyclin A and D1.tif]

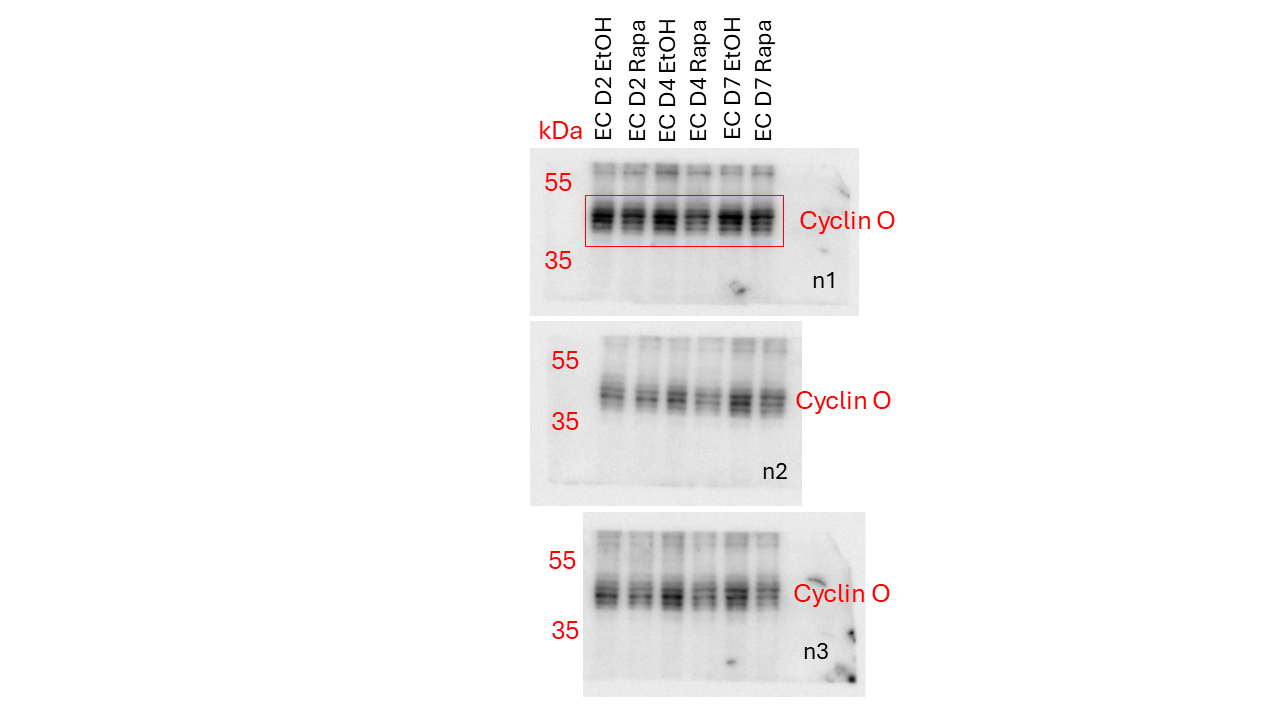

Supplement: Supplementary file 9 — Source data Fig. 5 [file 44319_2025_460_MOESM9_ESM.zip › Figure 5/A/cyclin O.tif]

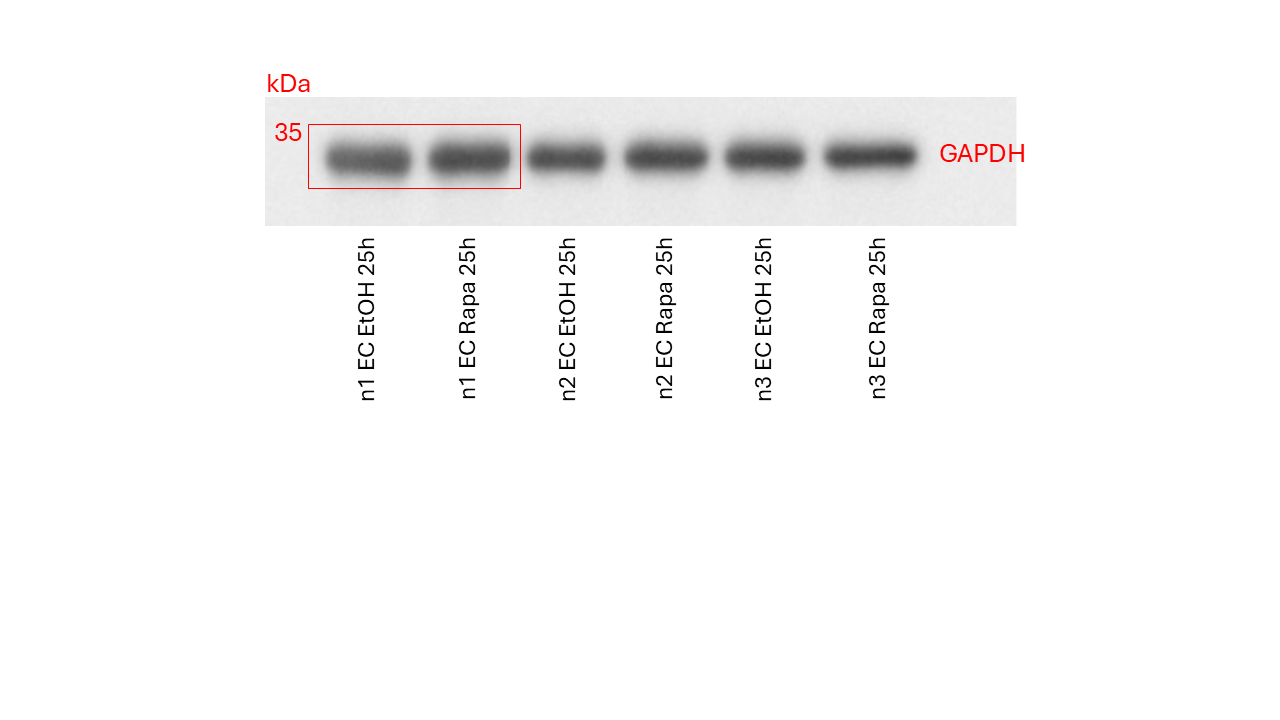

Supplement: Supplementary file 9 — Source data Fig. 5 [file 44319_2025_460_MOESM9_ESM.zip › Figure 5/A/GAPDH for p53.tif]

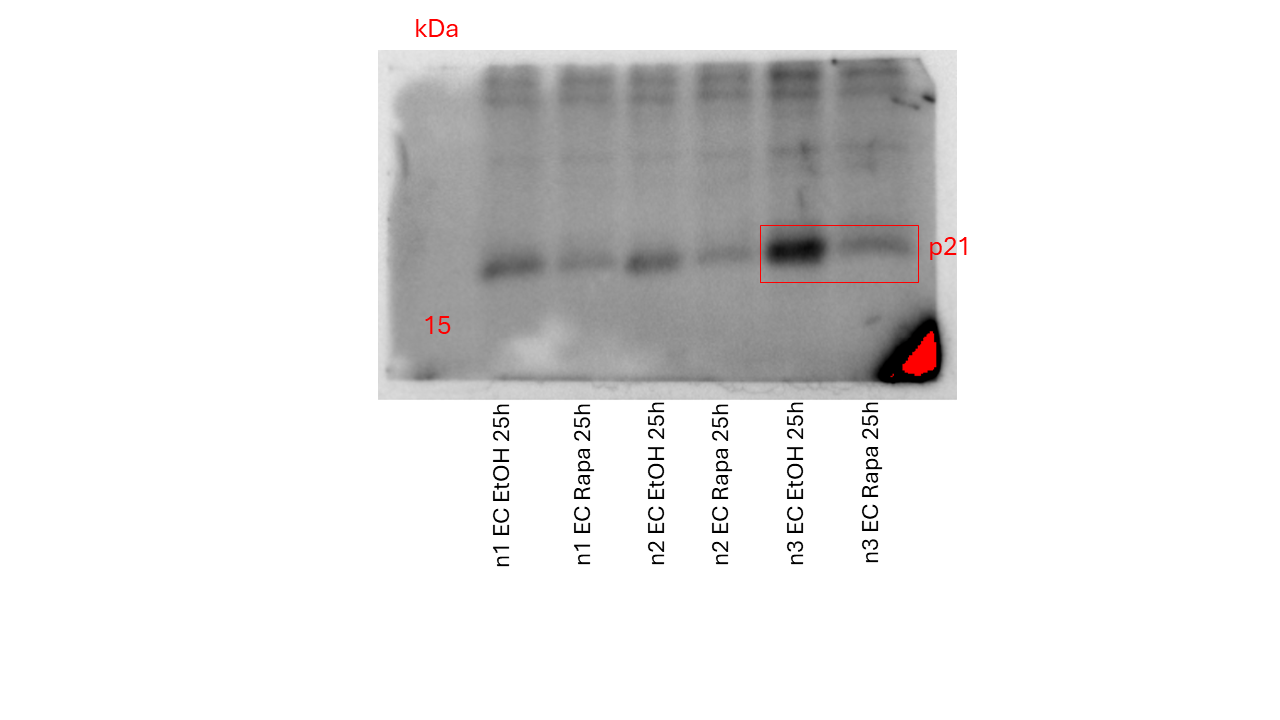

Supplement: Supplementary file 9 — Source data Fig. 5 [file 44319_2025_460_MOESM9_ESM.zip › Figure 5/A/p21.tif]

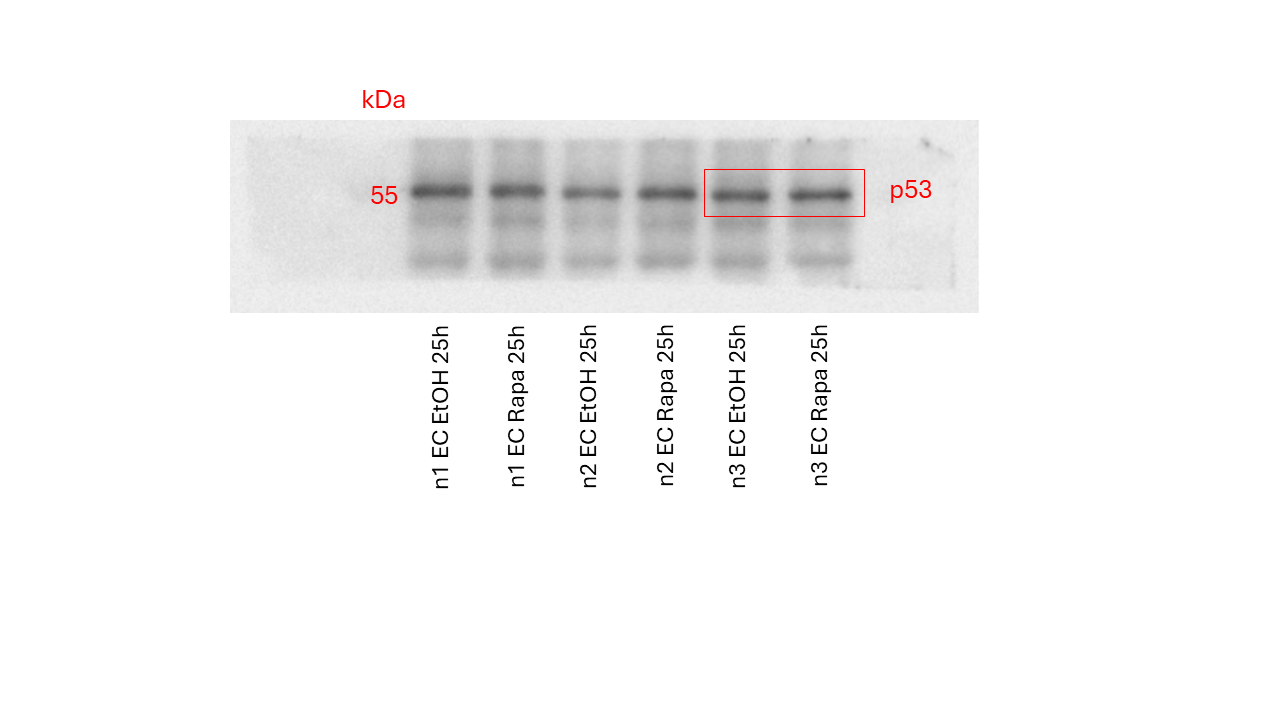

Supplement: Supplementary file 9 — Source data Fig. 5 [file 44319_2025_460_MOESM9_ESM.zip › Figure 5/A/p53.tif]

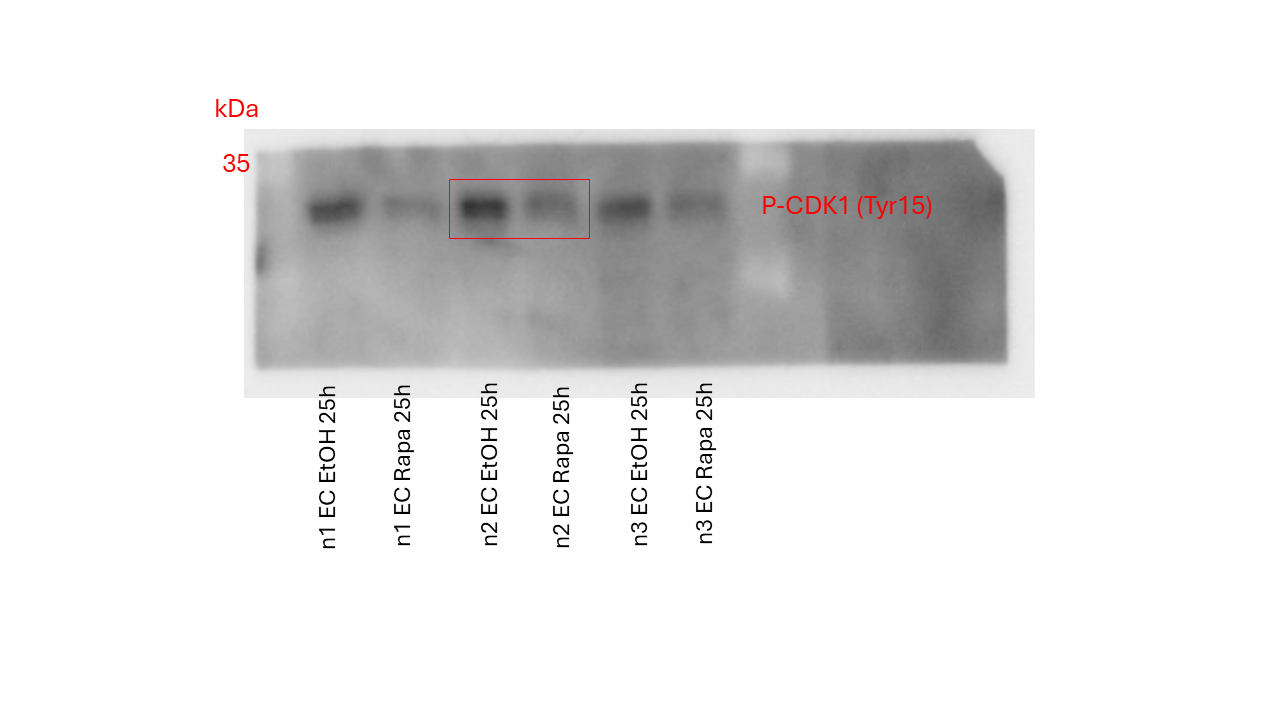

Supplement: Supplementary file 9 — Source data Fig. 5 [file 44319_2025_460_MOESM9_ESM.zip › Figure 5/A/pCDK1.tif]

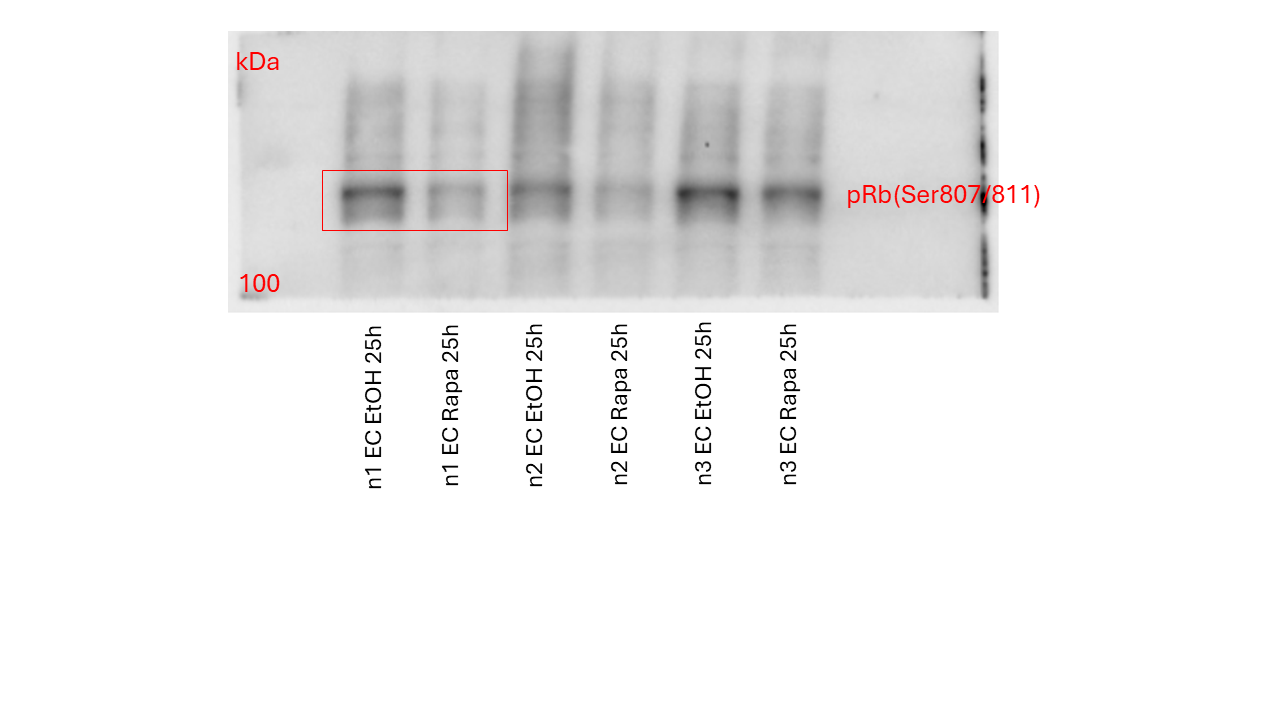

Supplement: Supplementary file 9 — Source data Fig. 5 [file 44319_2025_460_MOESM9_ESM.zip › Figure 5/A/pRb.tif]

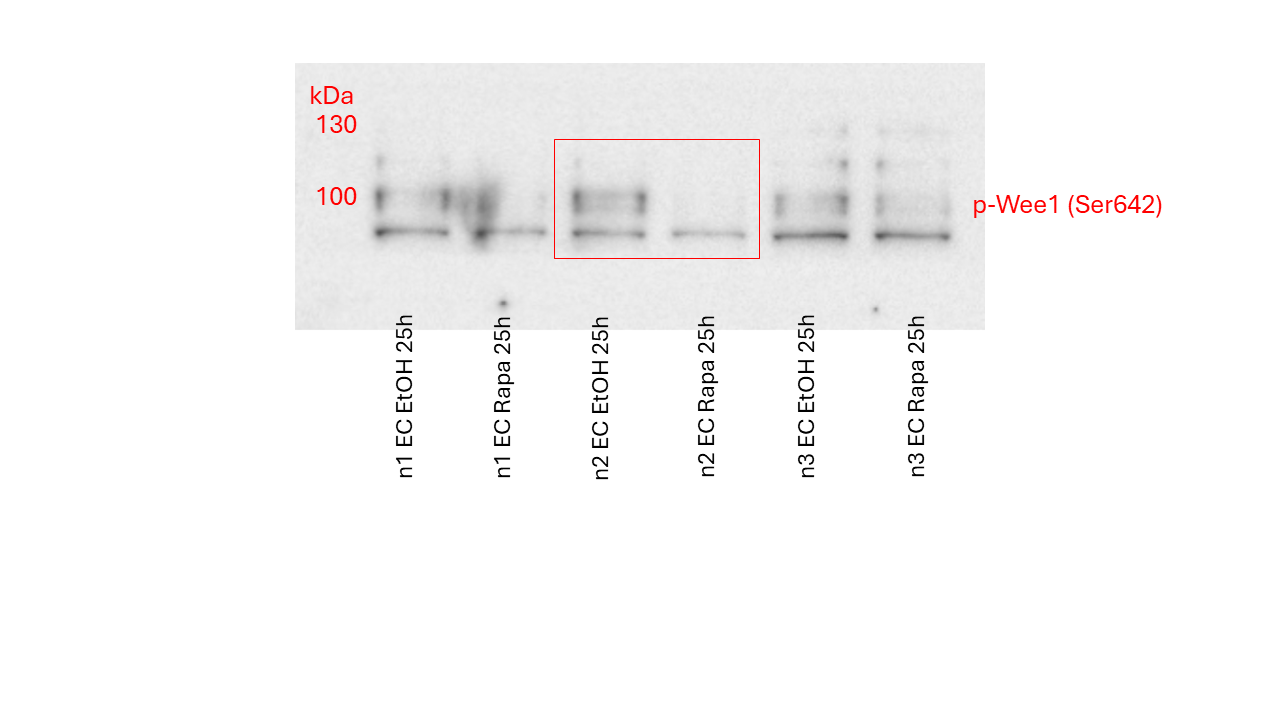

Supplement: Supplementary file 9 — Source data Fig. 5 [file 44319_2025_460_MOESM9_ESM.zip › Figure 5/A/pWee1.tif]

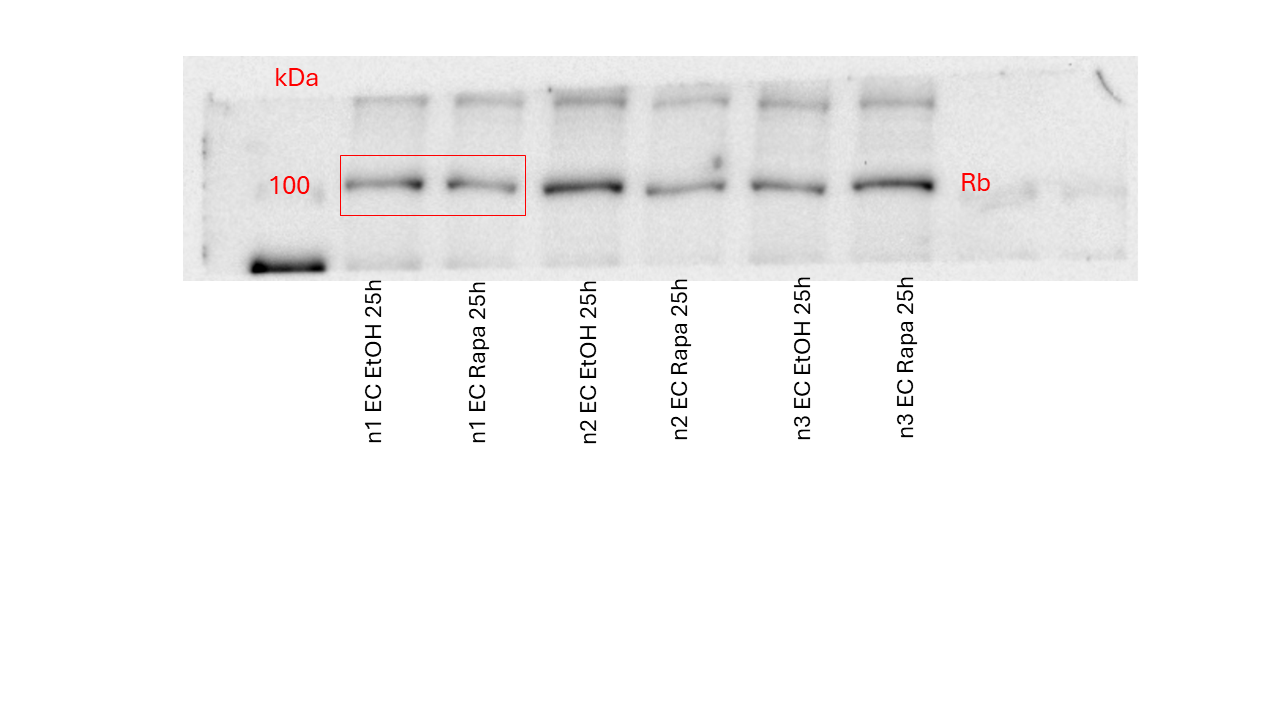

Supplement: Supplementary file 9 — Source data Fig. 5 [file 44319_2025_460_MOESM9_ESM.zip › Figure 5/A/Rb.tif]

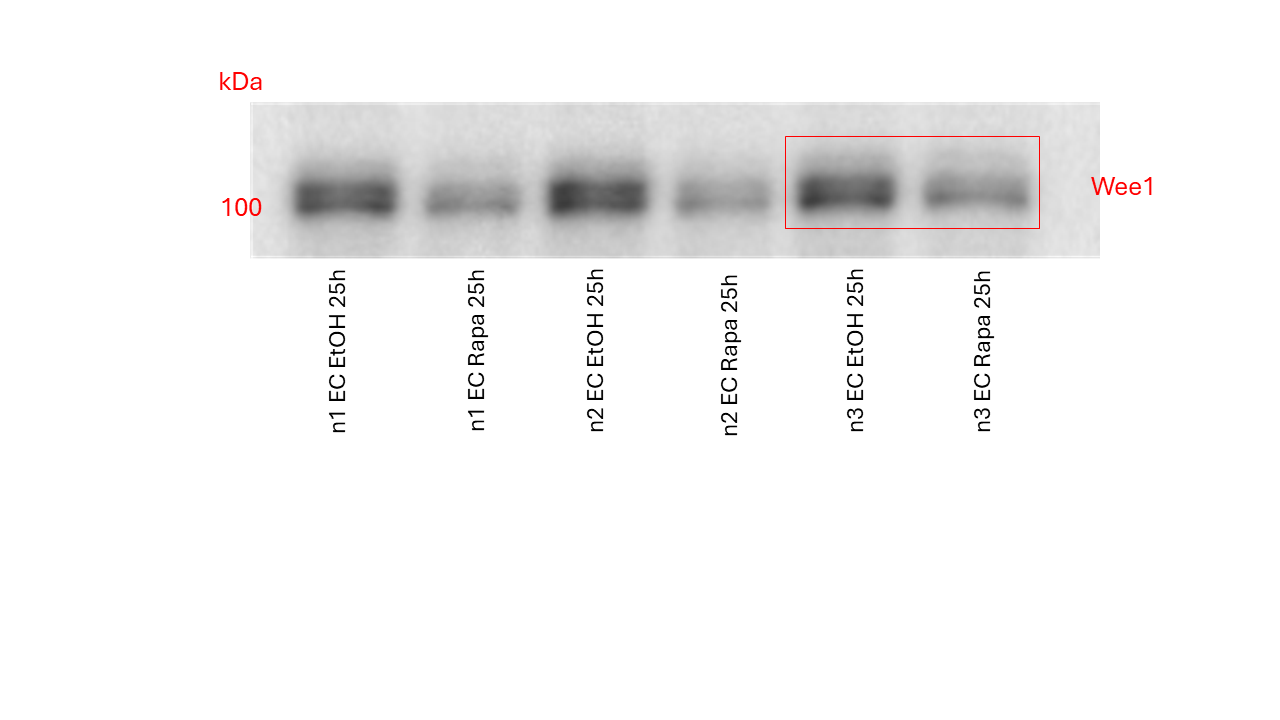

Supplement: Supplementary file 9 — Source data Fig. 5 [file 44319_2025_460_MOESM9_ESM.zip › Figure 5/A/Wee1.tif]

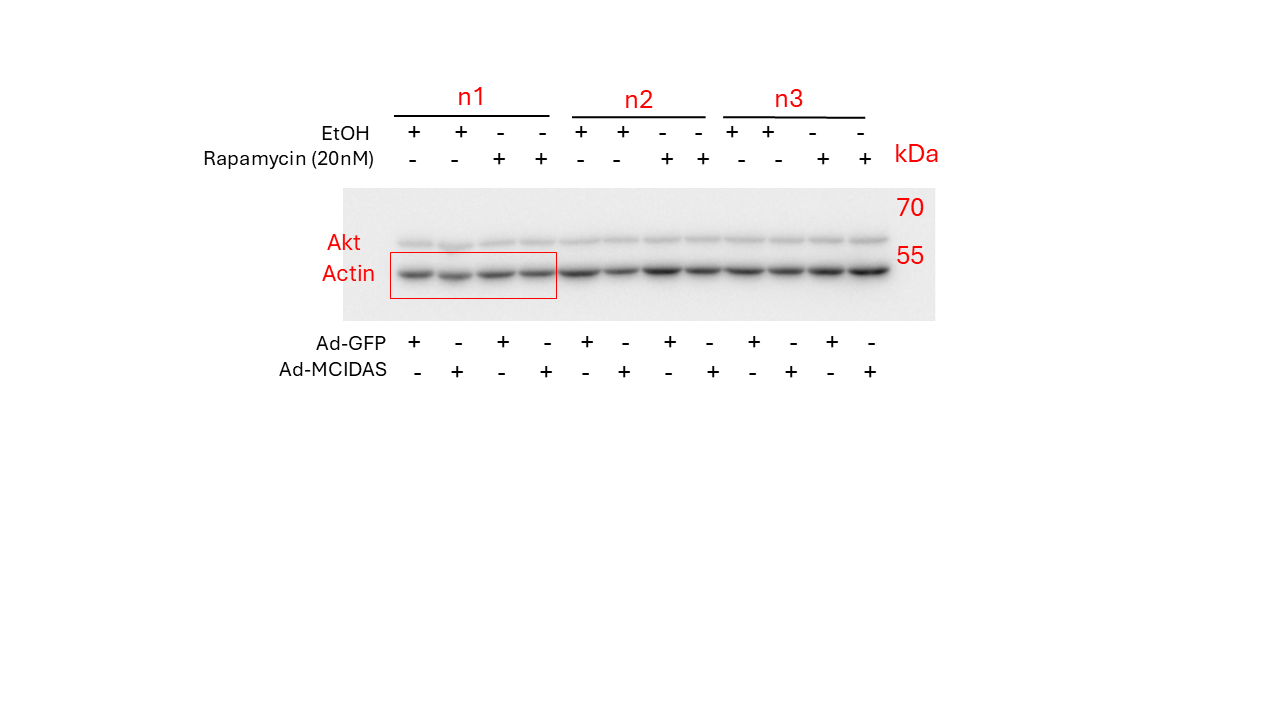

Supplement: Supplementary file 10 — Source data Fig. 6 [file 44319_2025_460_MOESM10_ESM.zip › Figure 6/A/Actin for Akt.tif]

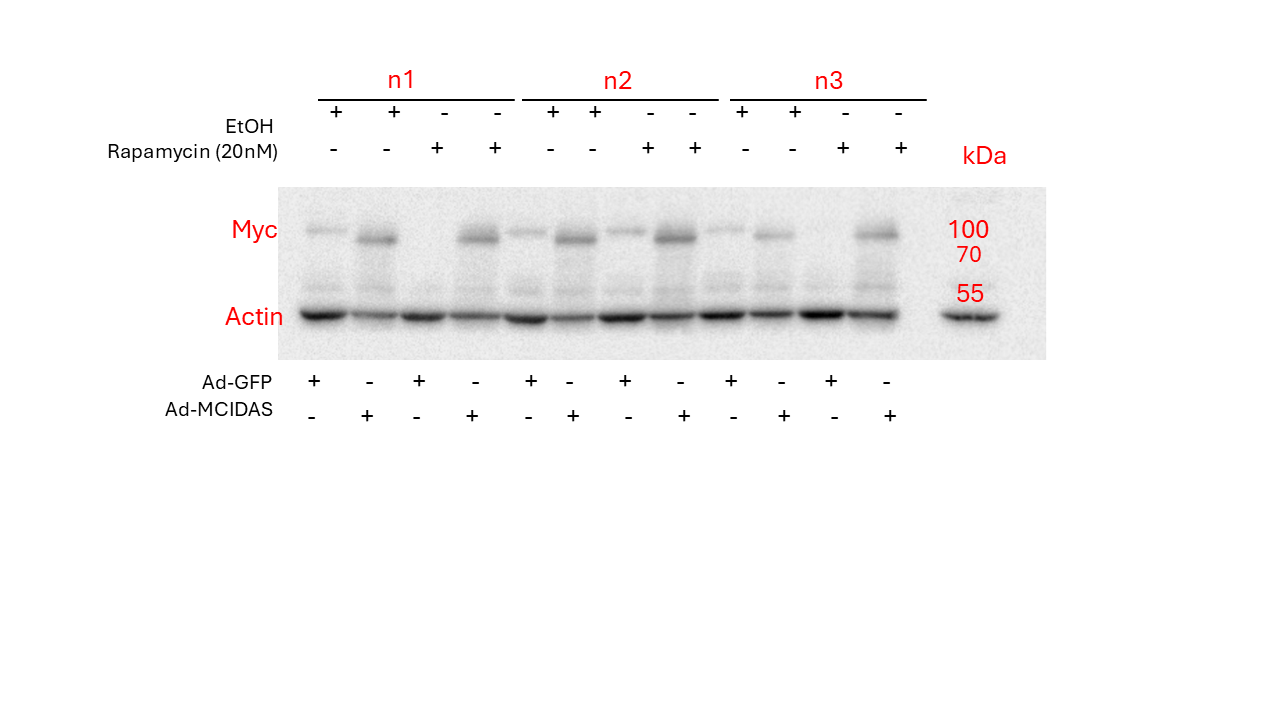

Supplement: Supplementary file 10 — Source data Fig. 6 [file 44319_2025_460_MOESM10_ESM.zip › Figure 6/A/Actin for Myc and rpS6.tif]

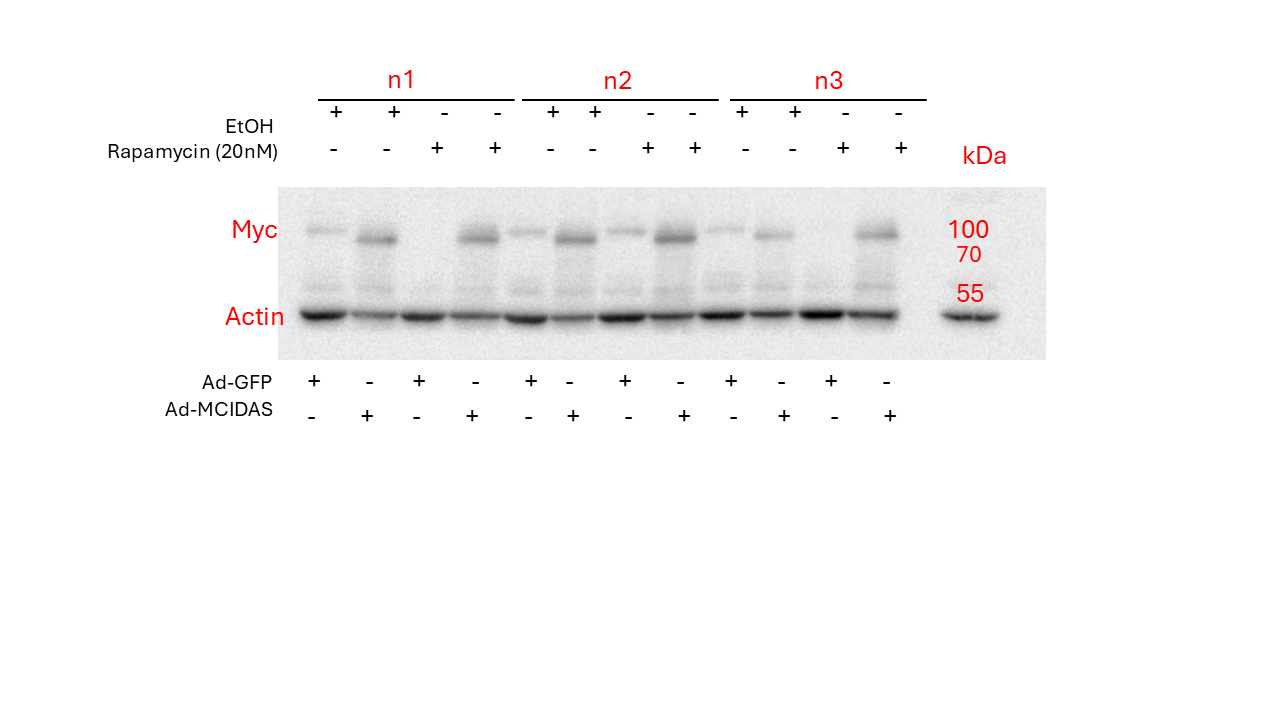

Supplement: Supplementary file 10 — Source data Fig. 6 [file 44319_2025_460_MOESM10_ESM.zip › Figure 6/A/Actin for Myc.tif]

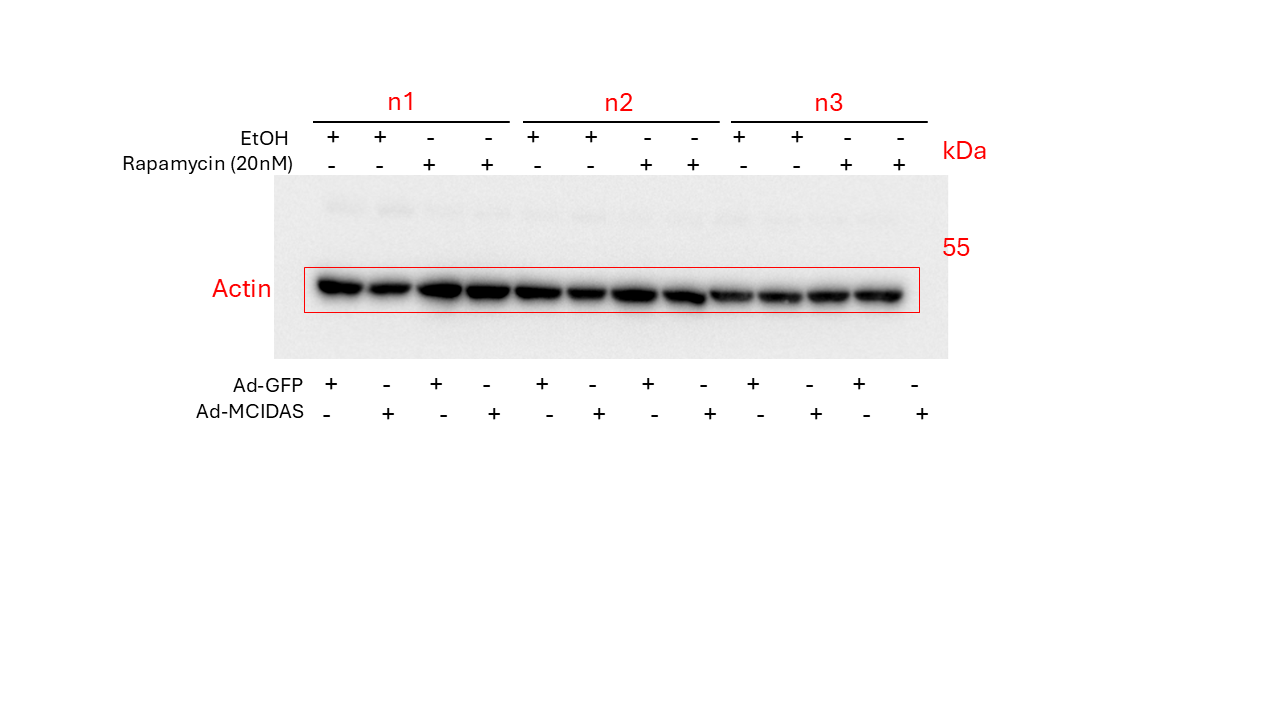

Supplement: Supplementary file 10 — Source data Fig. 6 [file 44319_2025_460_MOESM10_ESM.zip › Figure 6/A/Actin for pCAD.tif]

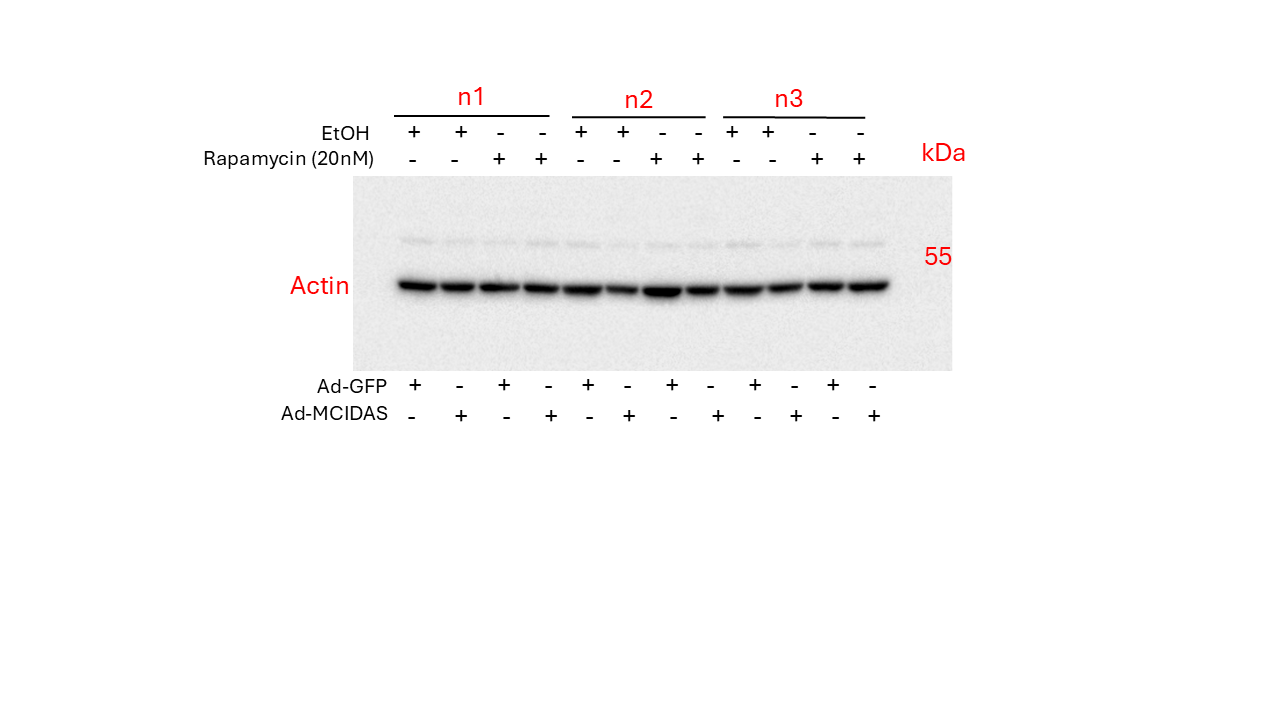

Supplement: Supplementary file 10 — Source data Fig. 6 [file 44319_2025_460_MOESM10_ESM.zip › Figure 6/A/Actin for prpS6 and pAkt.tif]

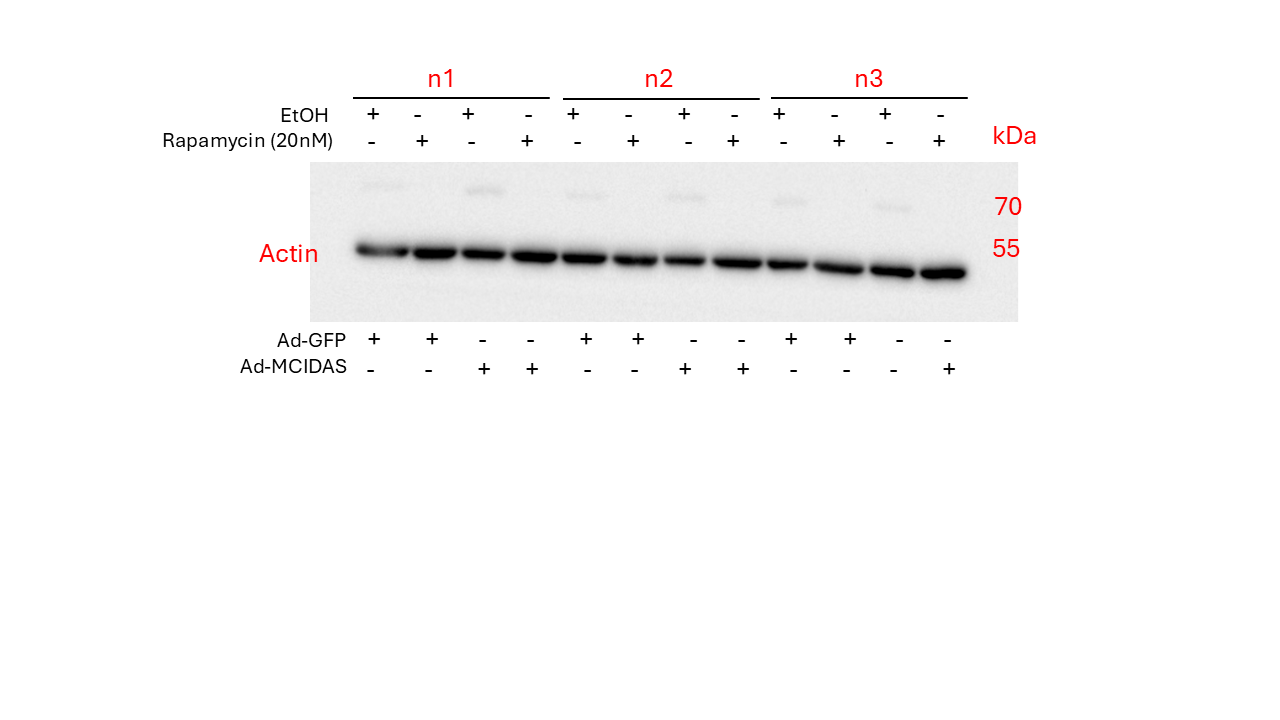

Supplement: Supplementary file 10 — Source data Fig. 6 [file 44319_2025_460_MOESM10_ESM.zip › Figure 6/A/Actin for pS6K1.tif]

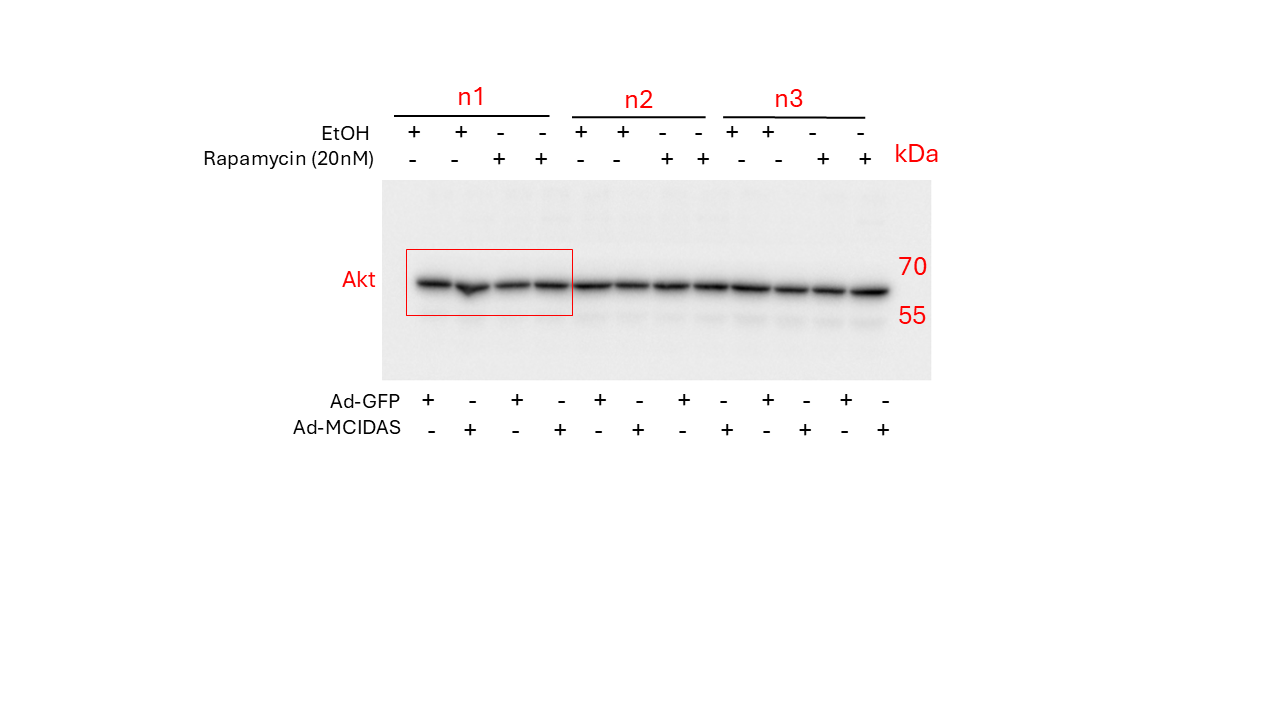

Supplement: Supplementary file 10 — Source data Fig. 6 [file 44319_2025_460_MOESM10_ESM.zip › Figure 6/A/Akt.tif]

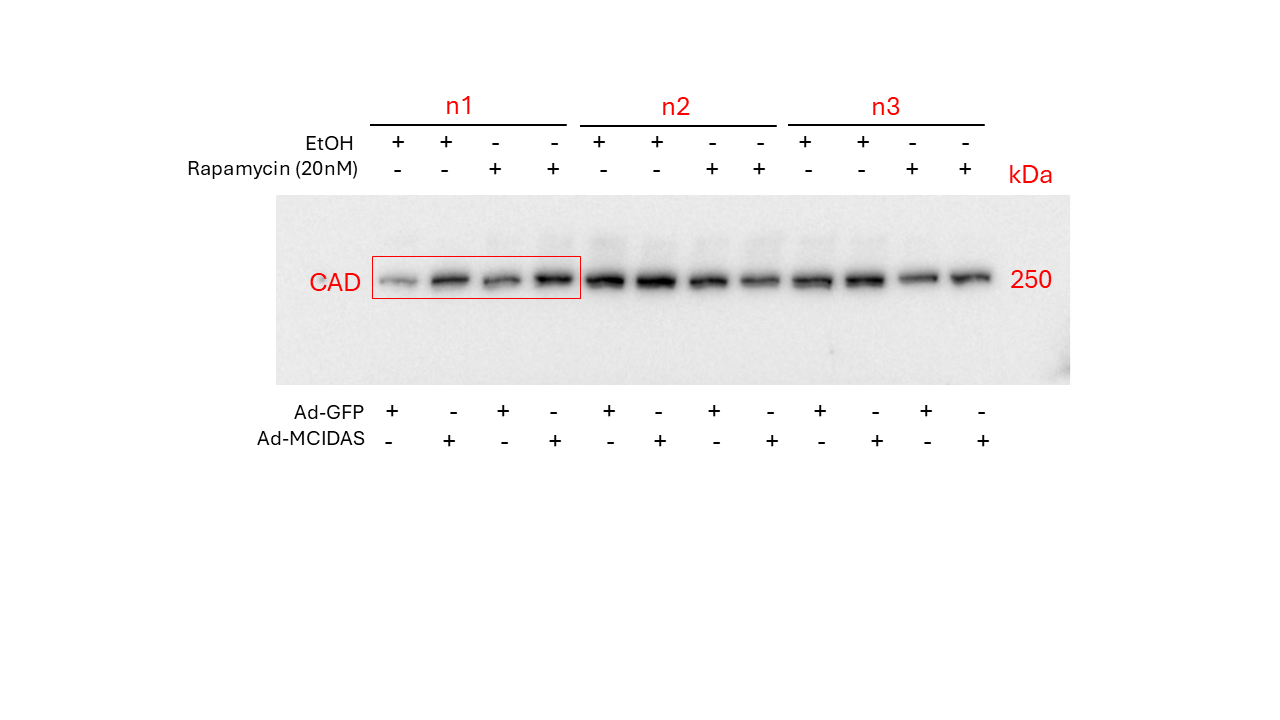

Supplement: Supplementary file 10 — Source data Fig. 6 [file 44319_2025_460_MOESM10_ESM.zip › Figure 6/A/CAD.tif]

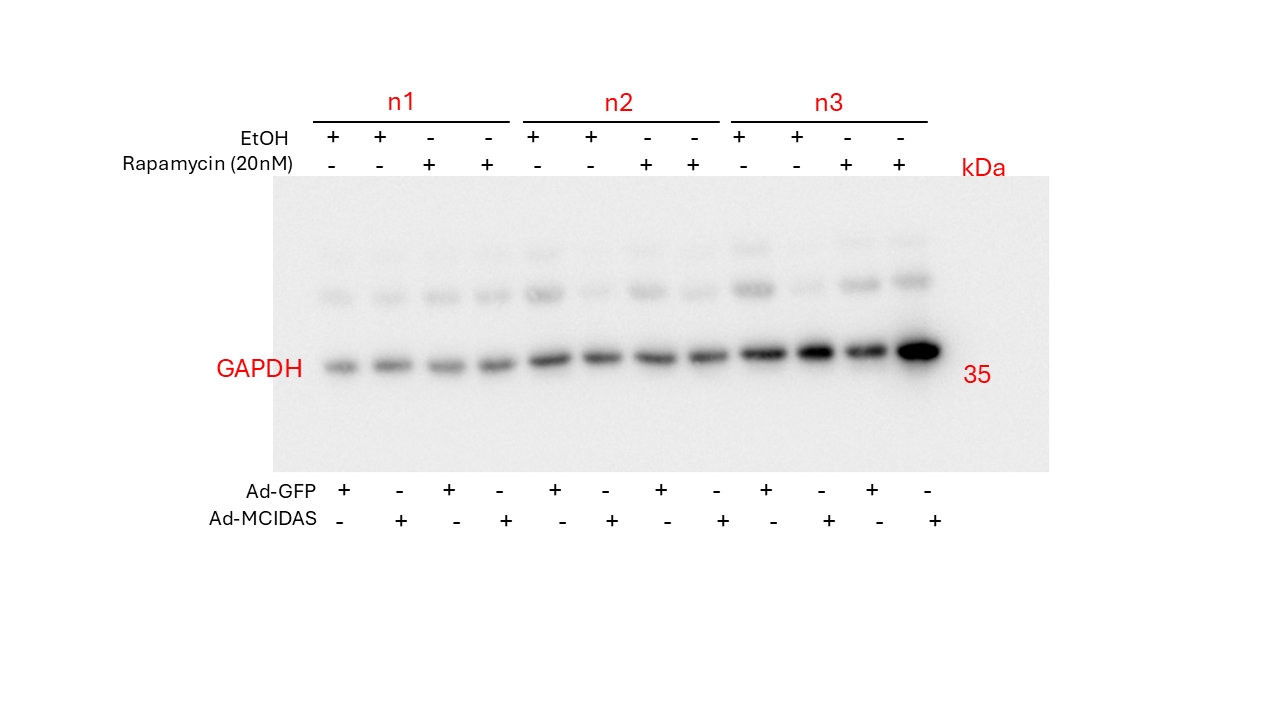

Supplement: Supplementary file 10 — Source data Fig. 6 [file 44319_2025_460_MOESM10_ESM.zip › Figure 6/A/GAPDH for CAD S6K1 GSK3 alpha and beta.tif]

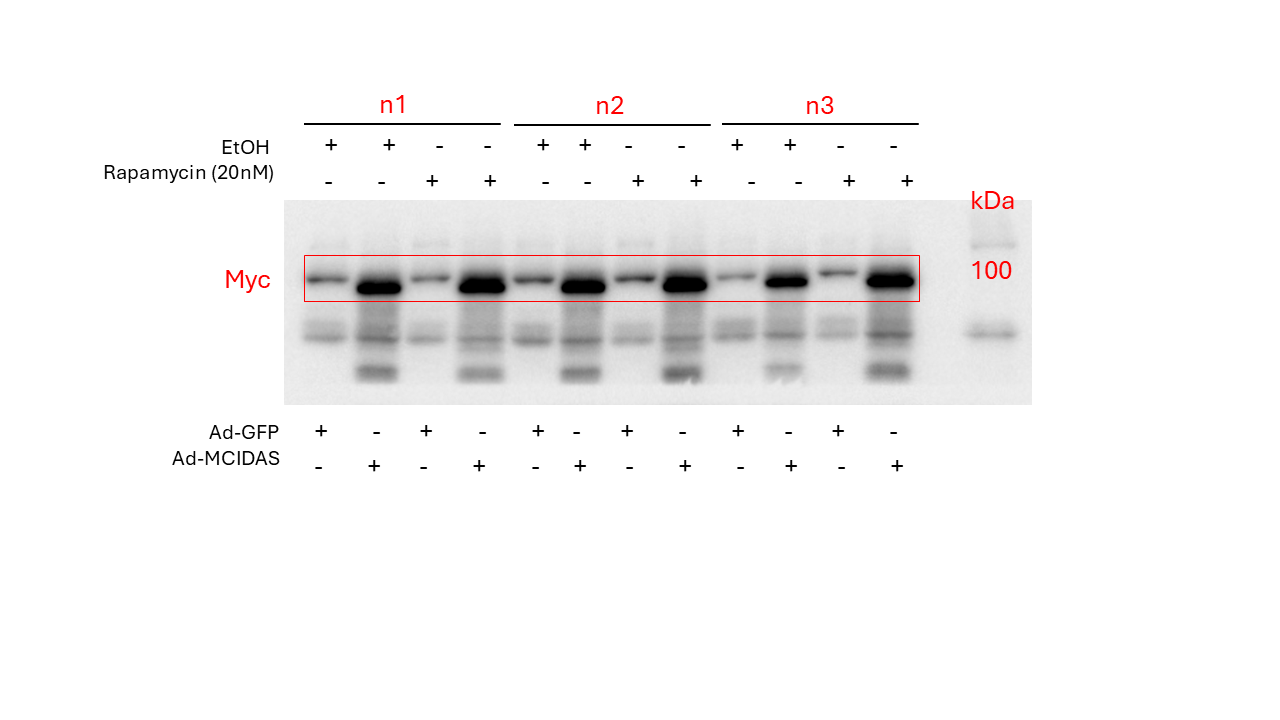

Supplement: Supplementary file 10 — Source data Fig. 6 [file 44319_2025_460_MOESM10_ESM.zip › Figure 6/A/Myc.tif]

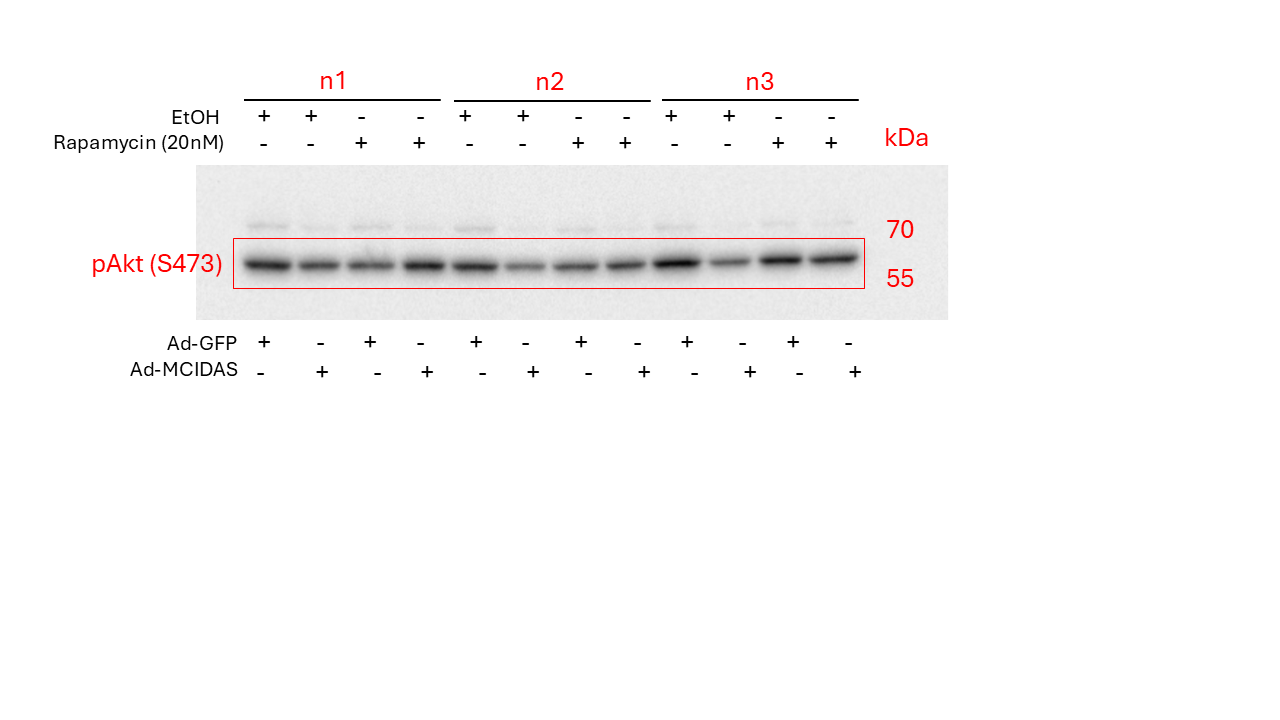

Supplement: Supplementary file 10 — Source data Fig. 6 [file 44319_2025_460_MOESM10_ESM.zip › Figure 6/A/pAkt.tif]

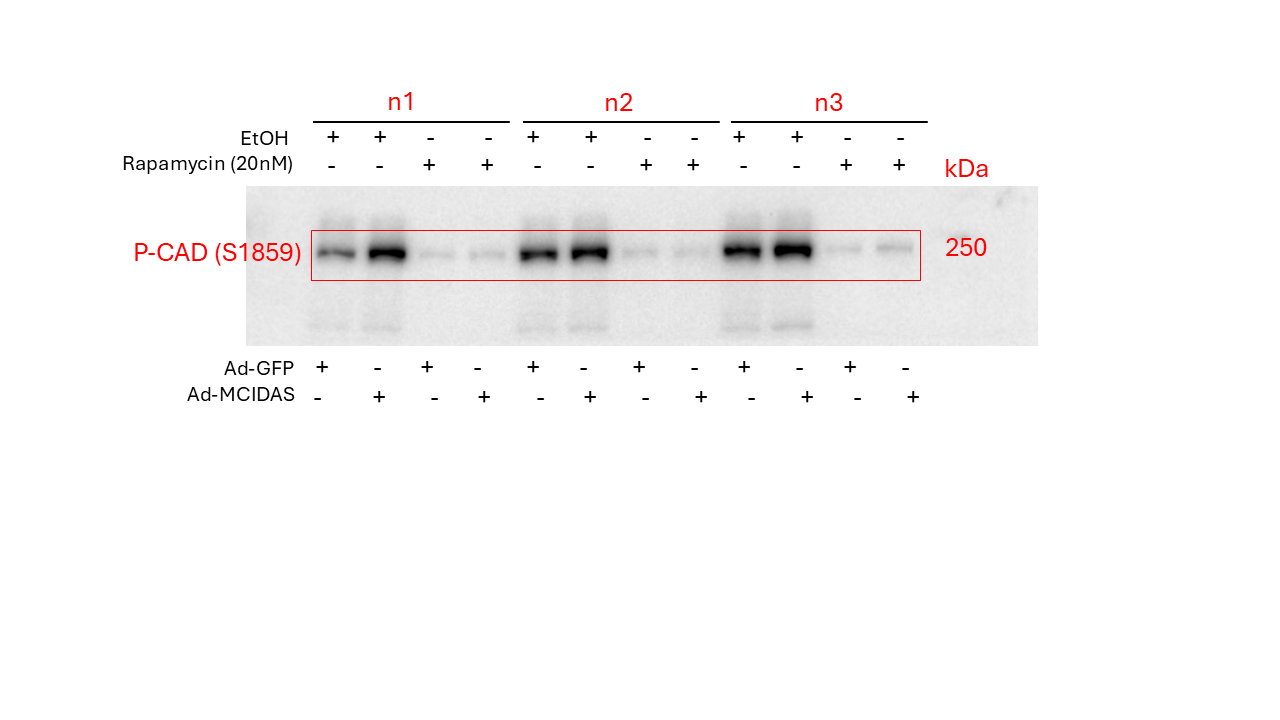

Supplement: Supplementary file 10 — Source data Fig. 6 [file 44319_2025_460_MOESM10_ESM.zip › Figure 6/A/pCAD.tif]

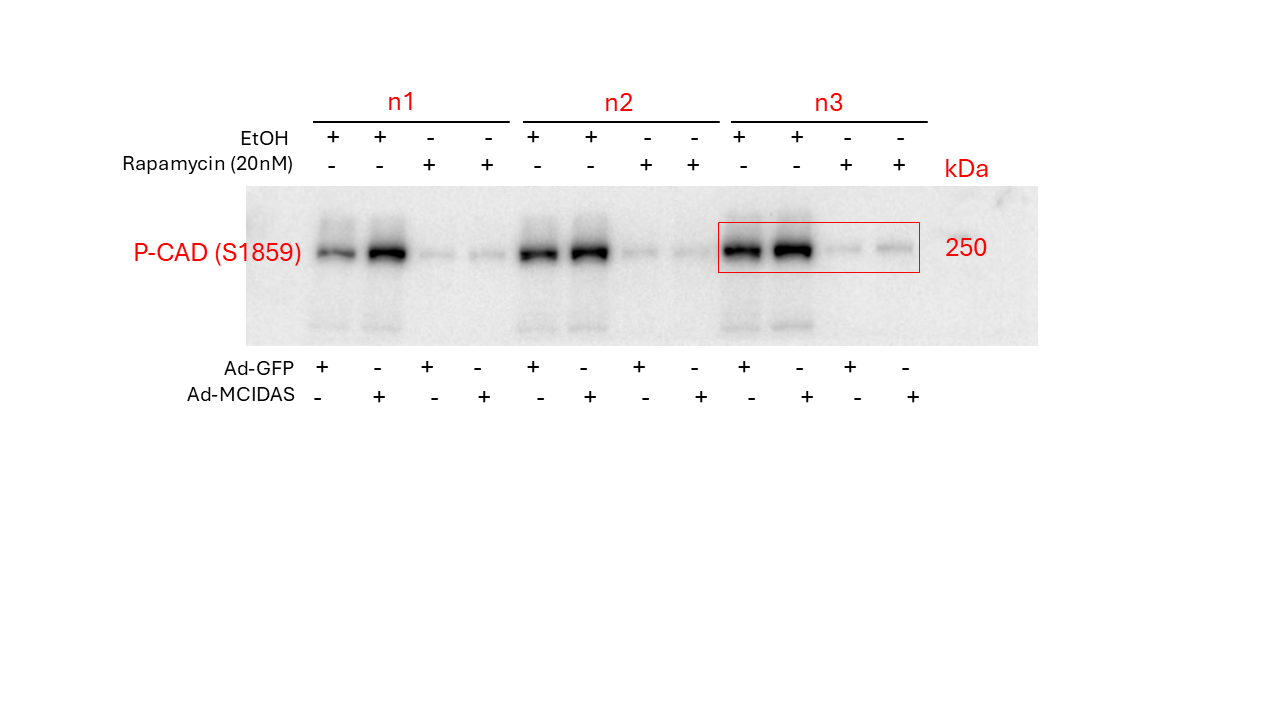

Supplement: Supplementary file 10 — Source data Fig. 6 [file 44319_2025_460_MOESM10_ESM.zip › Figure 6/A/pCAD_.tif]

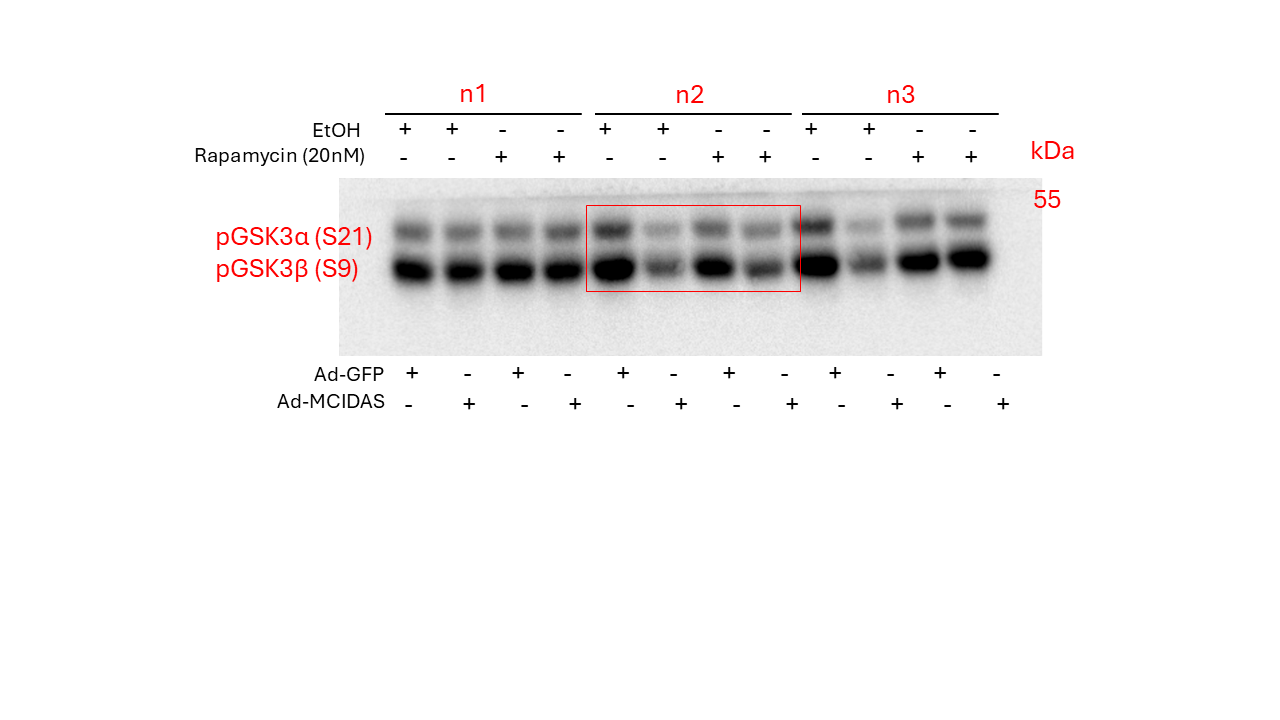

Supplement: Supplementary file 10 — Source data Fig. 6 [file 44319_2025_460_MOESM10_ESM.zip › Figure 6/A/pGSK3alpha and beta(1).tif]

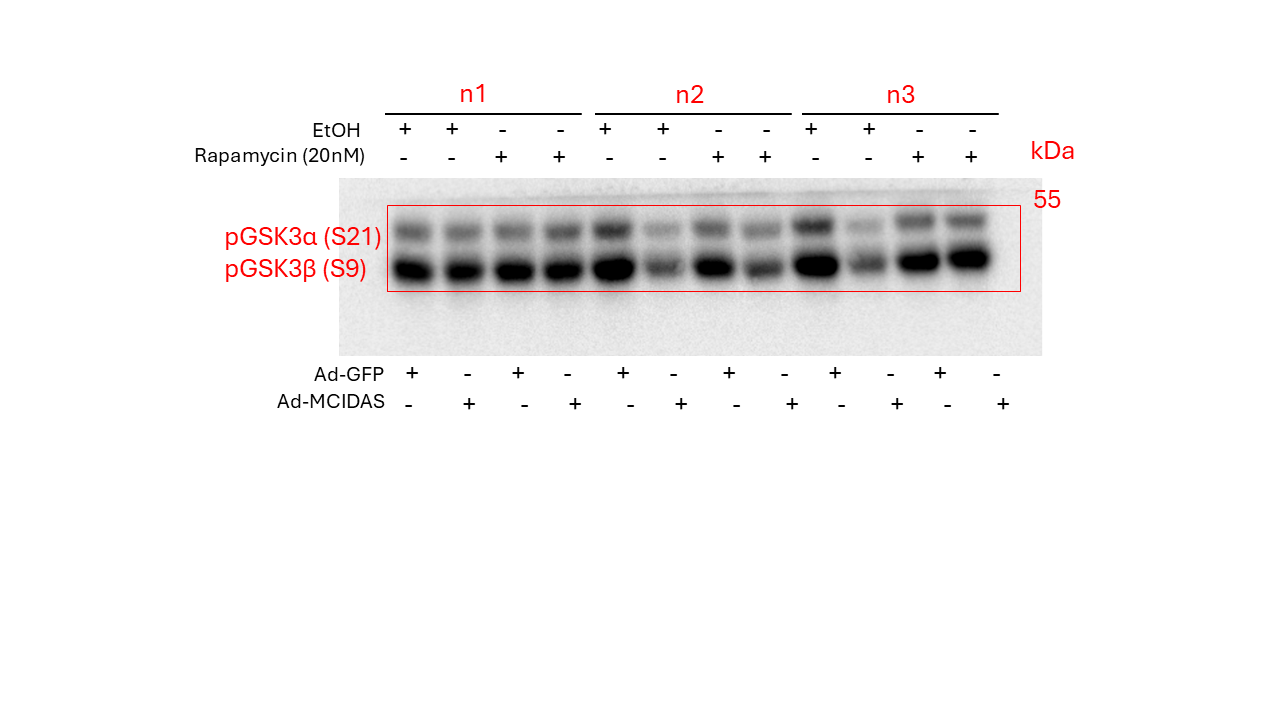

Supplement: Supplementary file 10 — Source data Fig. 6 [file 44319_2025_460_MOESM10_ESM.zip › Figure 6/A/pGSK3alpha and beta.tif]

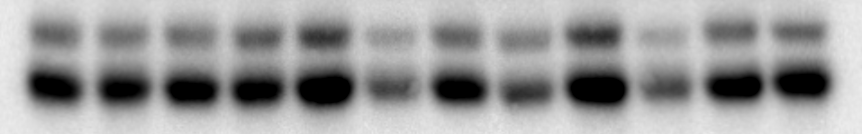

Supplement: Supplementary file 10 — Source data Fig. 6 [file 44319_2025_460_MOESM10_ESM.zip › Figure 6/A/pgsk3_PendeLab 2024-02-22 17h40m54s.tif]

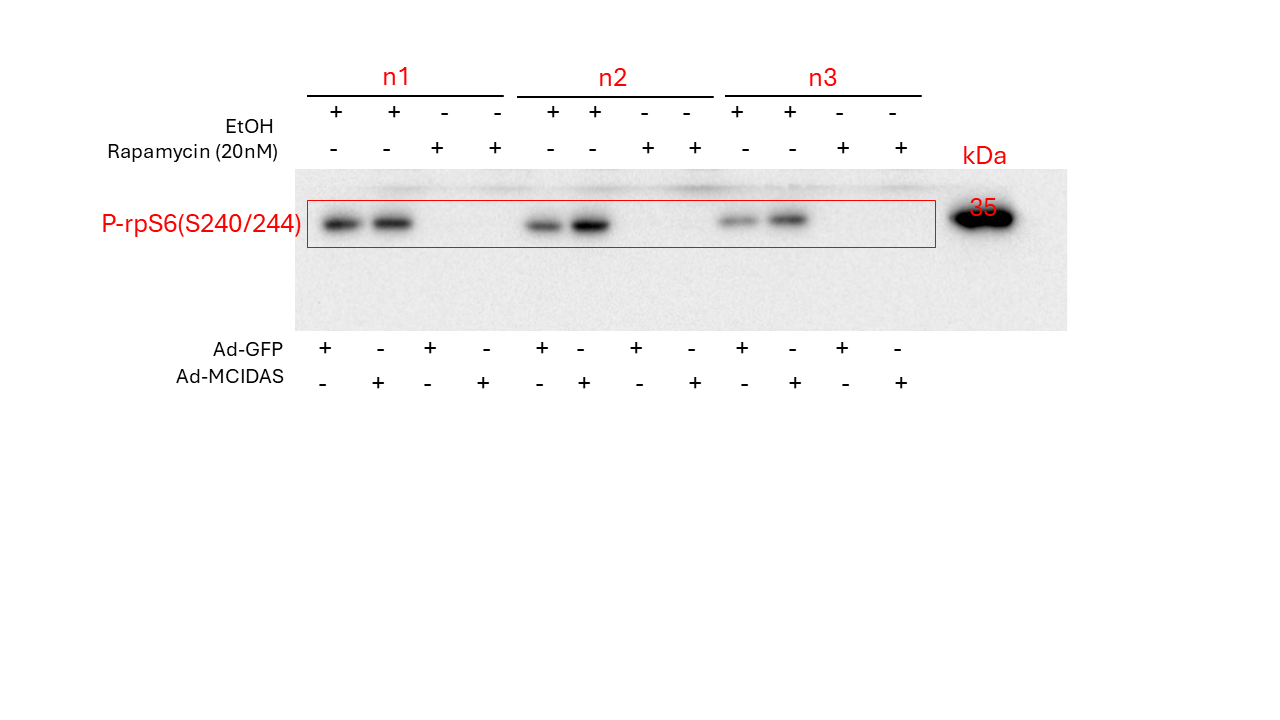

Supplement: Supplementary file 10 — Source data Fig. 6 [file 44319_2025_460_MOESM10_ESM.zip › Figure 6/A/prpS6.tif]

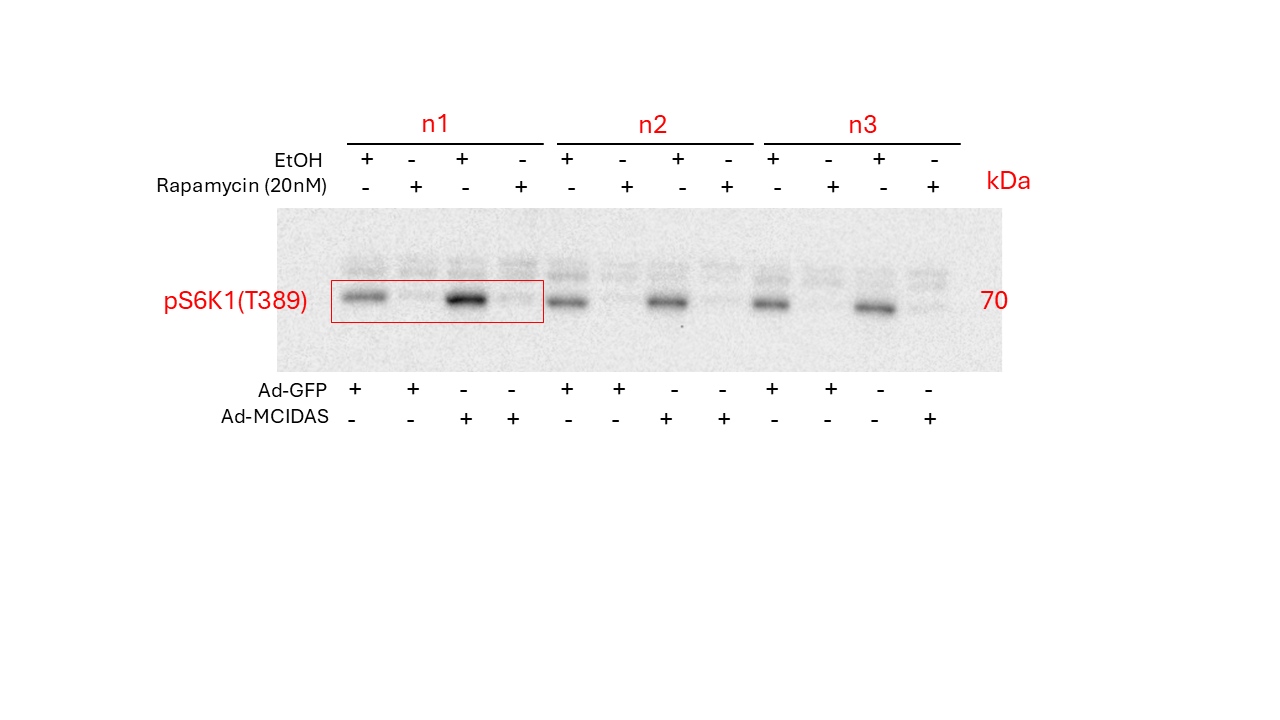

Supplement: Supplementary file 10 — Source data Fig. 6 [file 44319_2025_460_MOESM10_ESM.zip › Figure 6/A/pS6K1.tif]

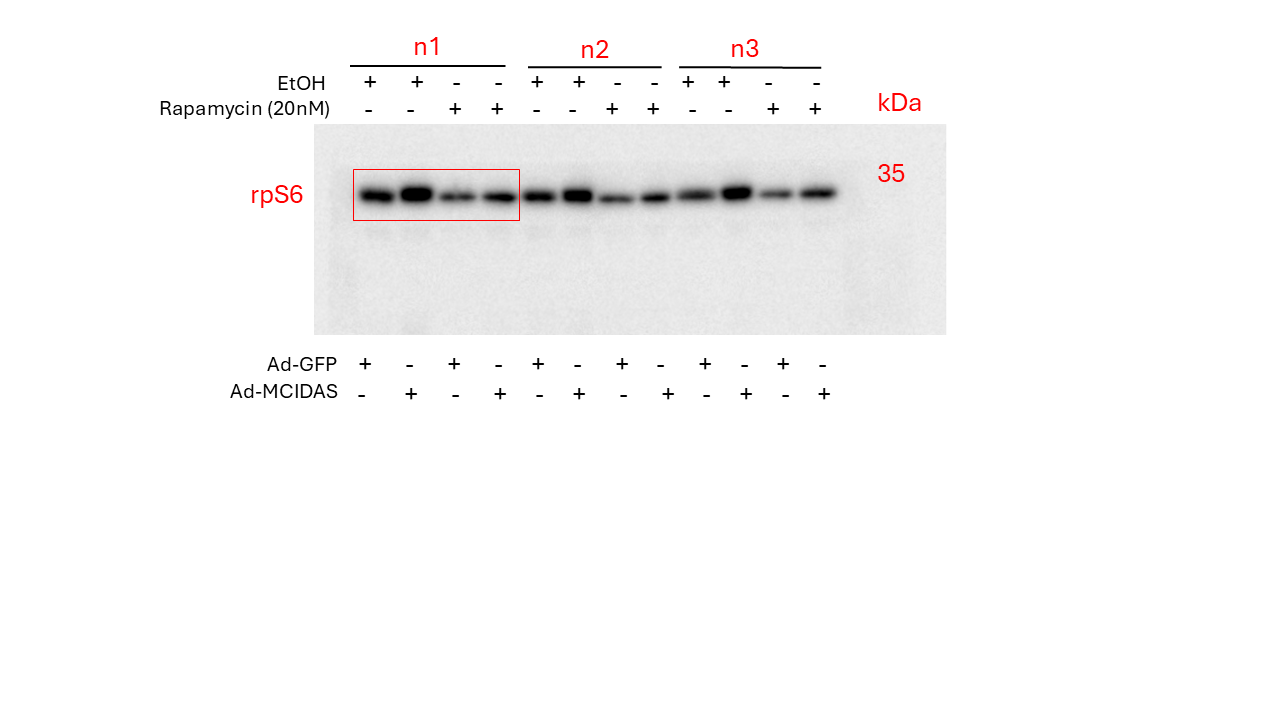

Supplement: Supplementary file 10 — Source data Fig. 6 [file 44319_2025_460_MOESM10_ESM.zip › Figure 6/A/rpS6.tif]

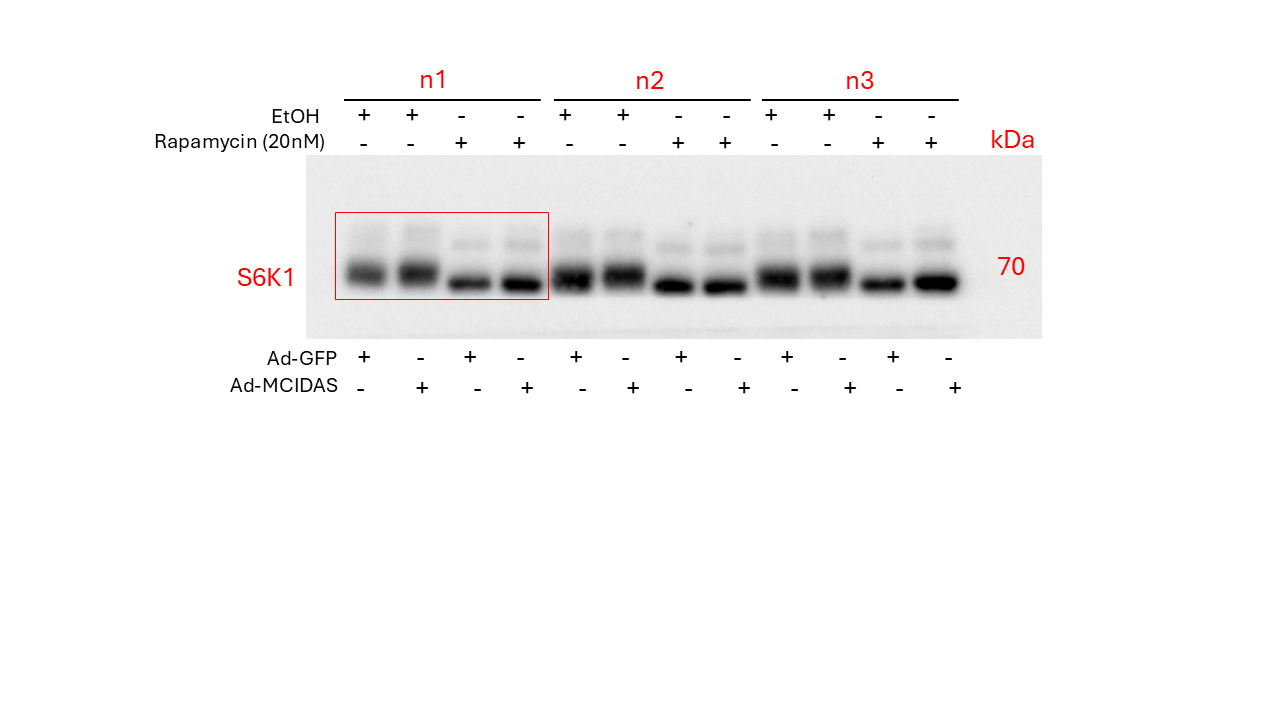

Supplement: Supplementary file 10 — Source data Fig. 6 [file 44319_2025_460_MOESM10_ESM.zip › Figure 6/A/S6K1.tif]

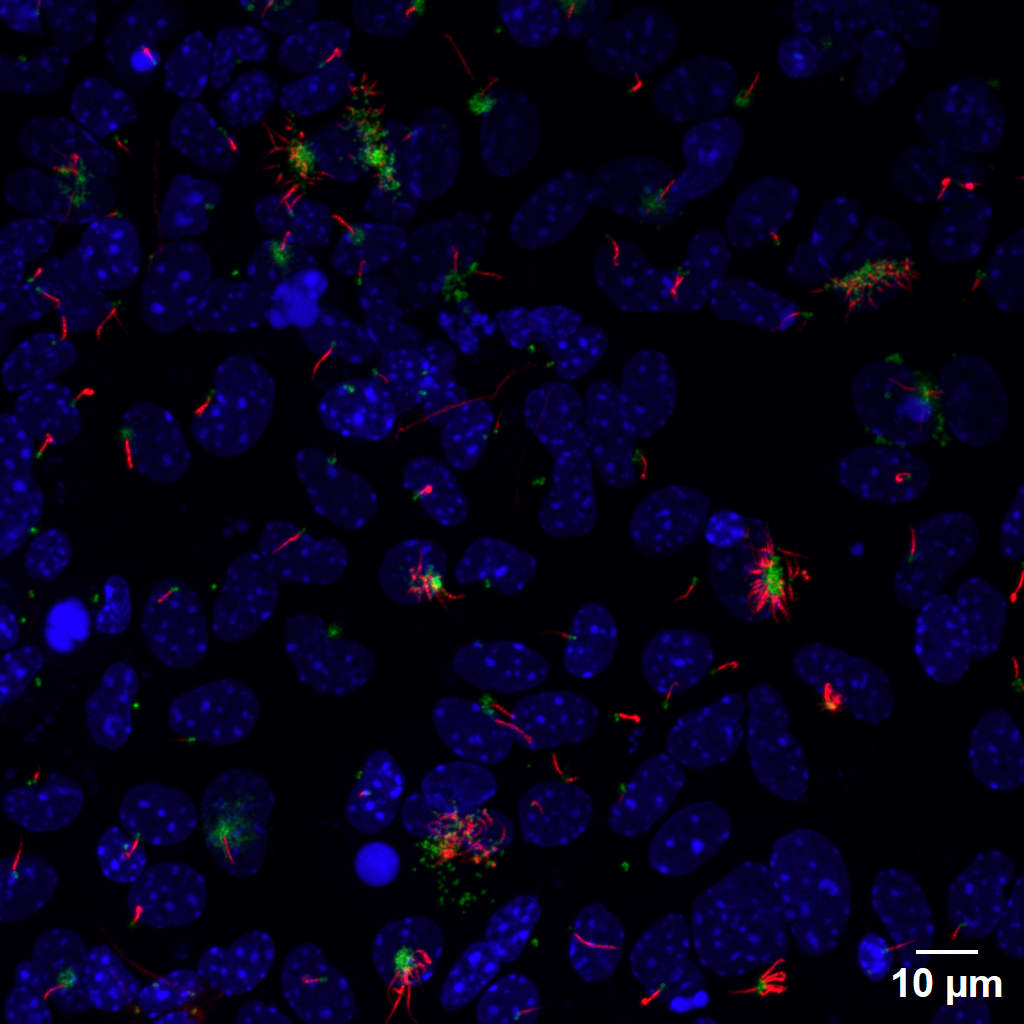

Supplement: Supplementary file 10 — Source data Fig. 6 [file 44319_2025_460_MOESM10_ESM.zip › Figure 6/D/EC Ad_GFP EtOH DAPI_Blue FOP_Green GT335_Red.tif]

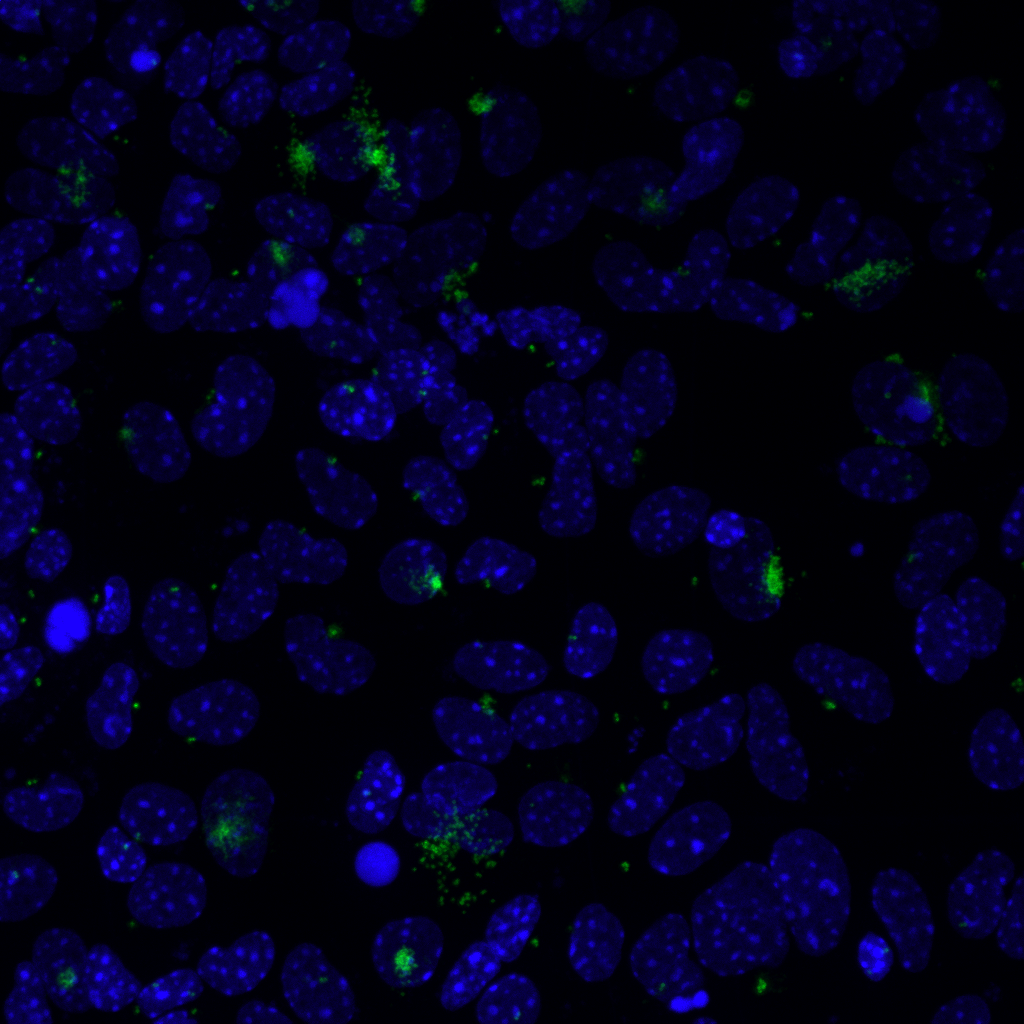

Supplement: Supplementary file 10 — Source data Fig. 6 [file 44319_2025_460_MOESM10_ESM.zip › Figure 6/D/EC Ad_GFP EtOH DAPI_Blue FOP_Green.tif]

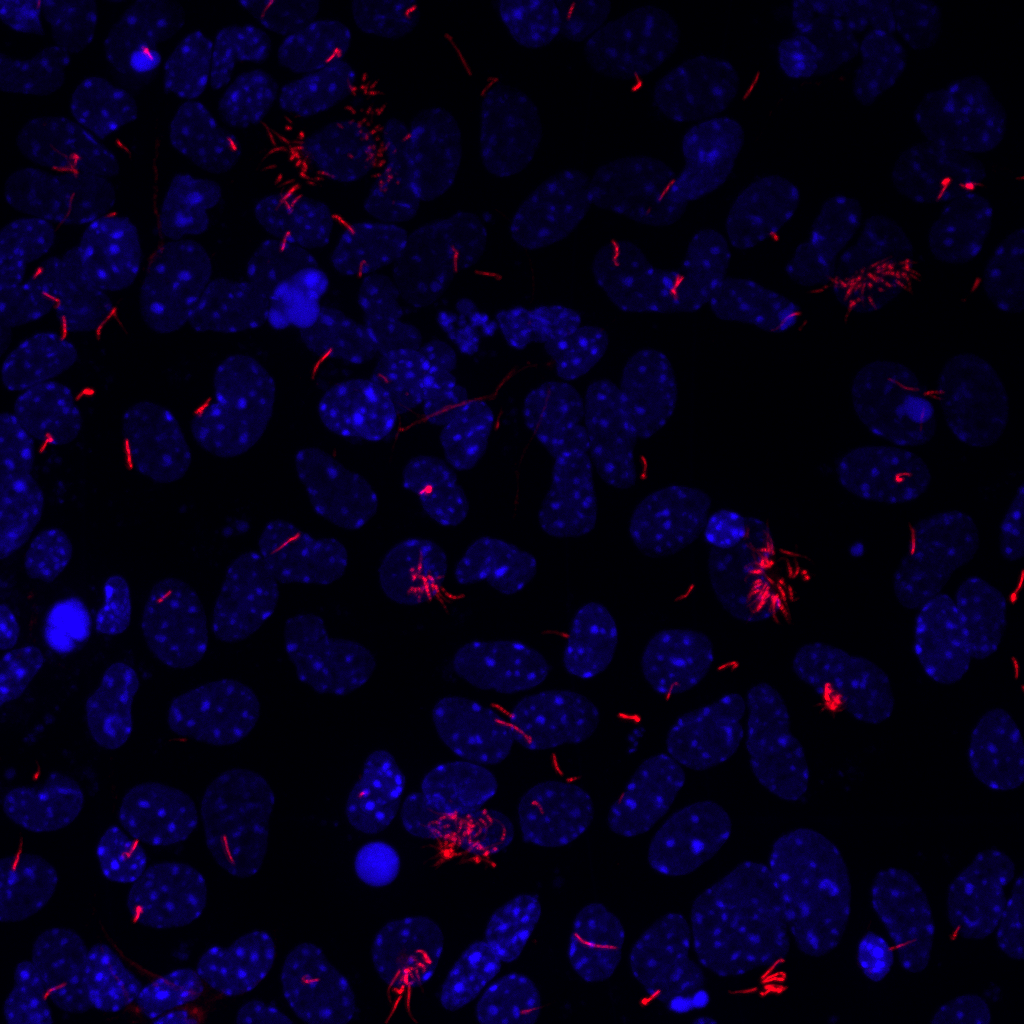

Supplement: Supplementary file 10 — Source data Fig. 6 [file 44319_2025_460_MOESM10_ESM.zip › Figure 6/D/EC Ad_GFP EtOH DAPI_Blue GT335_Red.tif]

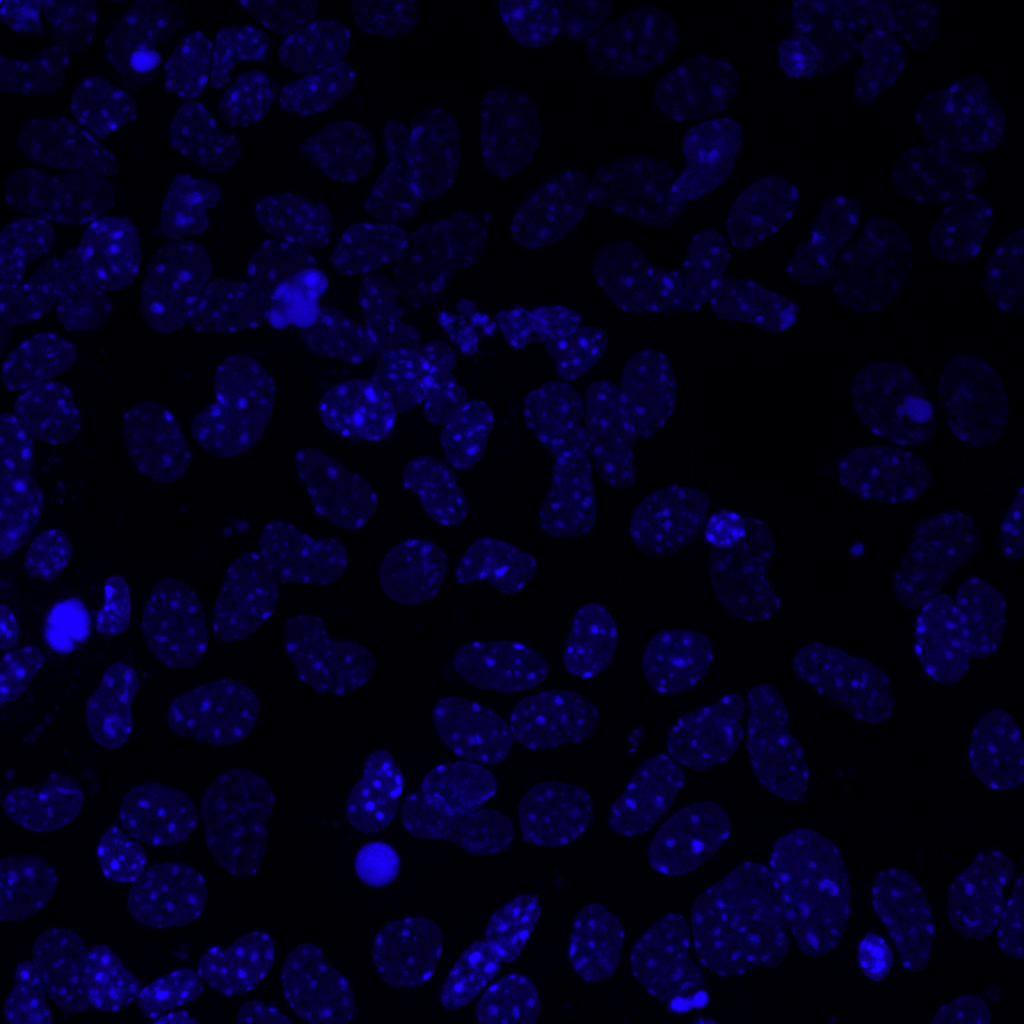

Supplement: Supplementary file 10 — Source data Fig. 6 [file 44319_2025_460_MOESM10_ESM.zip › Figure 6/D/EC Ad_GFP EtOH DAPI_Blue.tif]

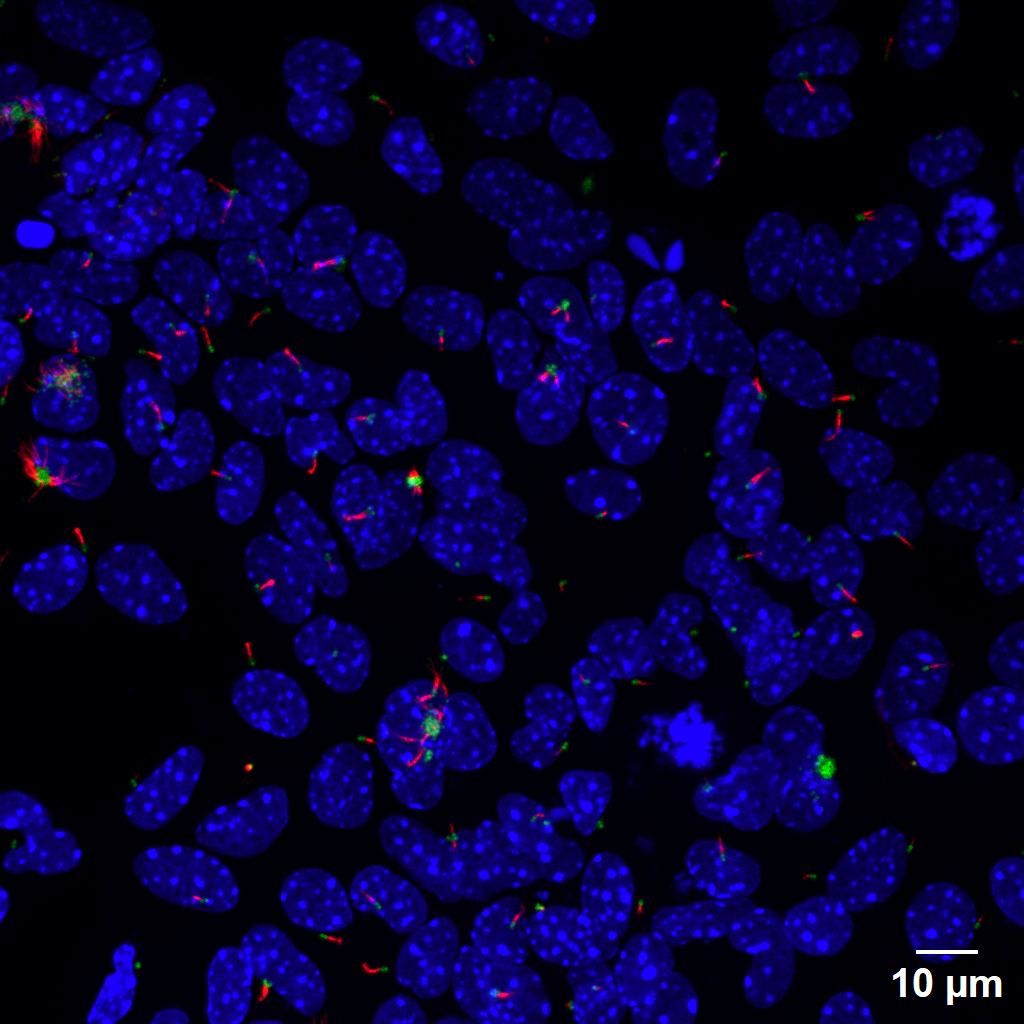

Supplement: Supplementary file 10 — Source data Fig. 6 [file 44319_2025_460_MOESM10_ESM.zip › Figure 6/D/EC Ad_GFP Rapa DAPI_Blue FOP_Green GT335_Red.tif]

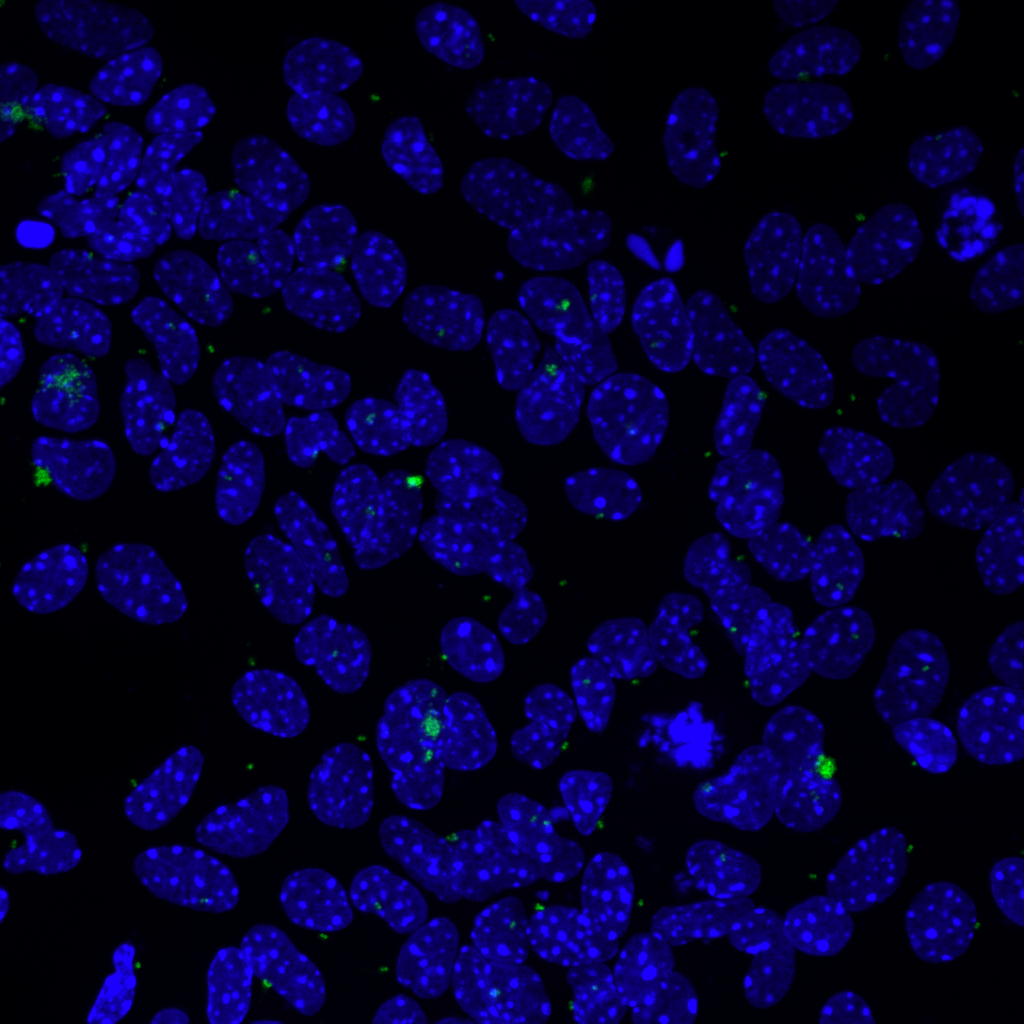

Supplement: Supplementary file 10 — Source data Fig. 6 [file 44319_2025_460_MOESM10_ESM.zip › Figure 6/D/EC Ad_GFP Rapa DAPI_Blue FOP_Green.tif]

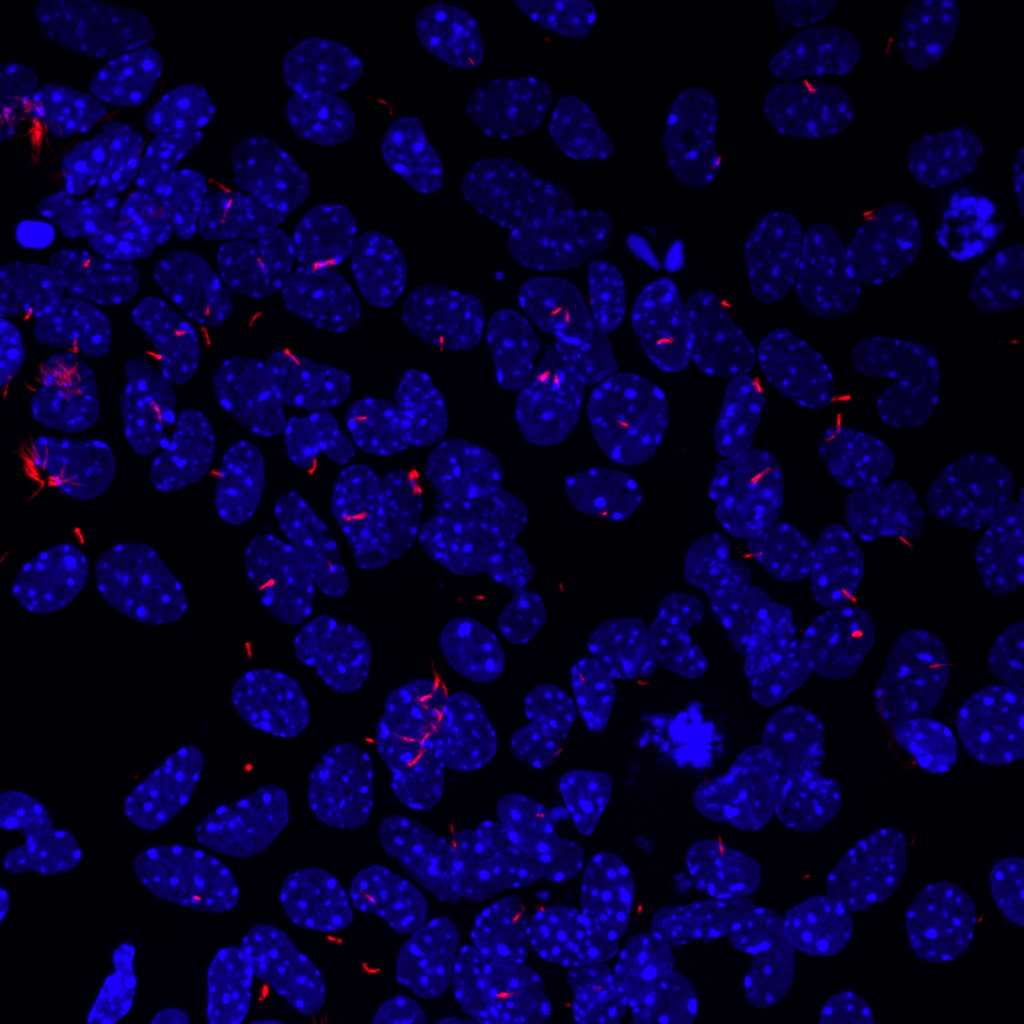

Supplement: Supplementary file 10 — Source data Fig. 6 [file 44319_2025_460_MOESM10_ESM.zip › Figure 6/D/EC Ad_GFP Rapa DAPI_Blue GT335_Red.tif]

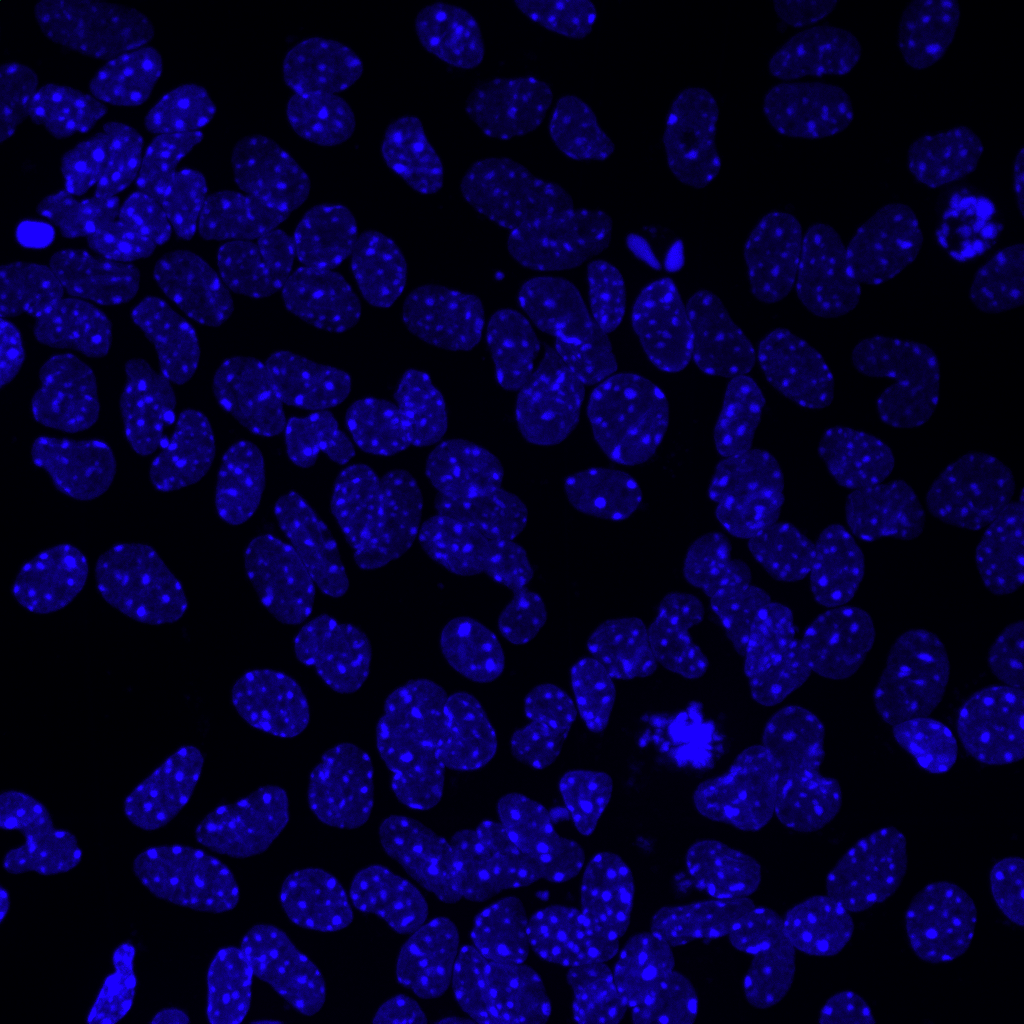

Supplement: Supplementary file 10 — Source data Fig. 6 [file 44319_2025_460_MOESM10_ESM.zip › Figure 6/D/EC Ad_GFP Rapa DAPI_Blue.tif]

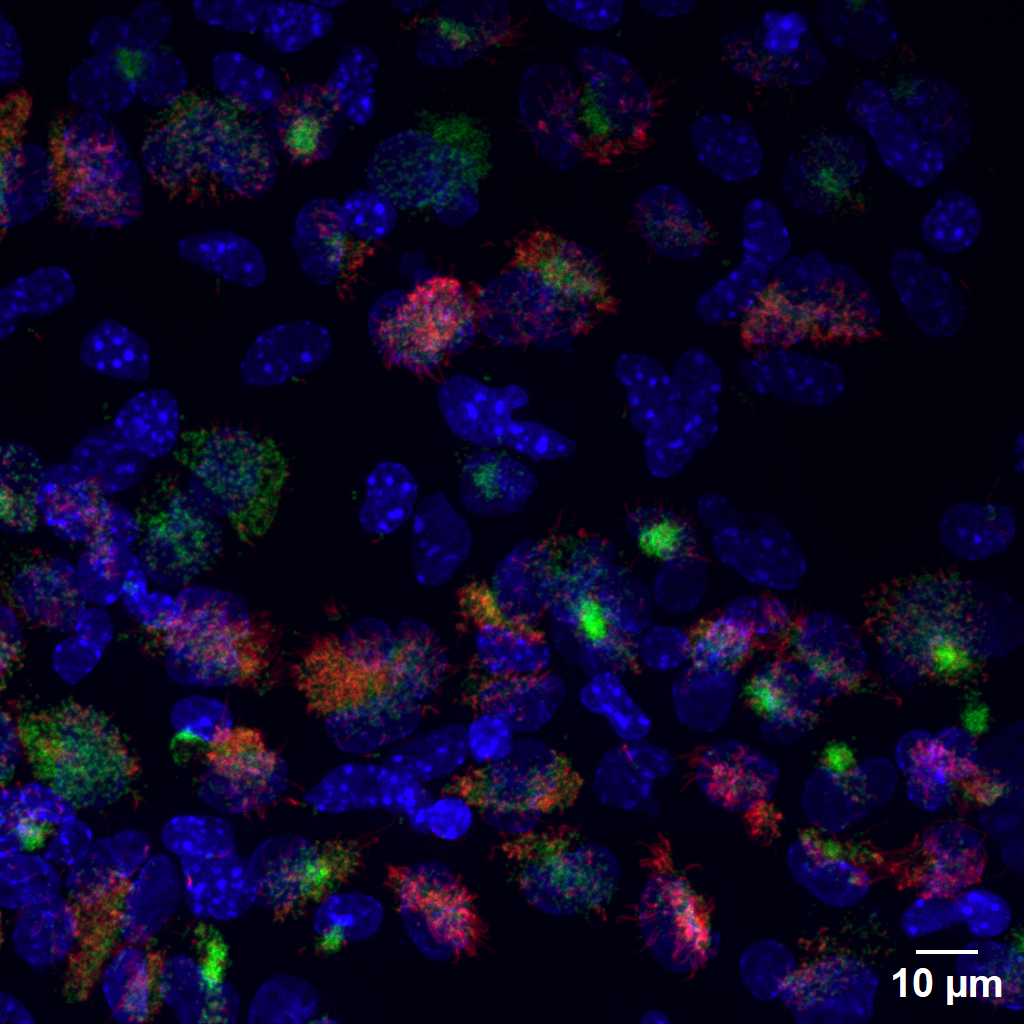

Supplement: Supplementary file 10 — Source data Fig. 6 [file 44319_2025_460_MOESM10_ESM.zip › Figure 6/D/EC Ad_MCIDAS_E2F4 EtOH DAPI_Blue FOP_Green GT335_Red.tif]

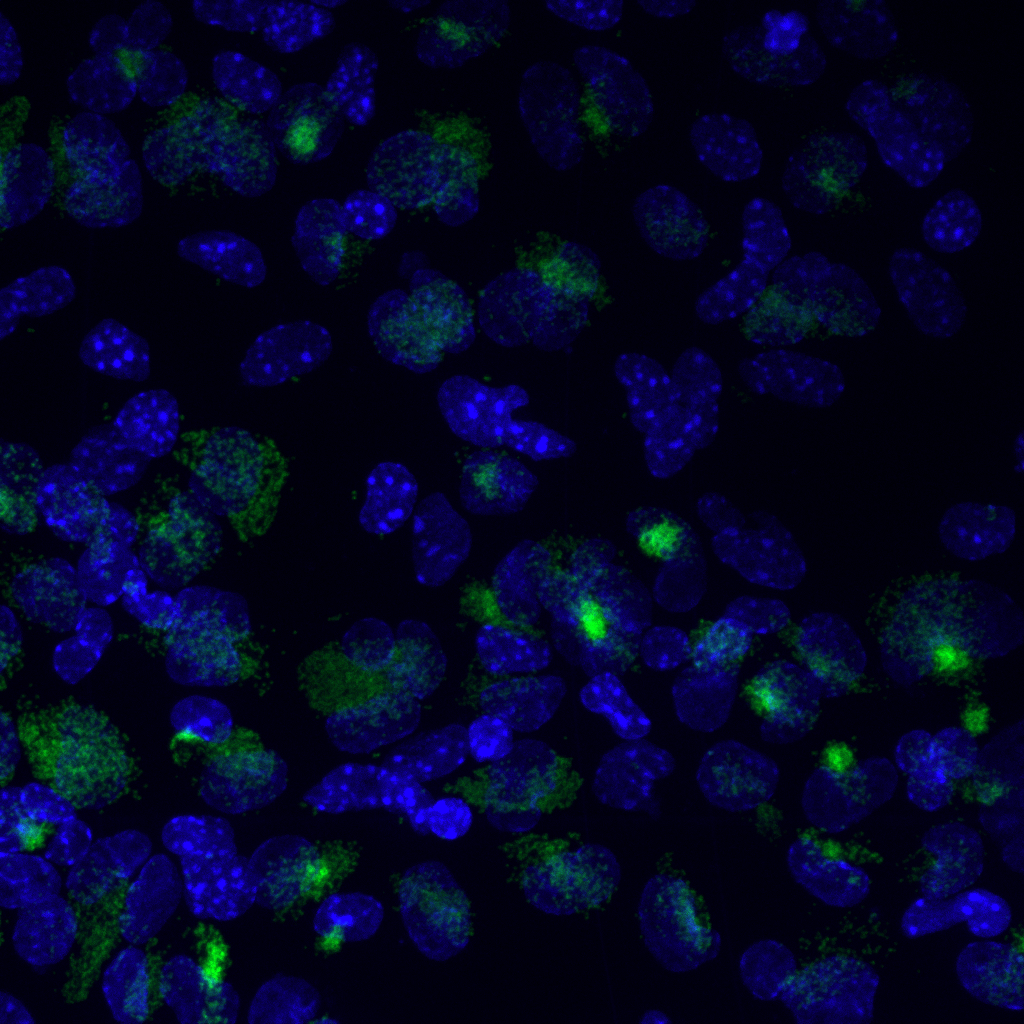

Supplement: Supplementary file 10 — Source data Fig. 6 [file 44319_2025_460_MOESM10_ESM.zip › Figure 6/D/EC Ad_MCIDAS_E2F4 EtOH DAPI_Blue FOP_Green.tif]

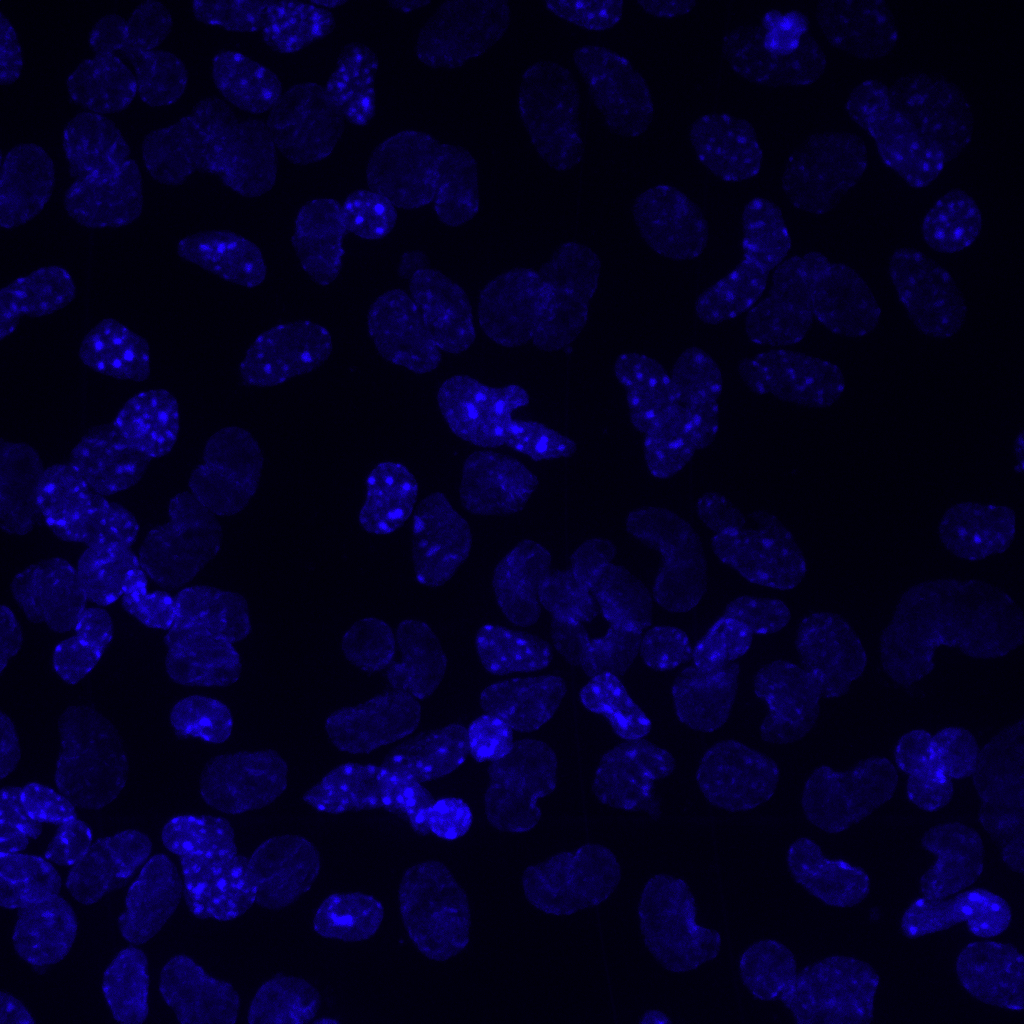

Supplement: Supplementary file 10 — Source data Fig. 6 [file 44319_2025_460_MOESM10_ESM.zip › Figure 6/D/EC Ad_MCIDAS_E2F4 EtOH DAPI_Blue.tif]

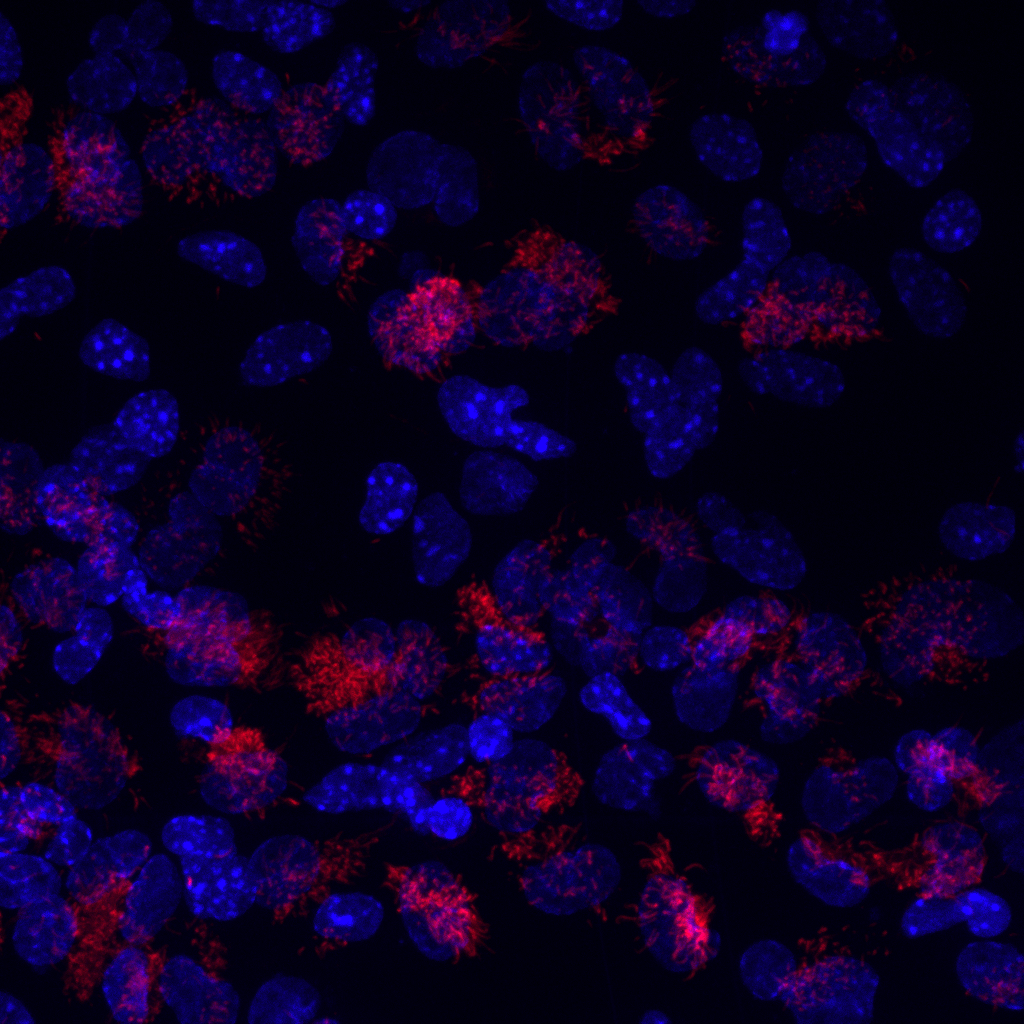

Supplement: Supplementary file 10 — Source data Fig. 6 [file 44319_2025_460_MOESM10_ESM.zip › Figure 6/D/EC Ad_MCIDAS_E2F4 EtOH DAPI_BlueGT335_Red.tif]

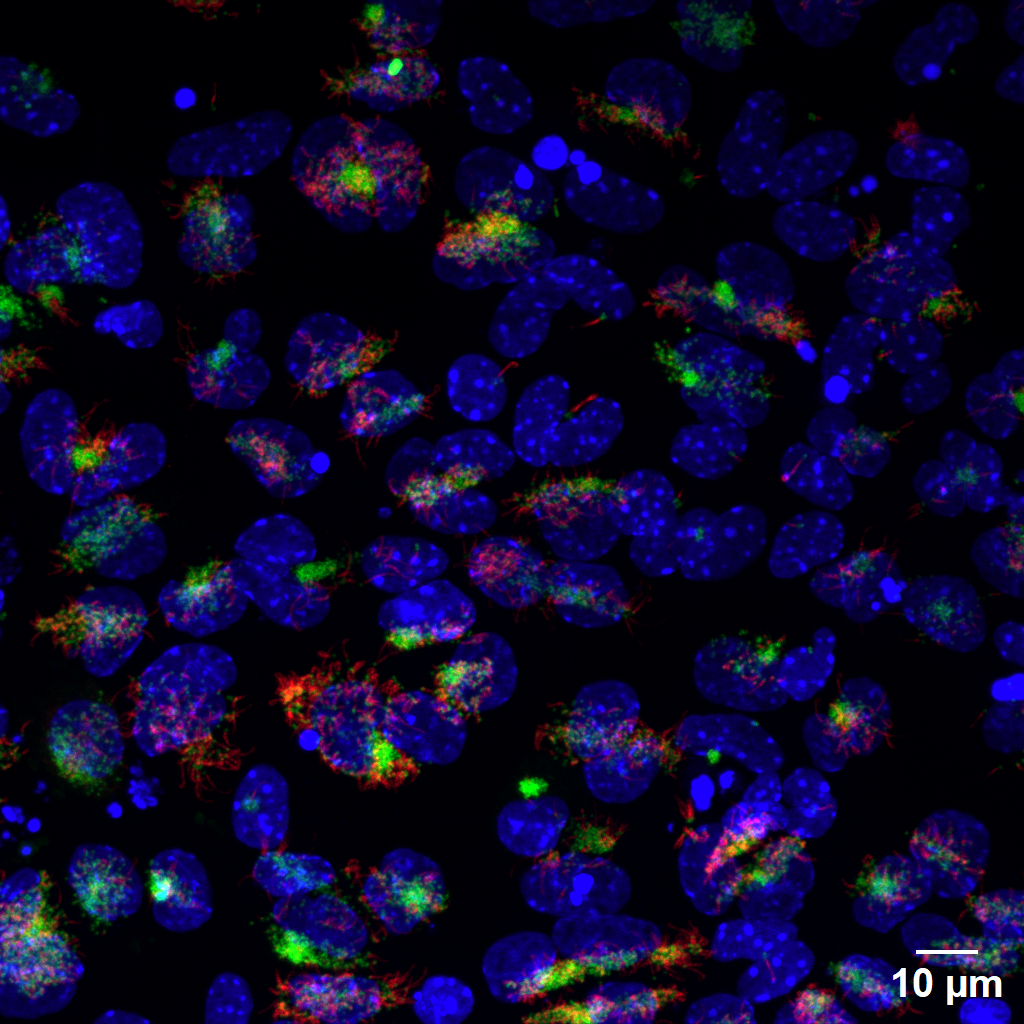

Supplement: Supplementary file 10 — Source data Fig. 6 [file 44319_2025_460_MOESM10_ESM.zip › Figure 6/D/EC Ad_MCIDAS_E2F4 Rapa DAPI_Blue FOP_Green GT335_Red.tif]

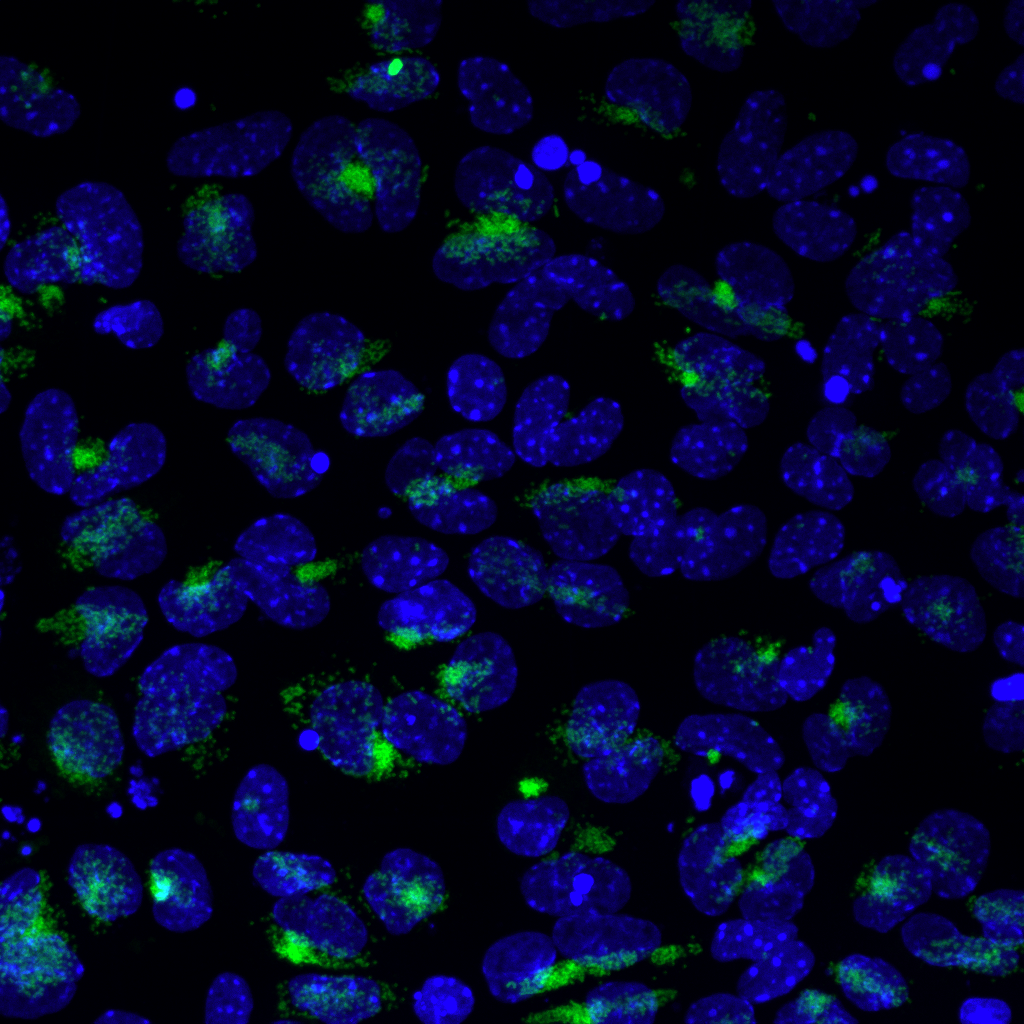

Supplement: Supplementary file 10 — Source data Fig. 6 [file 44319_2025_460_MOESM10_ESM.zip › Figure 6/D/EC Ad_MCIDAS_E2F4 Rapa DAPI_Blue FOP_Green.tif]

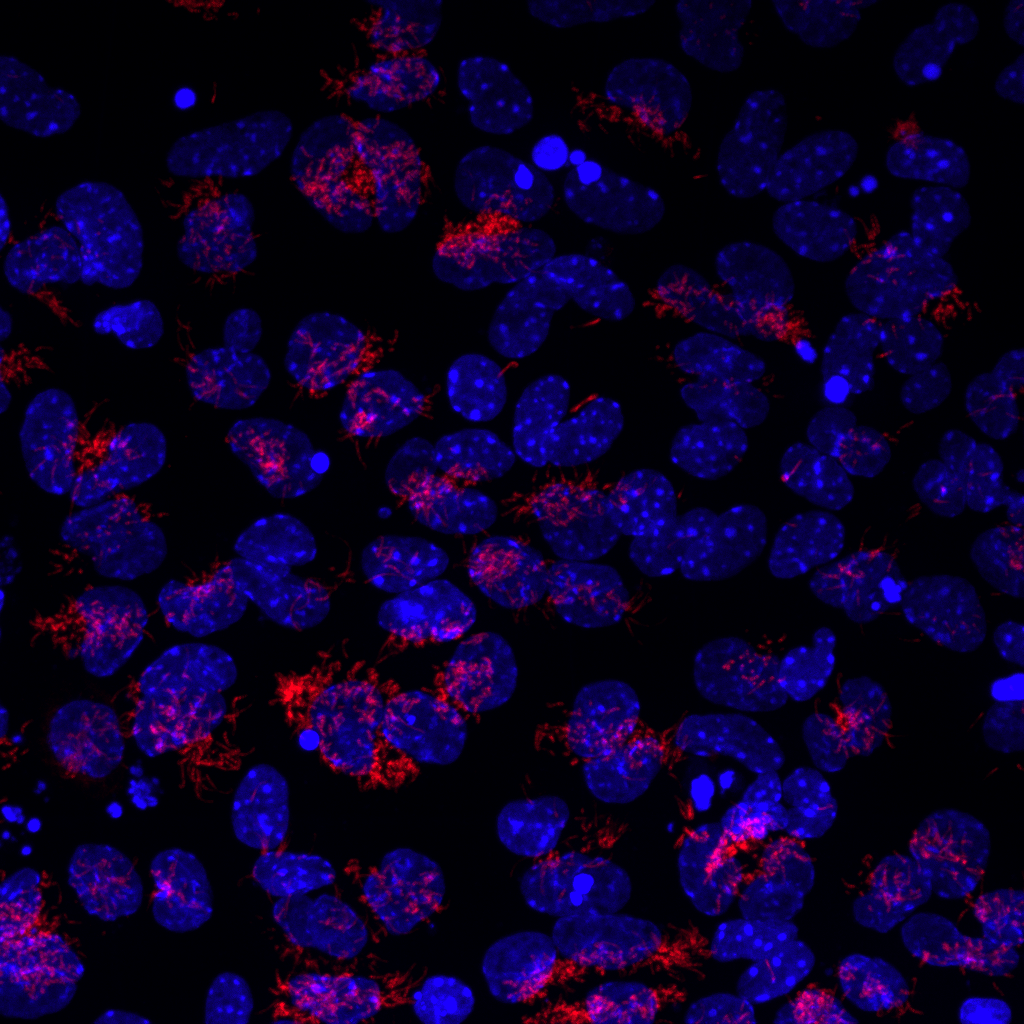

Supplement: Supplementary file 10 — Source data Fig. 6 [file 44319_2025_460_MOESM10_ESM.zip › Figure 6/D/EC Ad_MCIDAS_E2F4 Rapa DAPI_Blue GT335_Red.tif]

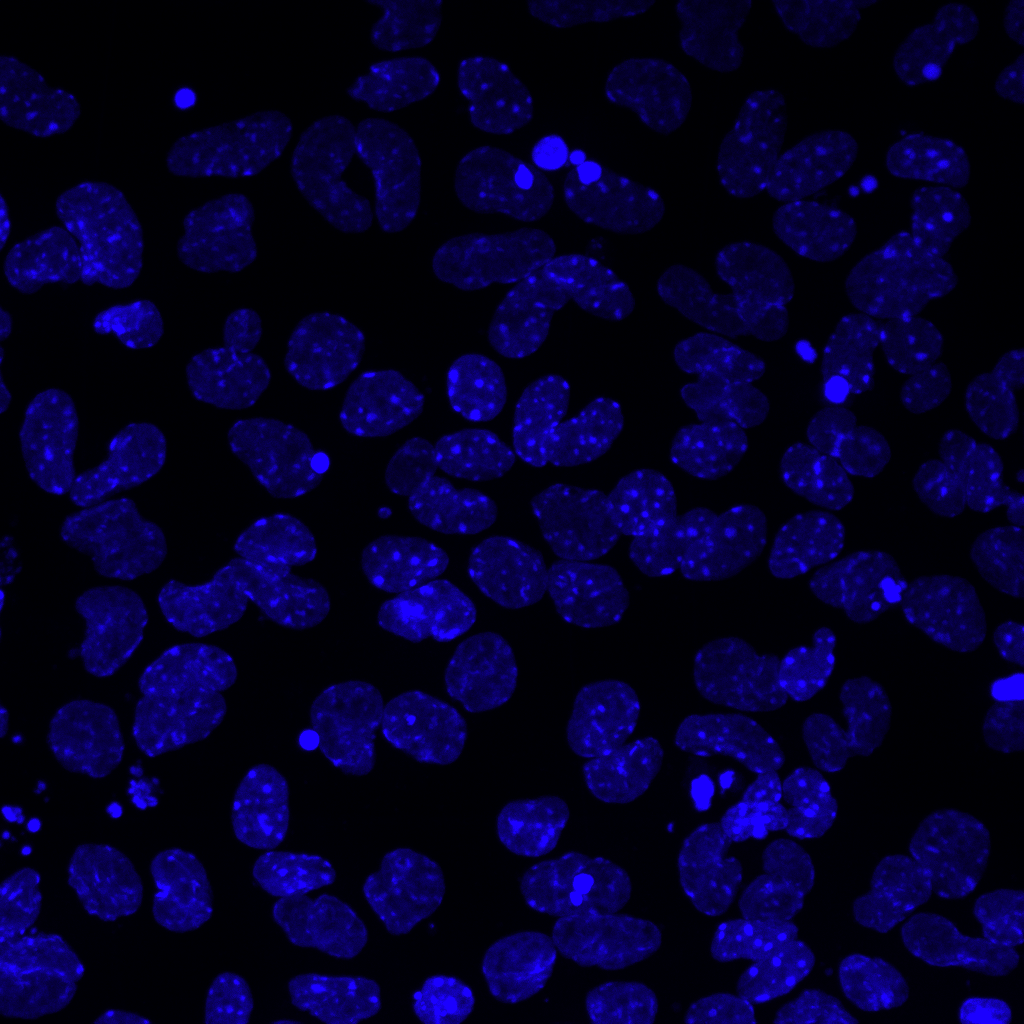

Supplement: Supplementary file 10 — Source data Fig. 6 [file 44319_2025_460_MOESM10_ESM.zip › Figure 6/D/EC Ad_MCIDAS_E2F4 Rapa DAPI_Blue.tif]

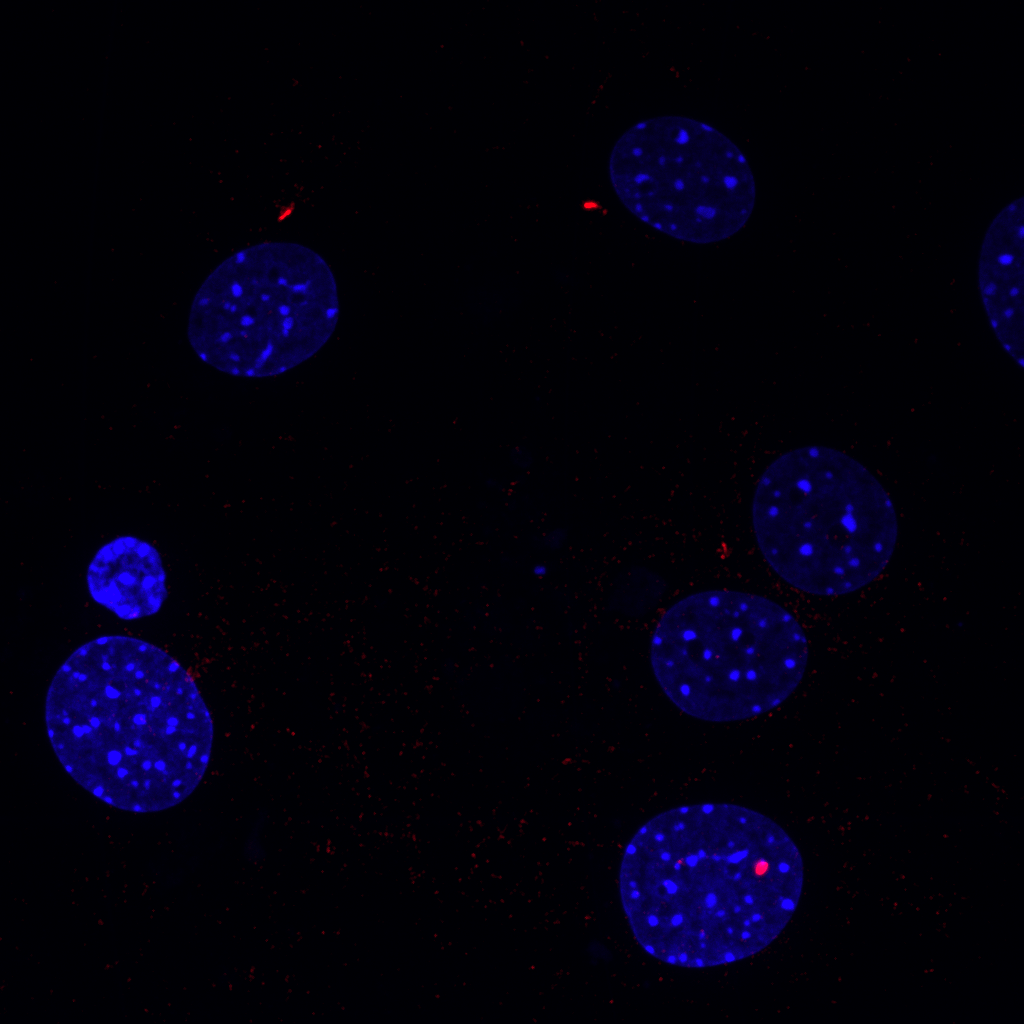

Supplement: Supplementary file 10 — Source data Fig. 6 [file 44319_2025_460_MOESM10_ESM.zip › Figure 6/F/MEF Dif5 EtOH Ad_GFP DAPI_Blue GT335_Red.tif]

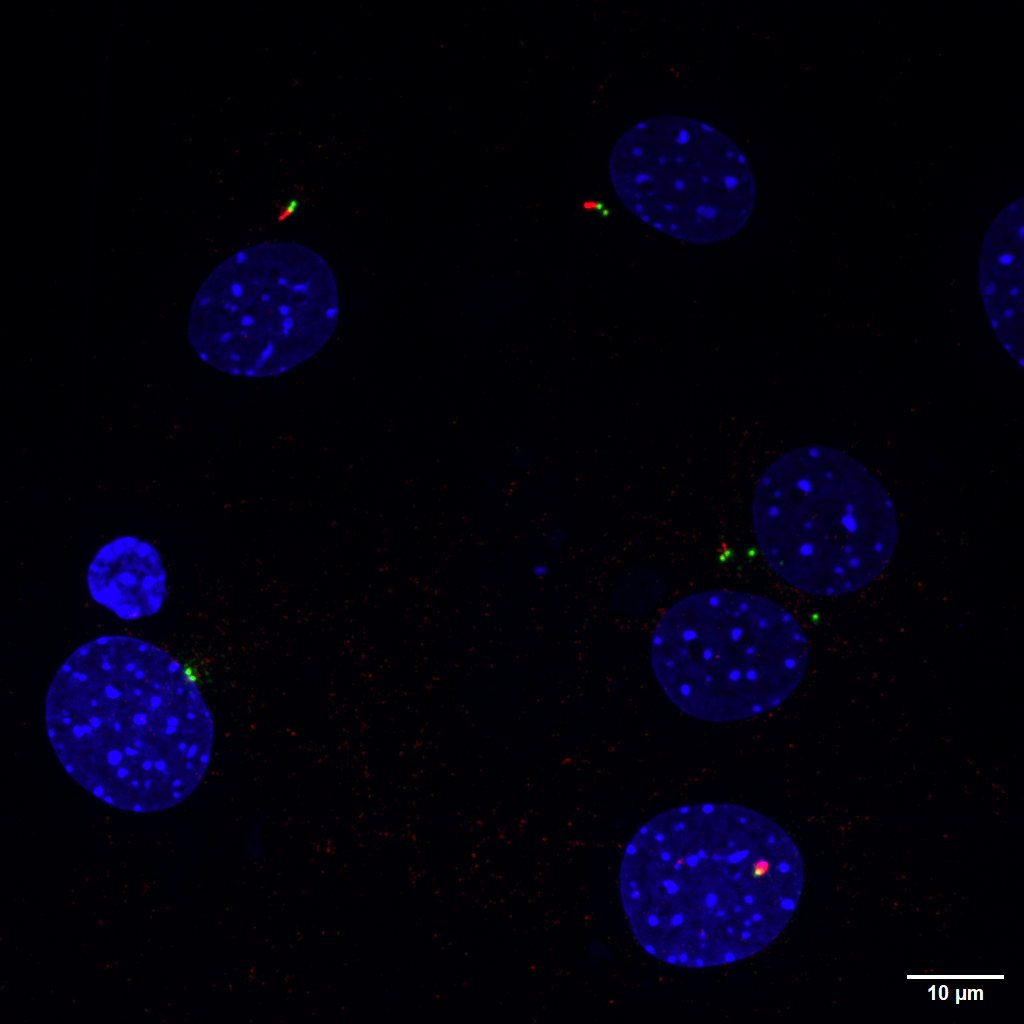

Supplement: Supplementary file 10 — Source data Fig. 6 [file 44319_2025_460_MOESM10_ESM.zip › Figure 6/F/MEF Dif5 EtOH Ad_GFP DAPI_Blue FOP_Green GT335_Red.tif]

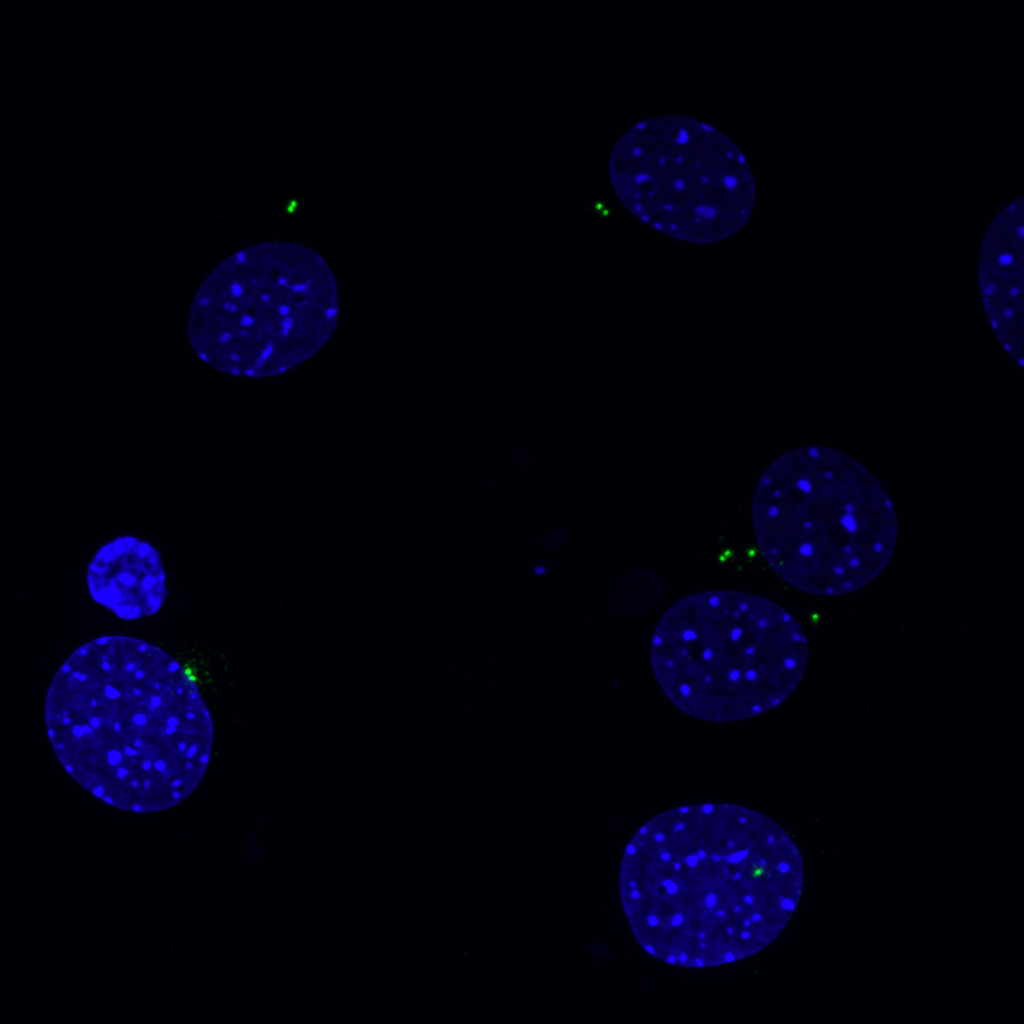

Supplement: Supplementary file 10 — Source data Fig. 6 [file 44319_2025_460_MOESM10_ESM.zip › Figure 6/F/MEF Dif5 EtOH Ad_GFP DAPI_Blue FOP_Green.tif]

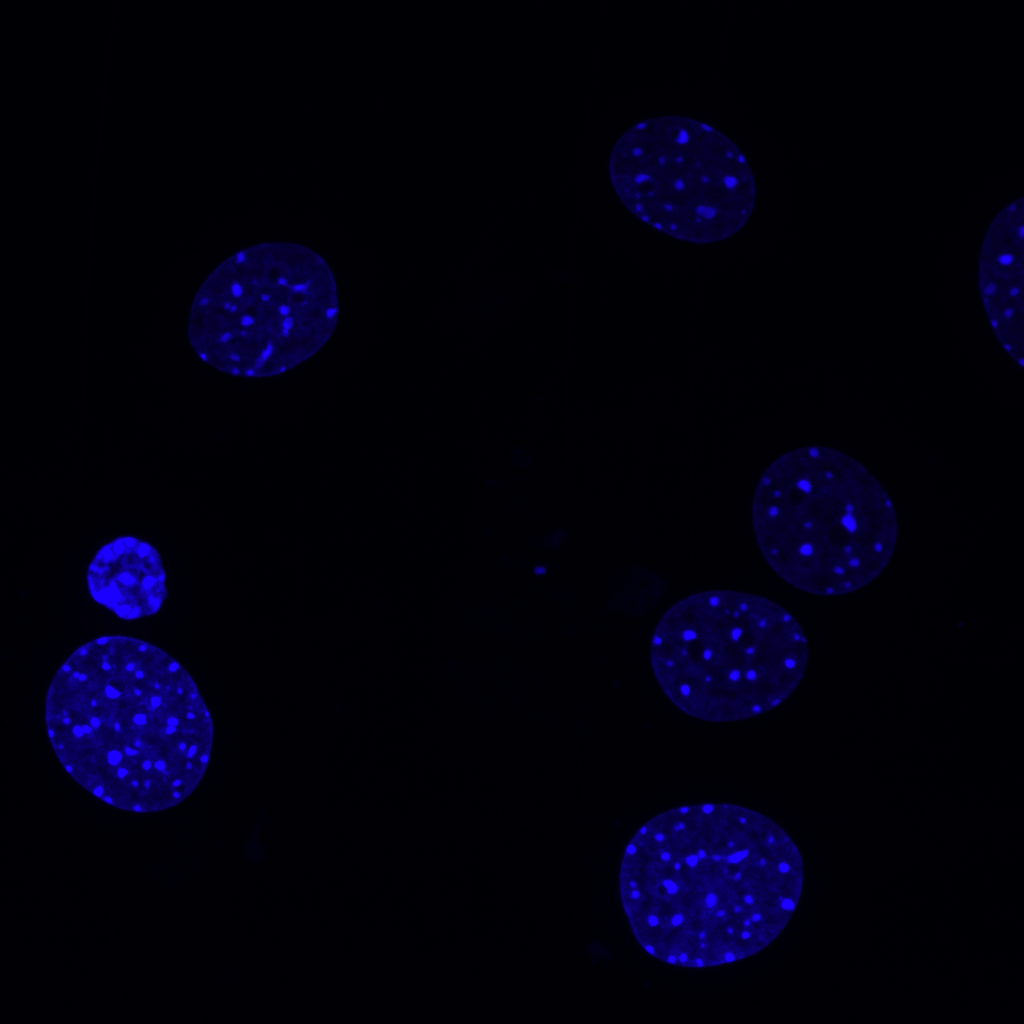

Supplement: Supplementary file 10 — Source data Fig. 6 [file 44319_2025_460_MOESM10_ESM.zip › Figure 6/F/MEF Dif5 EtOH Ad_GFP DAPI_Blue.tif]

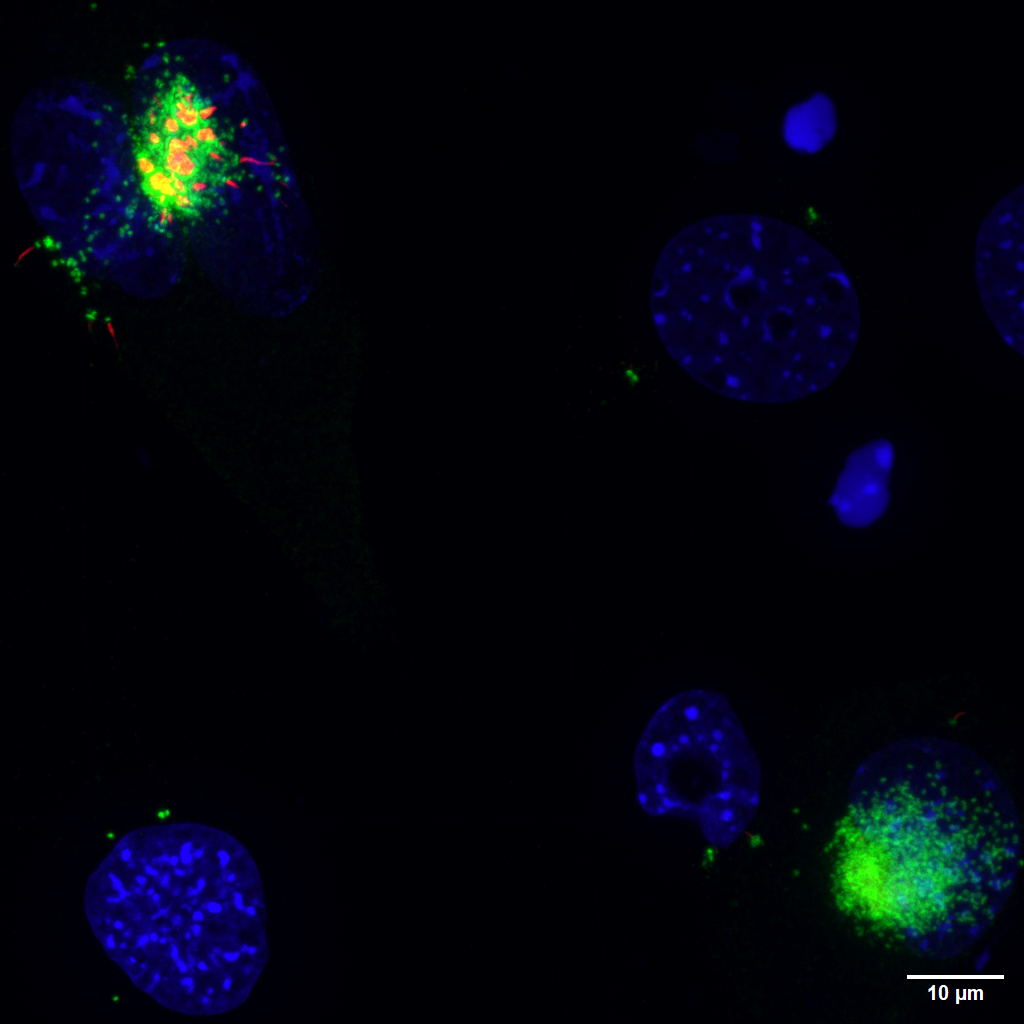

Supplement: Supplementary file 10 — Source data Fig. 6 [file 44319_2025_460_MOESM10_ESM.zip › Figure 6/F/MEF Dif5 EtOH Ad_MCIDAS_E2F4 DAPI_Blue FOP_Green GT335_Red.tif]

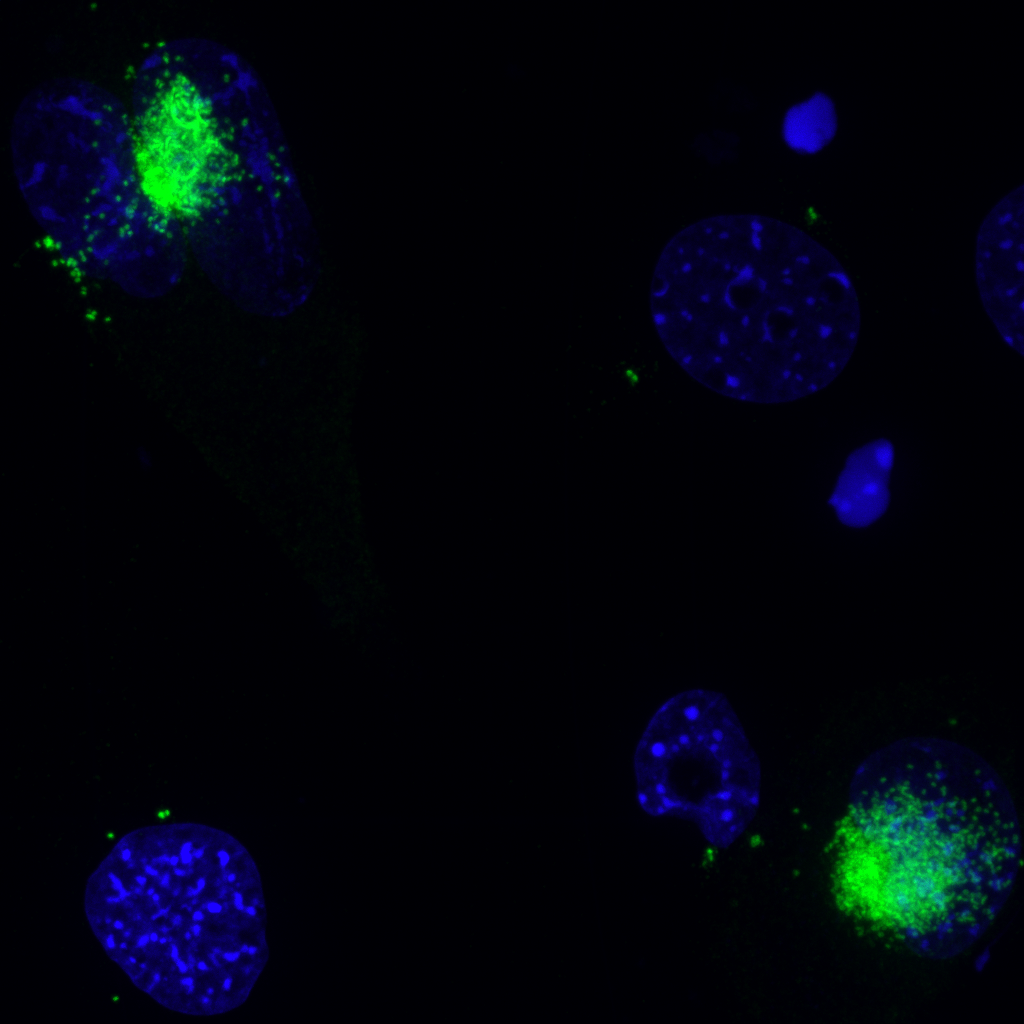

Supplement: Supplementary file 10 — Source data Fig. 6 [file 44319_2025_460_MOESM10_ESM.zip › Figure 6/F/MEF Dif5 EtOH Ad_MCIDAS_E2F4 DAPI_Blue FOP_Green.tif]

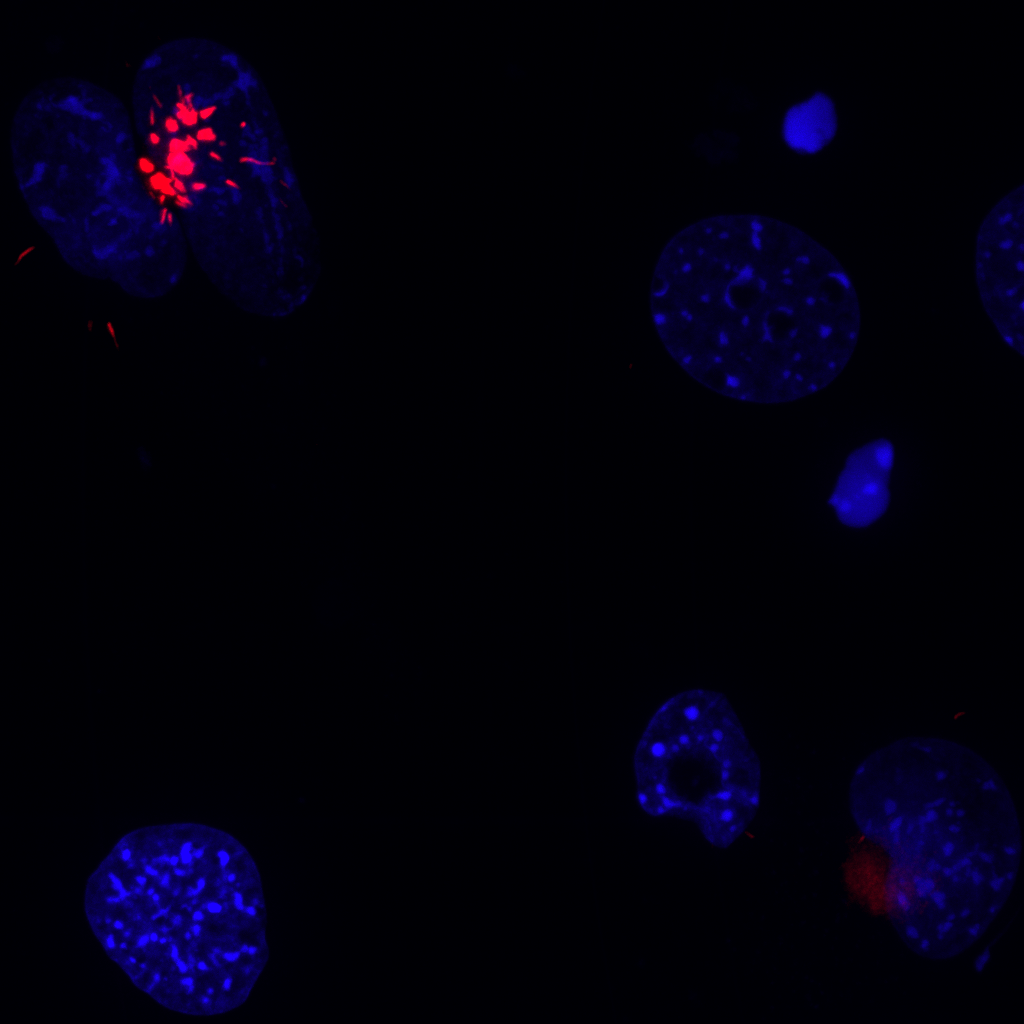

Supplement: Supplementary file 10 — Source data Fig. 6 [file 44319_2025_460_MOESM10_ESM.zip › Figure 6/F/MEF Dif5 EtOH Ad_MCIDAS_E2F4 DAPI_Blue GT335_Red.tif]

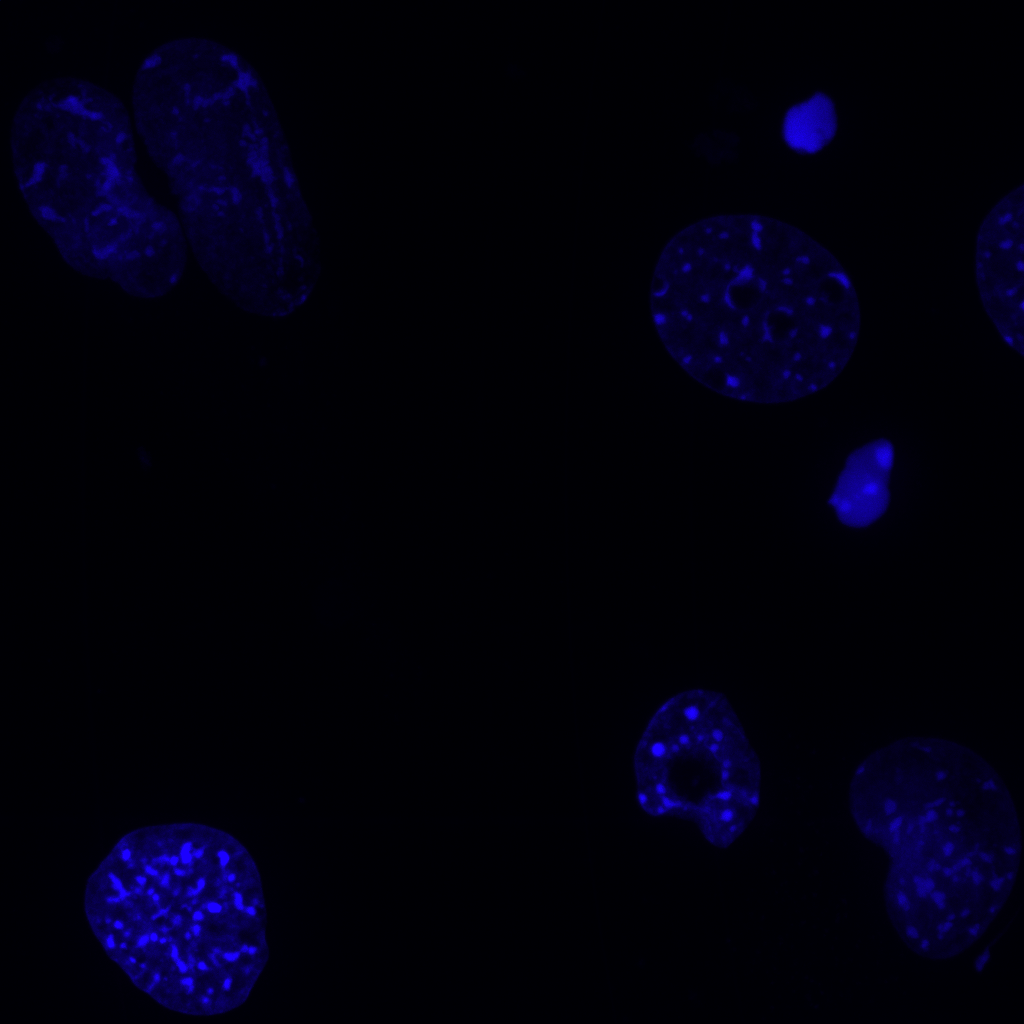

Supplement: Supplementary file 10 — Source data Fig. 6 [file 44319_2025_460_MOESM10_ESM.zip › Figure 6/F/MEF Dif5 EtOH Ad_MCIDAS_E2F4 DAPI_Blue.tif]

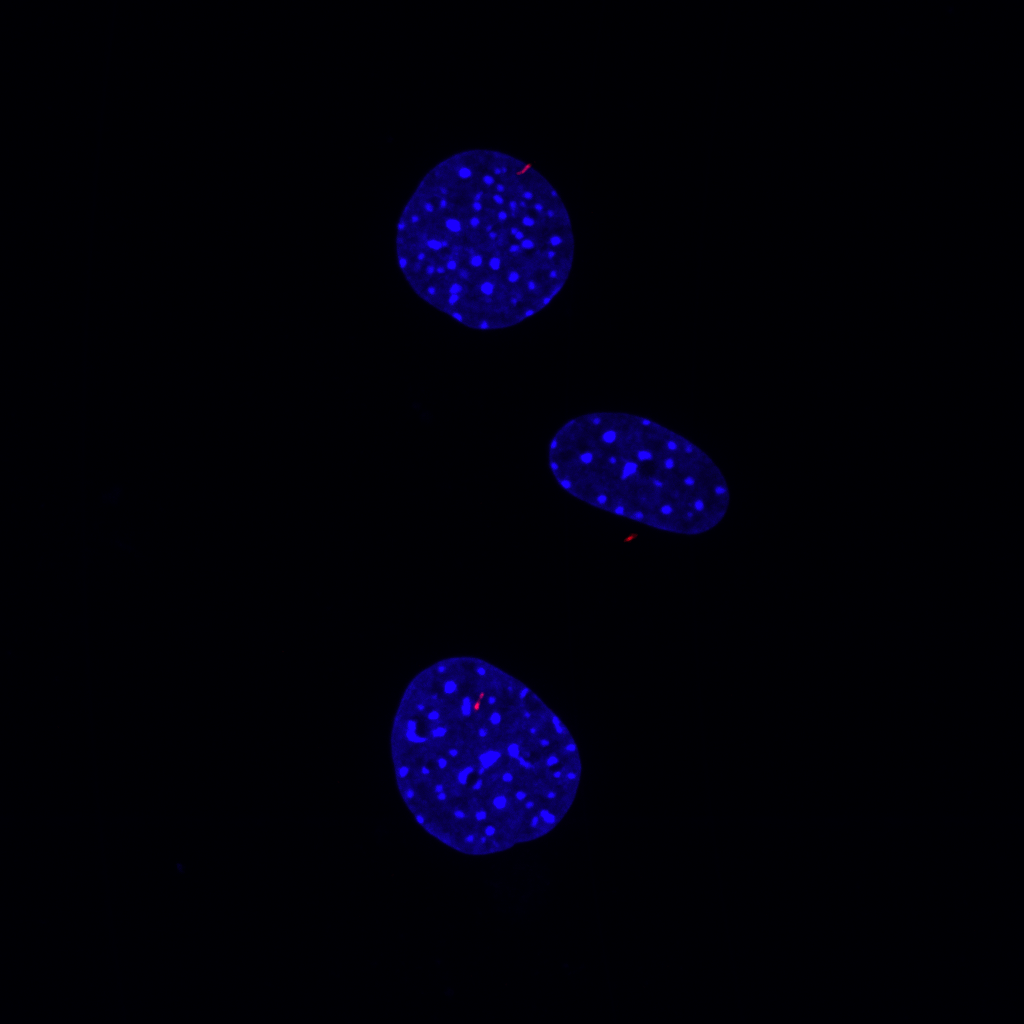

Supplement: Supplementary file 10 — Source data Fig. 6 [file 44319_2025_460_MOESM10_ESM.zip › Figure 6/F/MEF Dif5 Rapa Ad_GFP DAPI_Blue GT335_Red.tif]

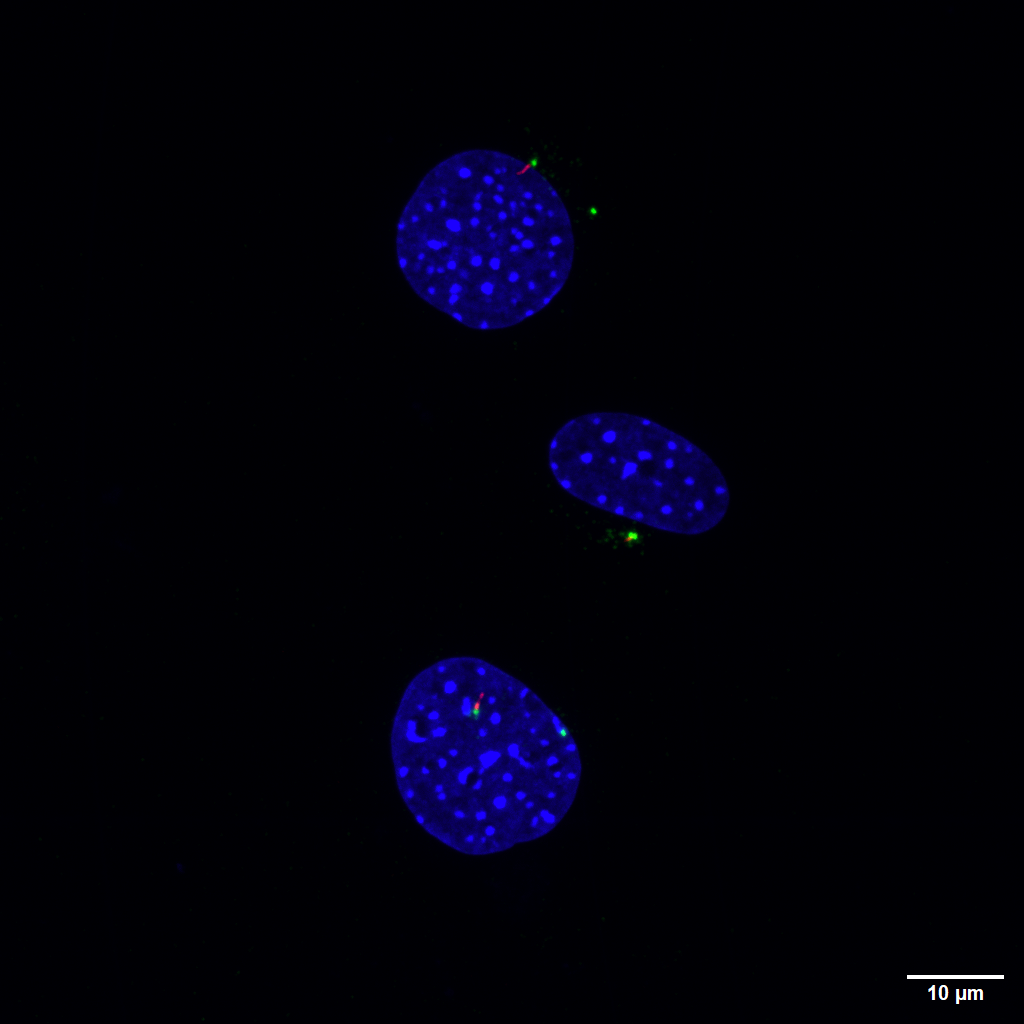

Supplement: Supplementary file 10 — Source data Fig. 6 [file 44319_2025_460_MOESM10_ESM.zip › Figure 6/F/MEF Dif5 Rapa Ad_GFP DAPI_Blue FOP_Green GT335_Red.tif]

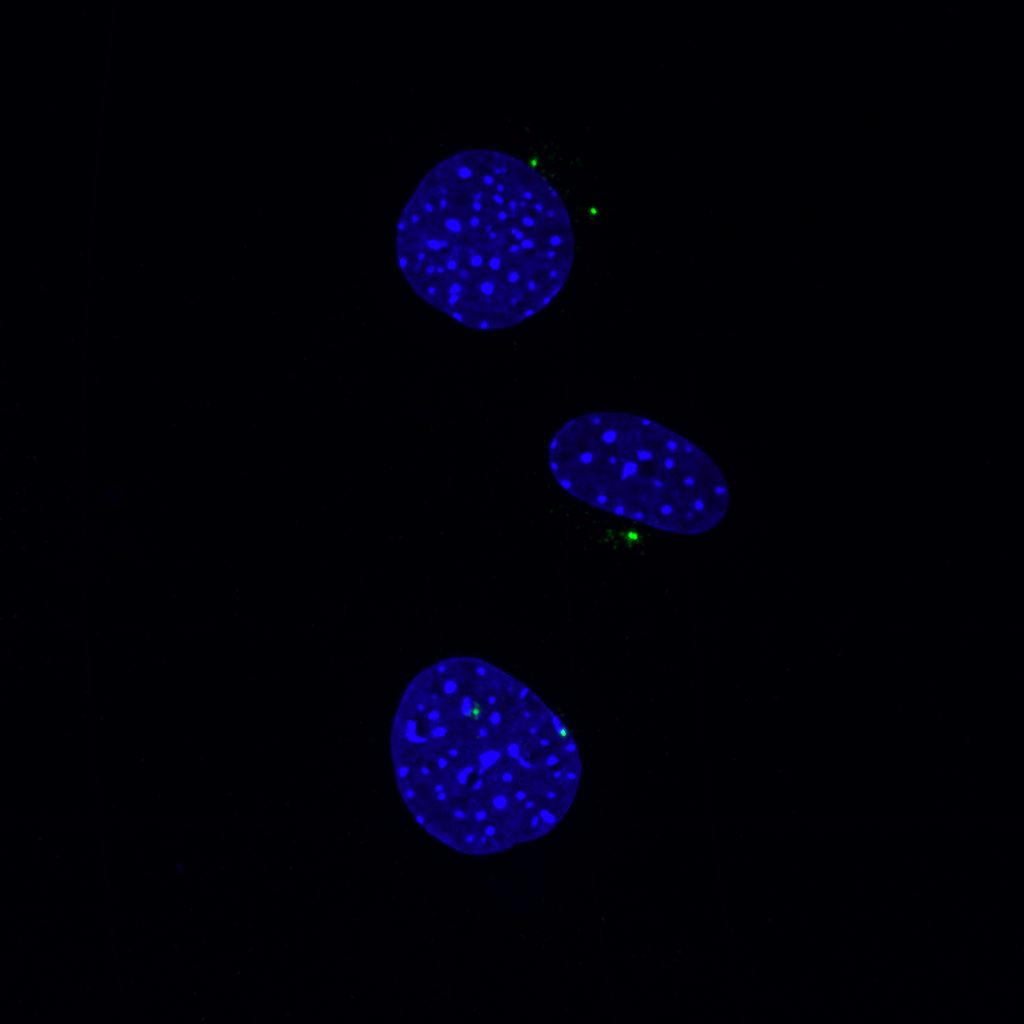

Supplement: Supplementary file 10 — Source data Fig. 6 [file 44319_2025_460_MOESM10_ESM.zip › Figure 6/F/MEF Dif5 Rapa Ad_GFP DAPI_Blue FOP_Green.tif]

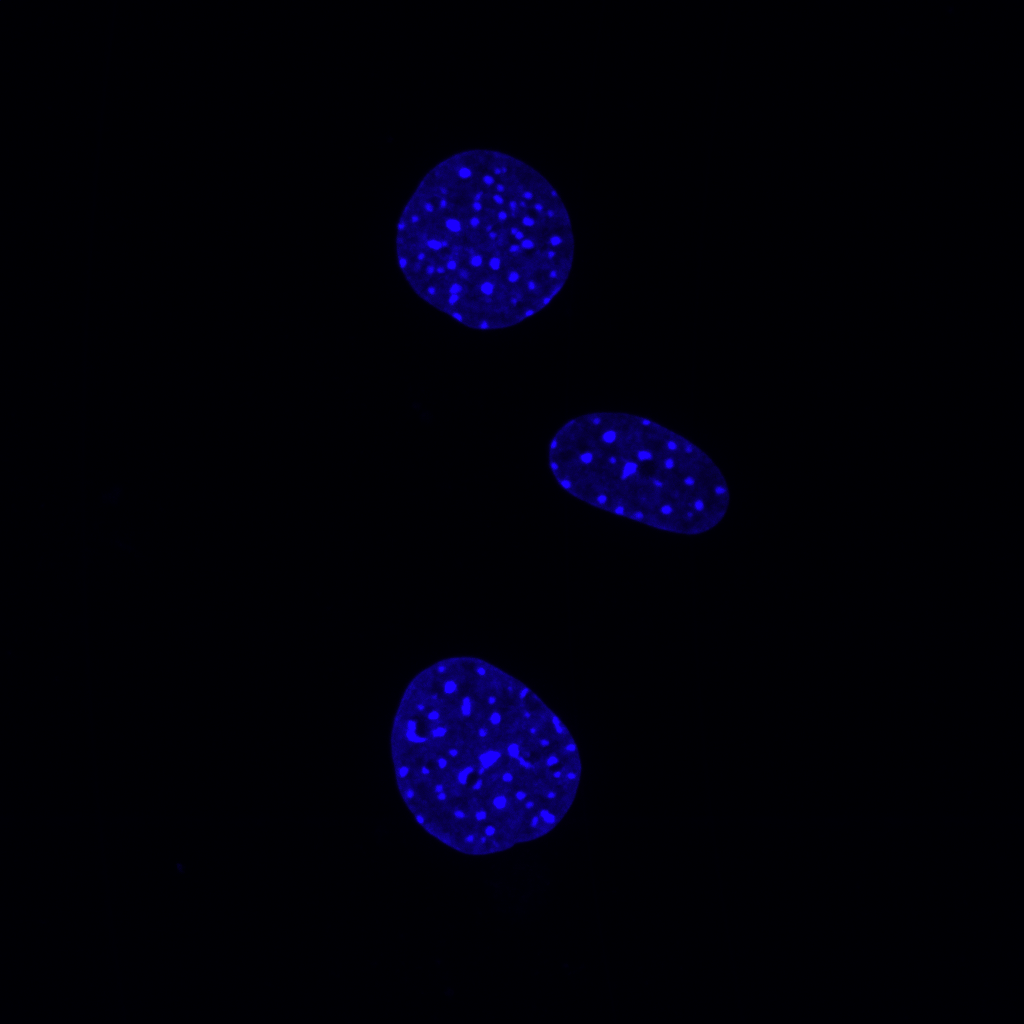

Supplement: Supplementary file 10 — Source data Fig. 6 [file 44319_2025_460_MOESM10_ESM.zip › Figure 6/F/MEF Dif5 Rapa Ad_GFP DAPI_Blue.tif]

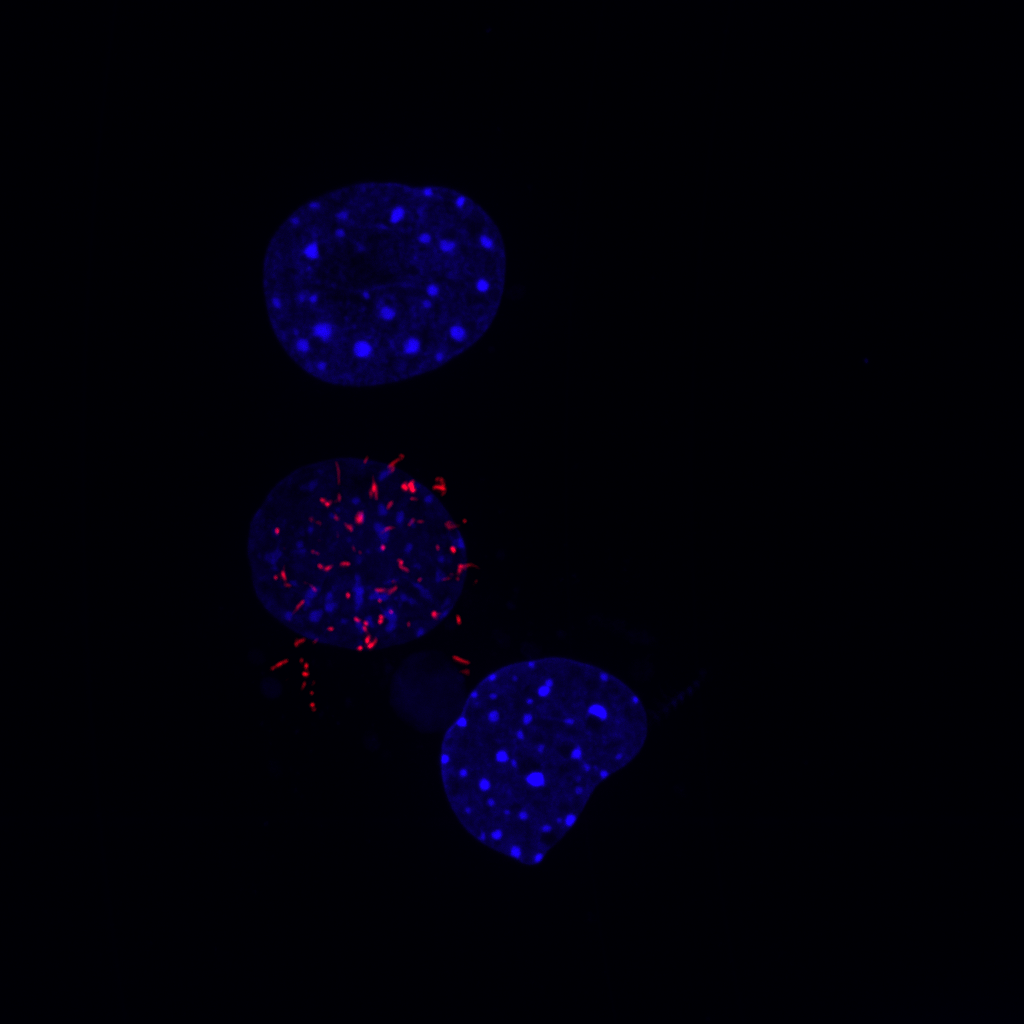

Supplement: Supplementary file 10 — Source data Fig. 6 [file 44319_2025_460_MOESM10_ESM.zip › Figure 6/F/MEF Dif5 Rapa Ad_MCIDAS_E2F4 DAPI_Blue GT335_Red.tif]

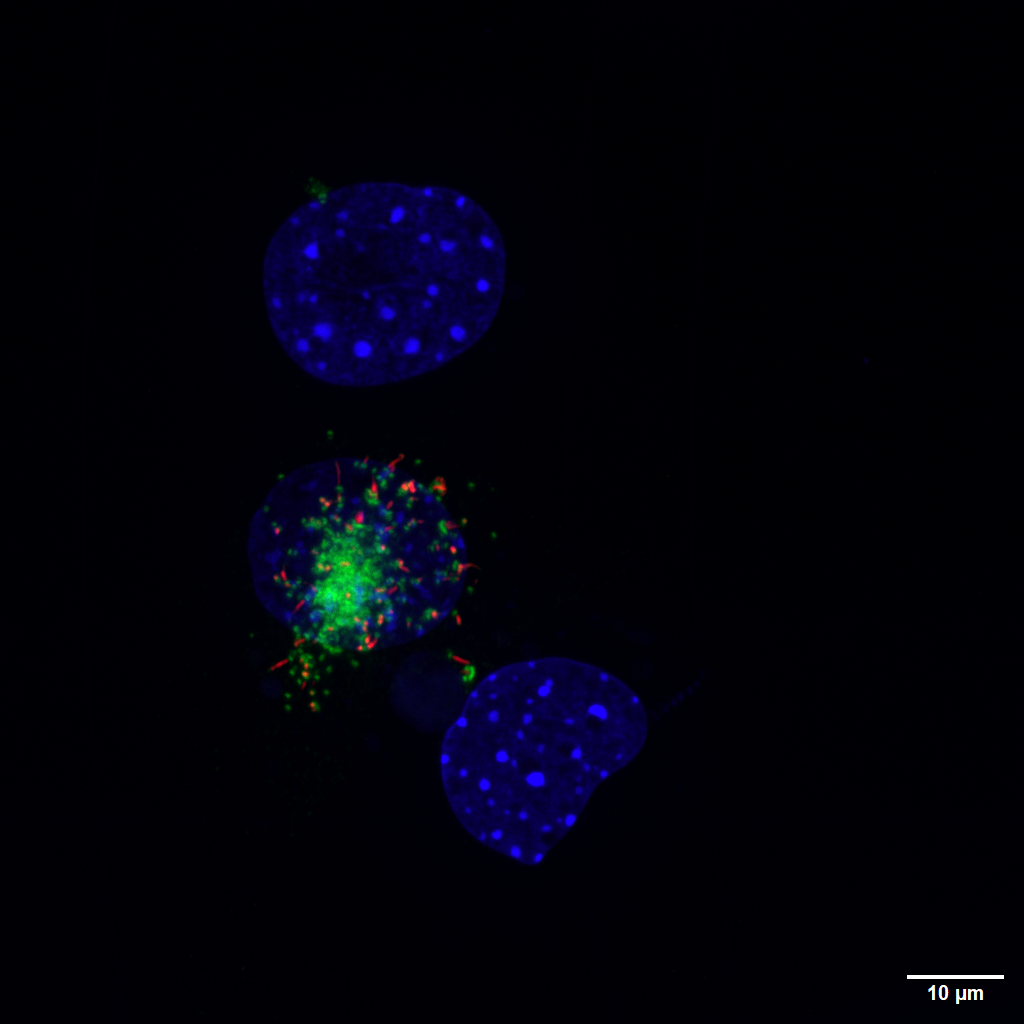

Supplement: Supplementary file 10 — Source data Fig. 6 [file 44319_2025_460_MOESM10_ESM.zip › Figure 6/F/MEF Dif5 Rapa Ad_MCIDAS_E2F4 DAPI_Blue FOP_Green GT335_Red.tif]

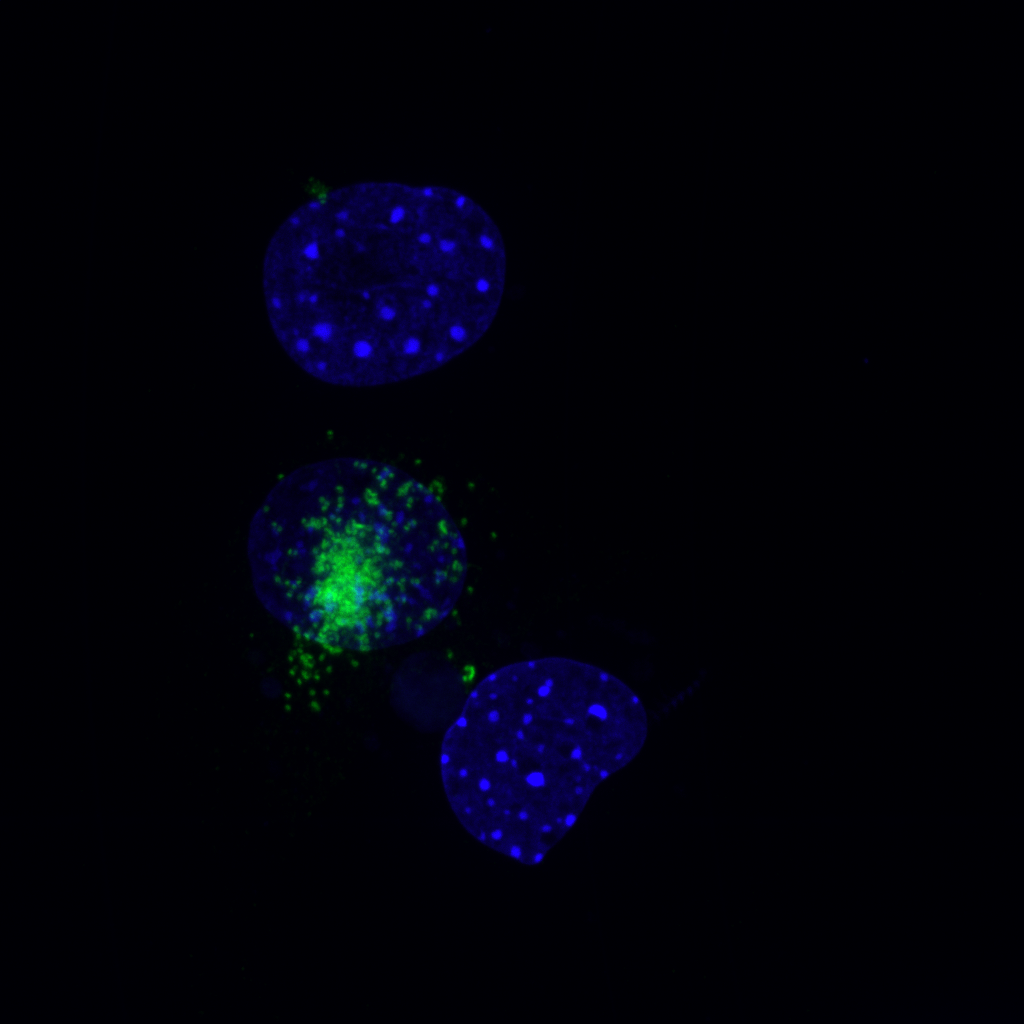

Supplement: Supplementary file 10 — Source data Fig. 6 [file 44319_2025_460_MOESM10_ESM.zip › Figure 6/F/MEF Dif5 Rapa Ad_MCIDAS_E2F4 DAPI_Blue FOP_Green.tif]

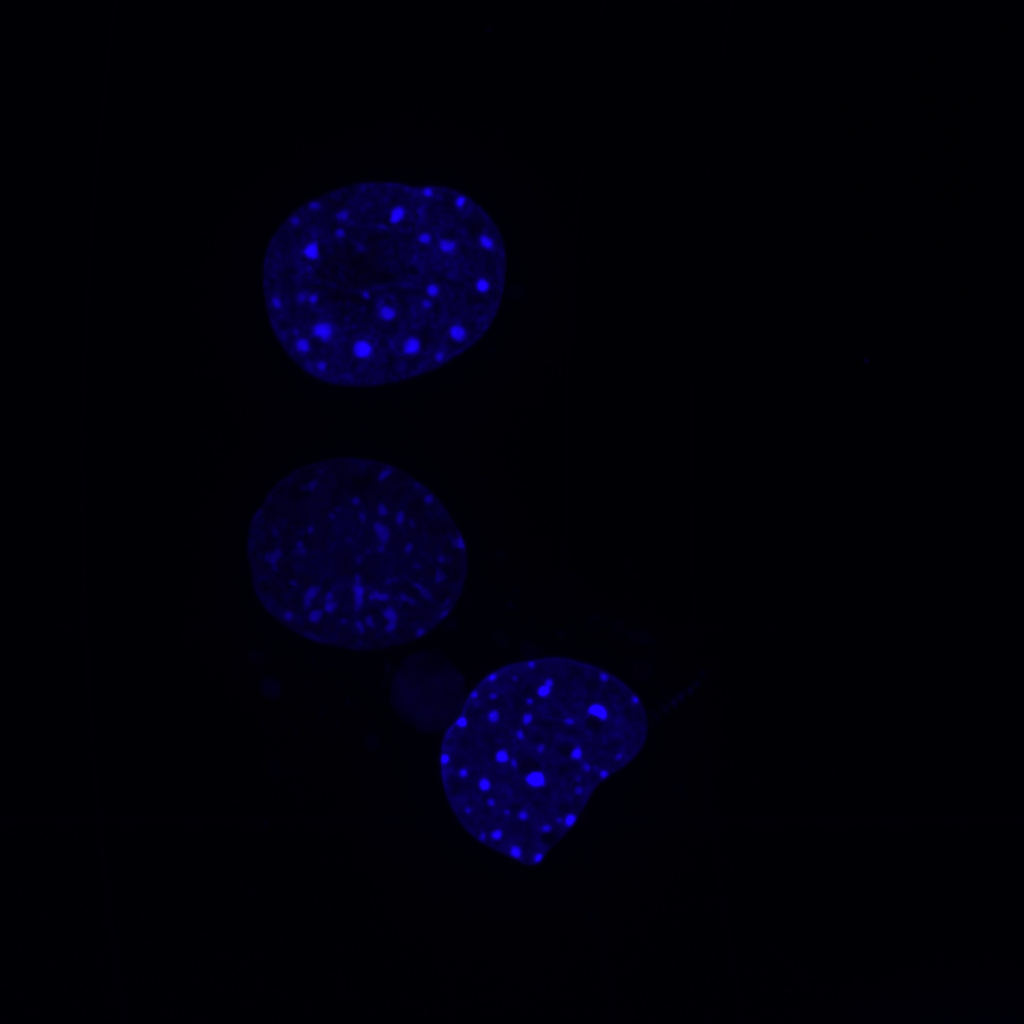

Supplement: Supplementary file 10 — Source data Fig. 6 [file 44319_2025_460_MOESM10_ESM.zip › Figure 6/F/MEF Dif5 Rapa Ad_MCIDAS_E2F4 DAPI_Blue.tif]

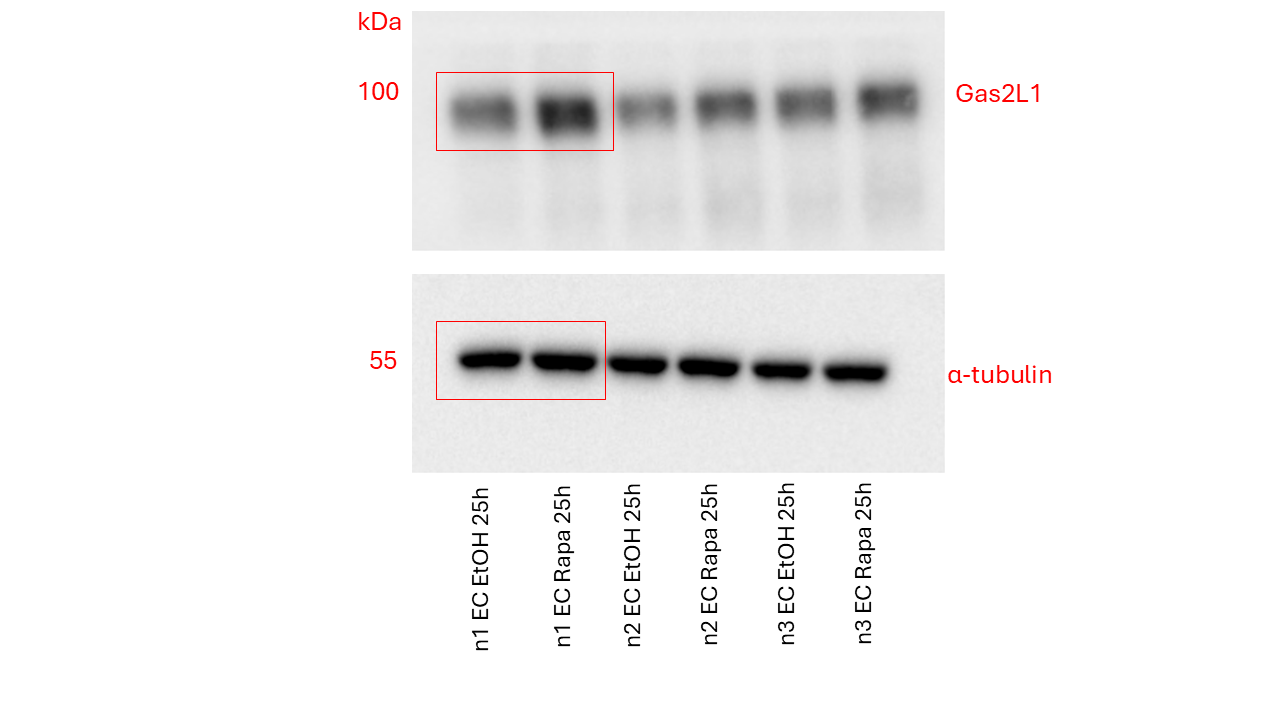

Supplement: Supplementary file 11 — Source data Fig. 7 [file 44319_2025_460_MOESM11_ESM.zip › Figure 7/D/Gas2L1 and alpha_tubulin.tif]

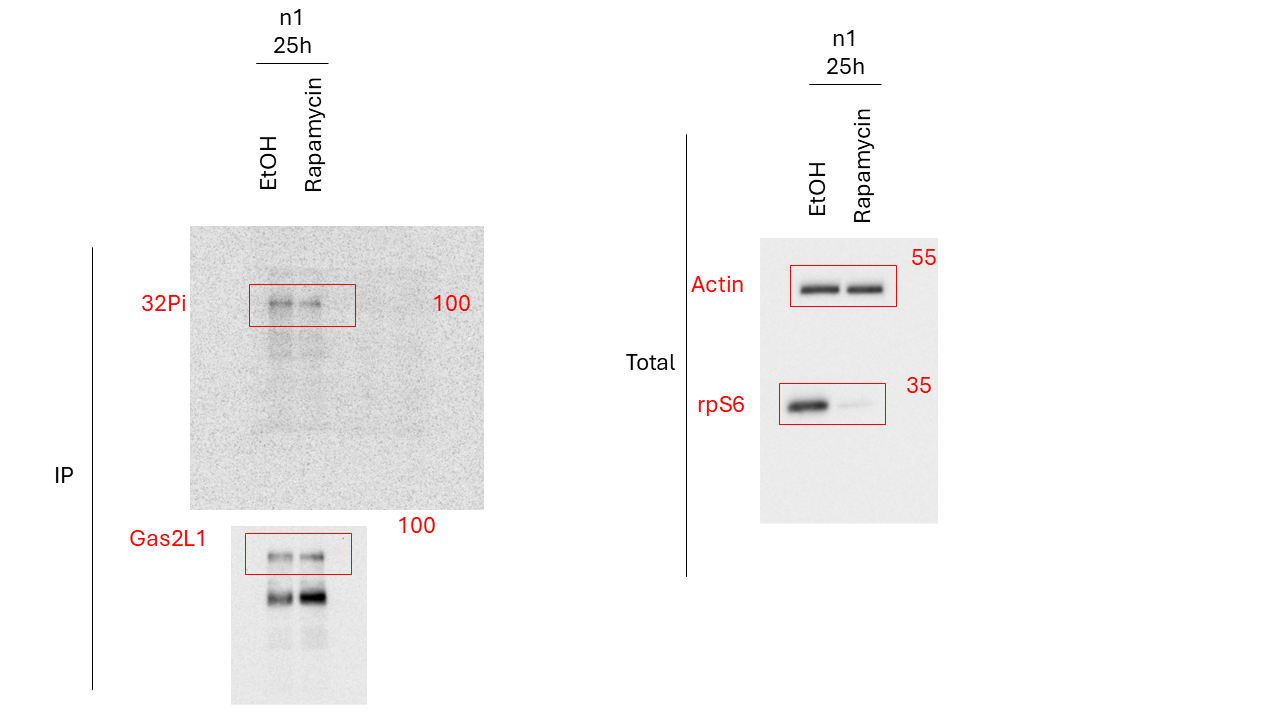

Supplement: Supplementary file 11 — Source data Fig. 7 [file 44319_2025_460_MOESM11_ESM.zip › Figure 7/F/F replicate 1.tif]

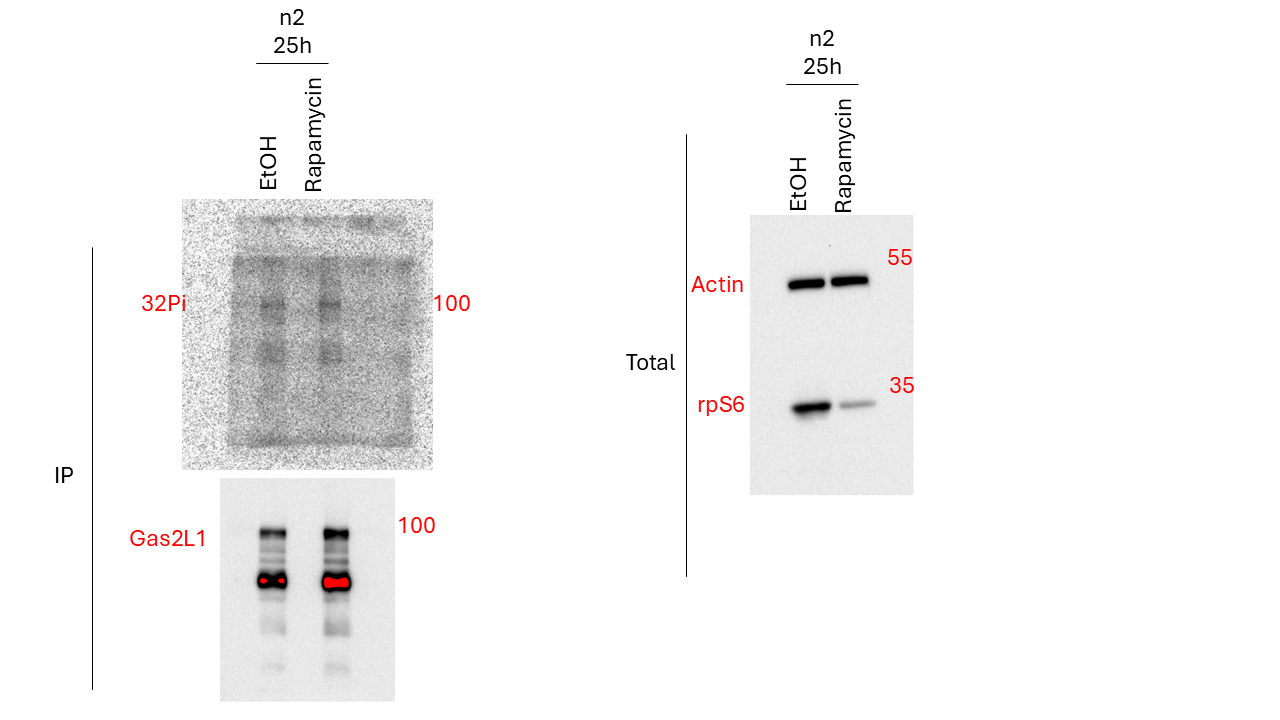

Supplement: Supplementary file 11 — Source data Fig. 7 [file 44319_2025_460_MOESM11_ESM.zip › Figure 7/F/F replicate 2.tif]

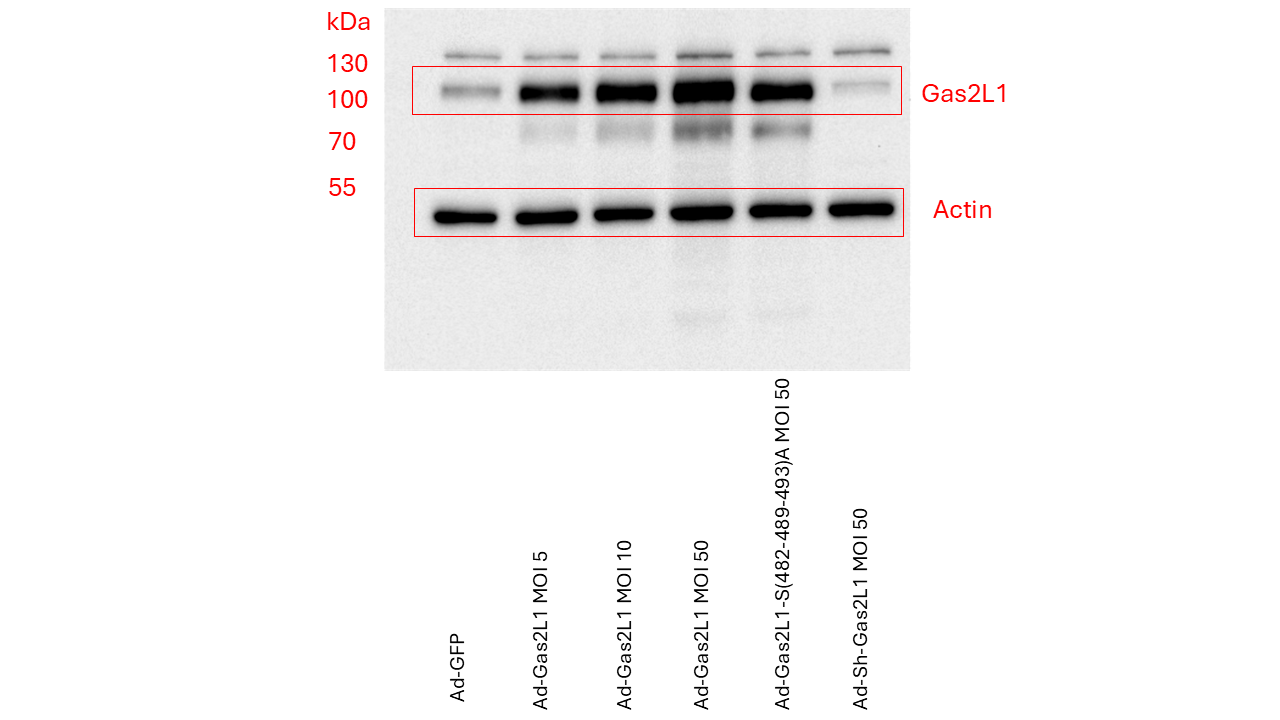

Supplement: Supplementary file 12 — Source data Fig. 8 [file 44319_2025_460_MOESM12_ESM.zip › Figure 8/A/Gas2L1.tif]
