# Supplementary figures and images for: mTOR controls ependymal cell differentiation by targeting the alternative cell cycle and centrosomal proteins (part 2 of 2)
Source: EMBO Rep. 2025 Apr 30;26(12):3075–105. doi: 10.1038/s44319-025-00460-2 (PMC12187940; doi:10.1038/s44319-025-00460-2)

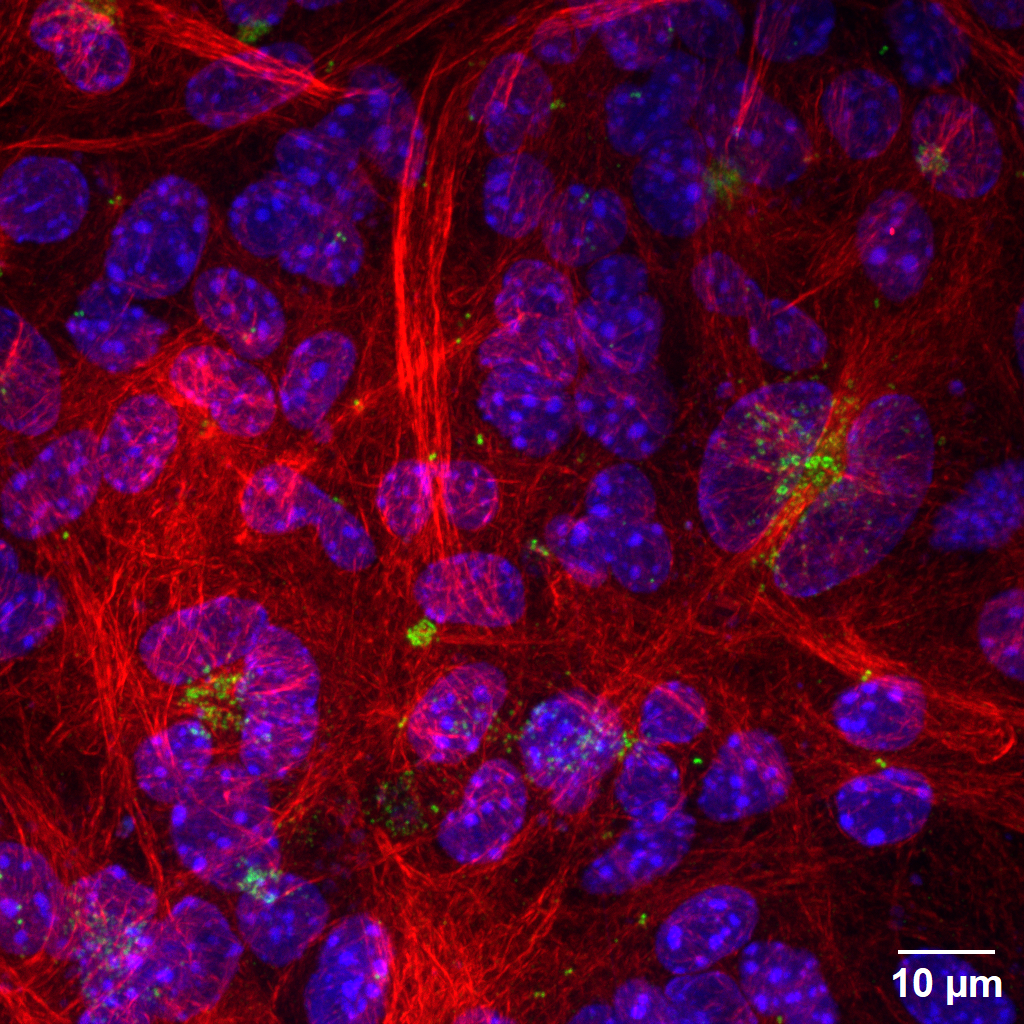

Supplement: Supplementary file 12 — Source data Fig. 8 [file 44319_2025_460_MOESM12_ESM.zip › Figure 8/B/raw images/EC Dif4 EtOH Ad_Gas2L1 DAPI_Blue Fop_Green Gas2L1_Red.tif]

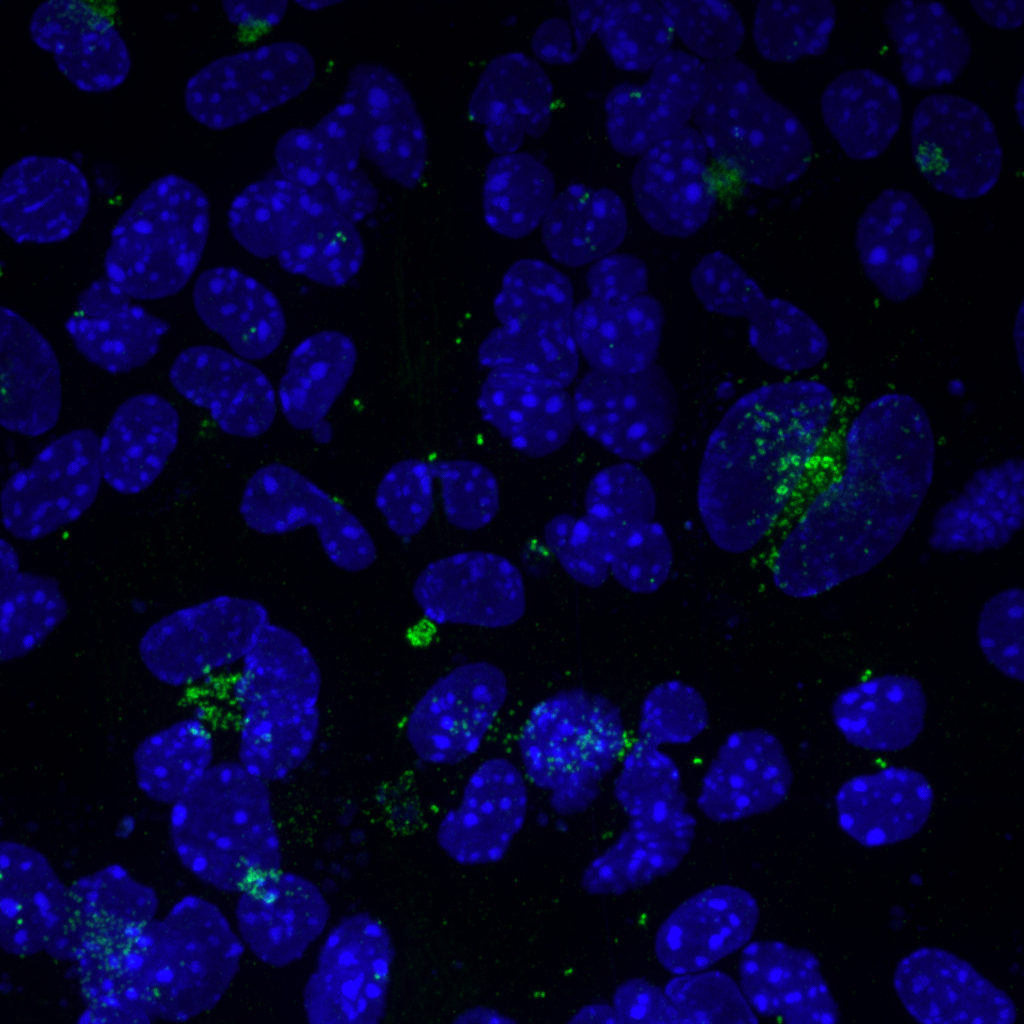

Supplement: Supplementary file 12 — Source data Fig. 8 [file 44319_2025_460_MOESM12_ESM.zip › Figure 8/B/raw images/EC Dif4 EtOH Ad_Gas2L1 DAPI_Blue Fop_Green.tif]

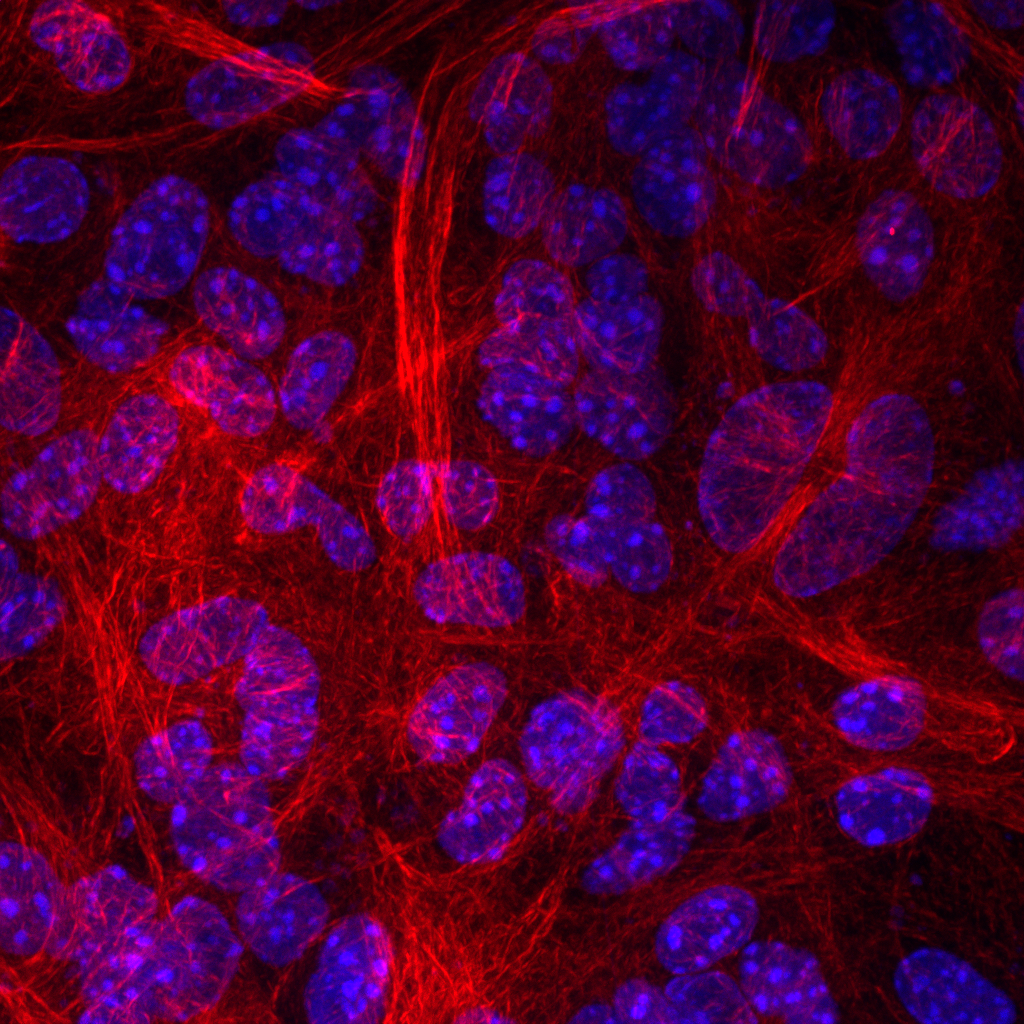

Supplement: Supplementary file 12 — Source data Fig. 8 [file 44319_2025_460_MOESM12_ESM.zip › Figure 8/B/raw images/EC Dif4 EtOH Ad_Gas2L1 DAPI_Blue Gas2L1_Red.tif]

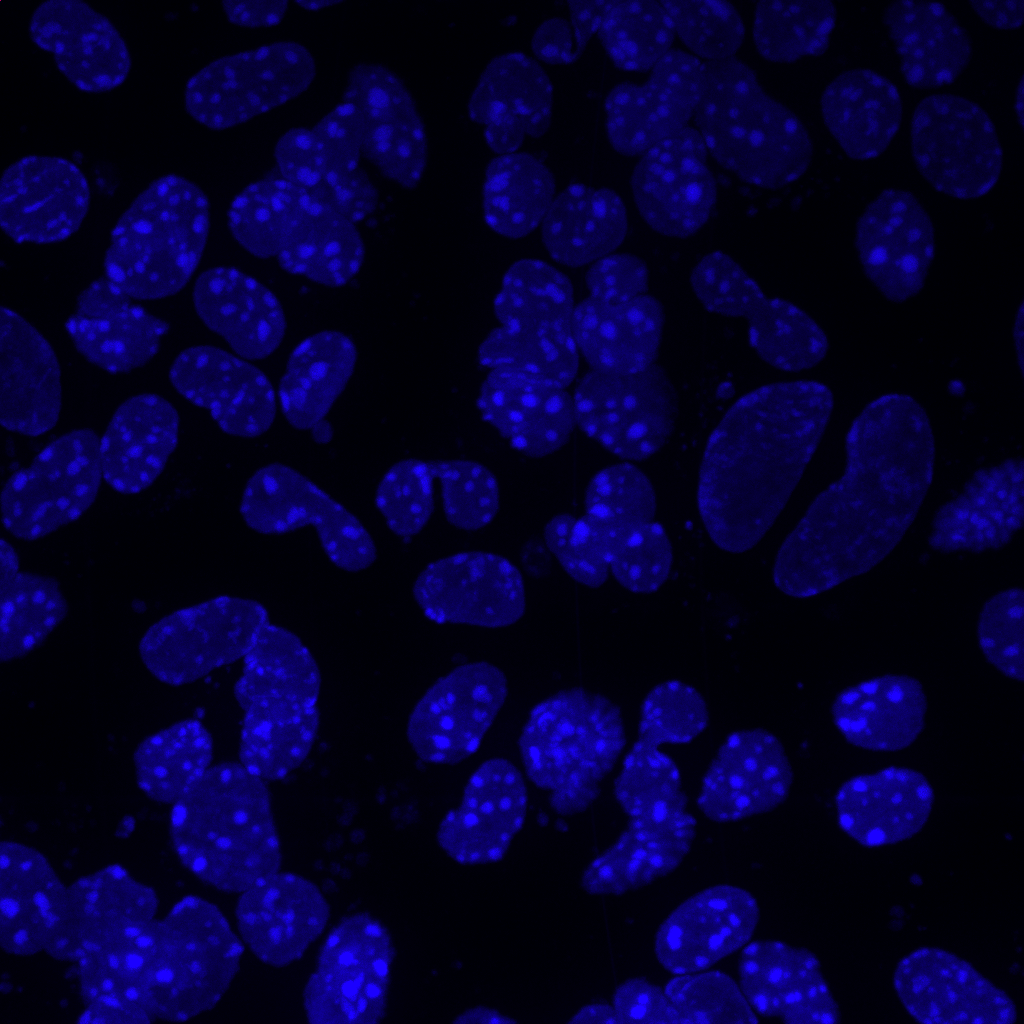

Supplement: Supplementary file 12 — Source data Fig. 8 [file 44319_2025_460_MOESM12_ESM.zip › Figure 8/B/raw images/EC Dif4 EtOH Ad_Gas2L1 DAPI_Blue.tif]

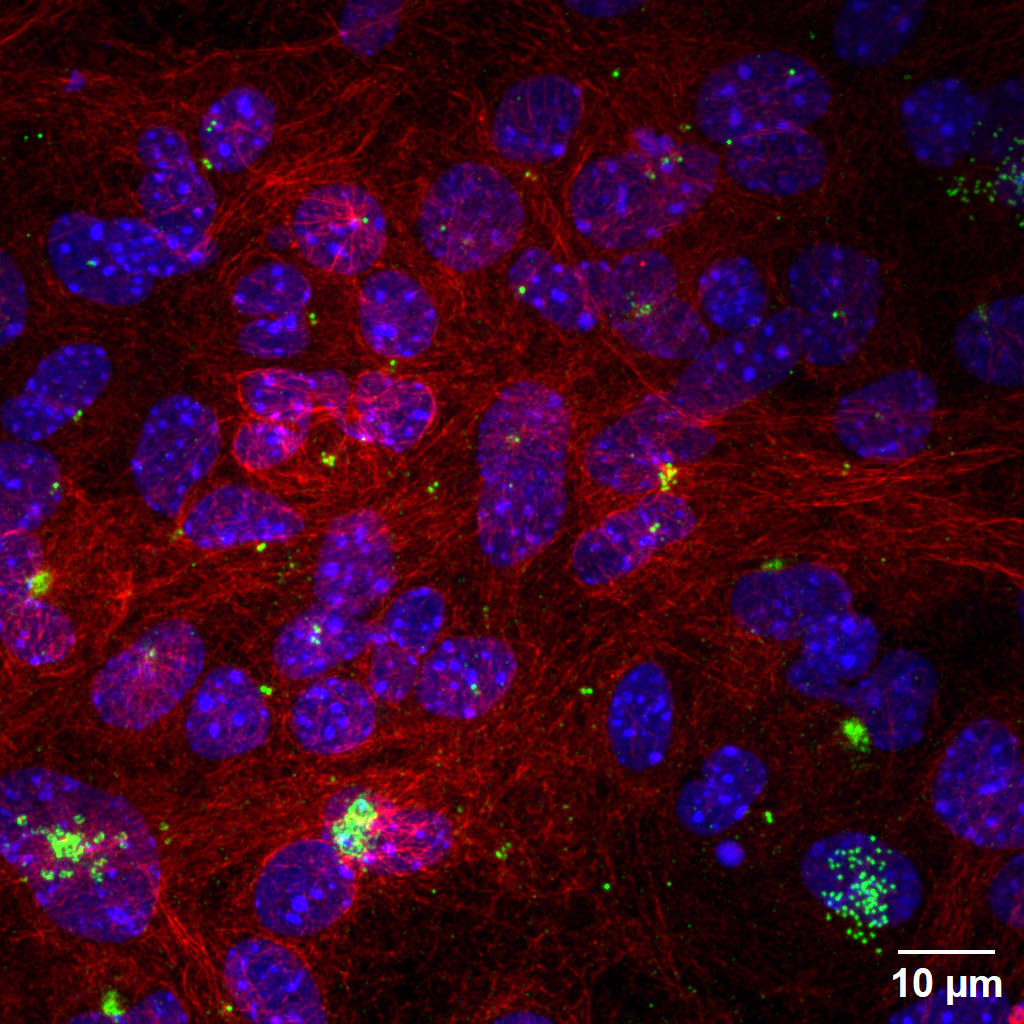

Supplement: Supplementary file 12 — Source data Fig. 8 [file 44319_2025_460_MOESM12_ESM.zip › Figure 8/B/raw images/EC Dif4 EtOH Ad_Gas2L1S482_489_493A DAPI_Blue Fop_Green Gas2L1_Red.tif]

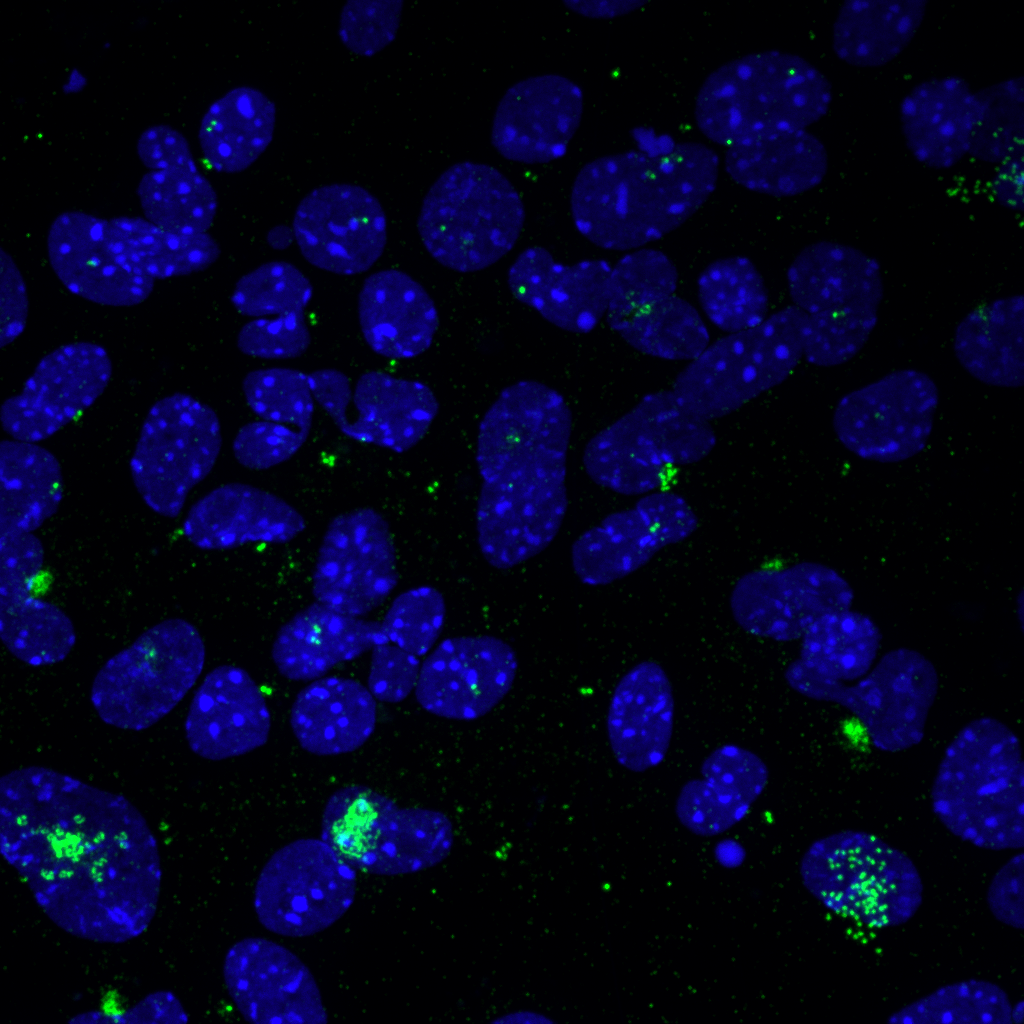

Supplement: Supplementary file 12 — Source data Fig. 8 [file 44319_2025_460_MOESM12_ESM.zip › Figure 8/B/raw images/EC Dif4 EtOH Ad_Gas2L1S482_489_493A DAPI_Blue Fop_Green.tif]

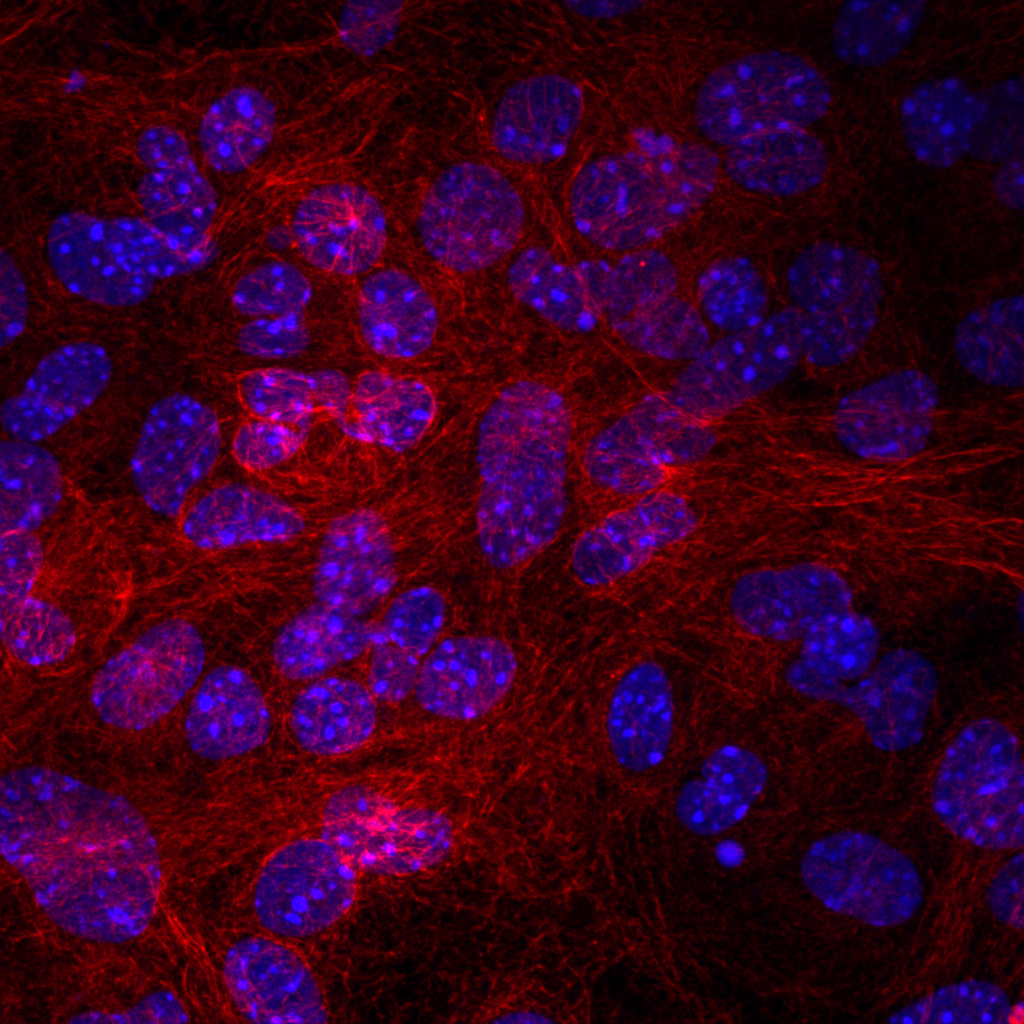

Supplement: Supplementary file 12 — Source data Fig. 8 [file 44319_2025_460_MOESM12_ESM.zip › Figure 8/B/raw images/EC Dif4 EtOH Ad_Gas2L1S482_489_493A DAPI_Blue Gas2L1_Red.tif]

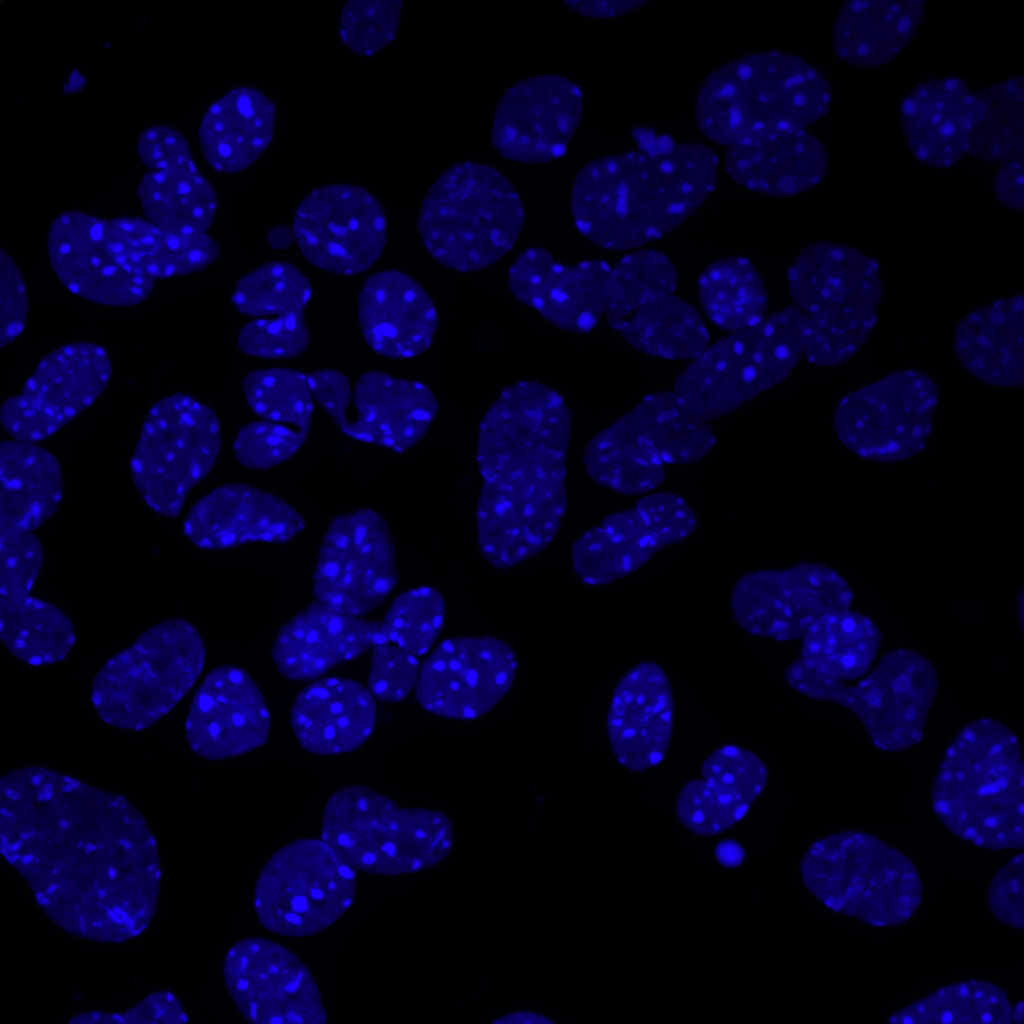

Supplement: Supplementary file 12 — Source data Fig. 8 [file 44319_2025_460_MOESM12_ESM.zip › Figure 8/B/raw images/EC Dif4 EtOH Ad_Gas2L1S482_489_493A DAPI_Blue.tif]

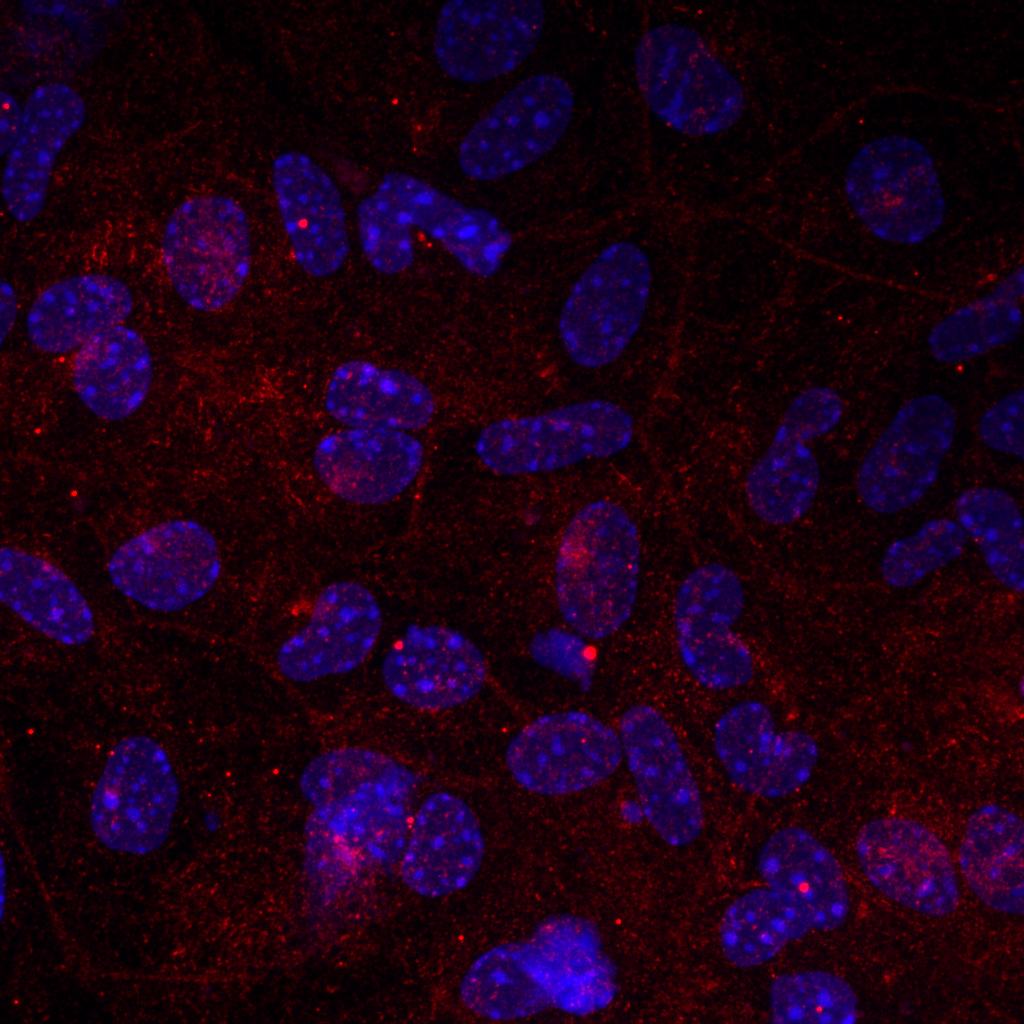

Supplement: Supplementary file 12 — Source data Fig. 8 [file 44319_2025_460_MOESM12_ESM.zip › Figure 8/B/raw images/EC Dif4 EtOH Ad_GFP DAPI_Blue Gas2L1_Red.tif]

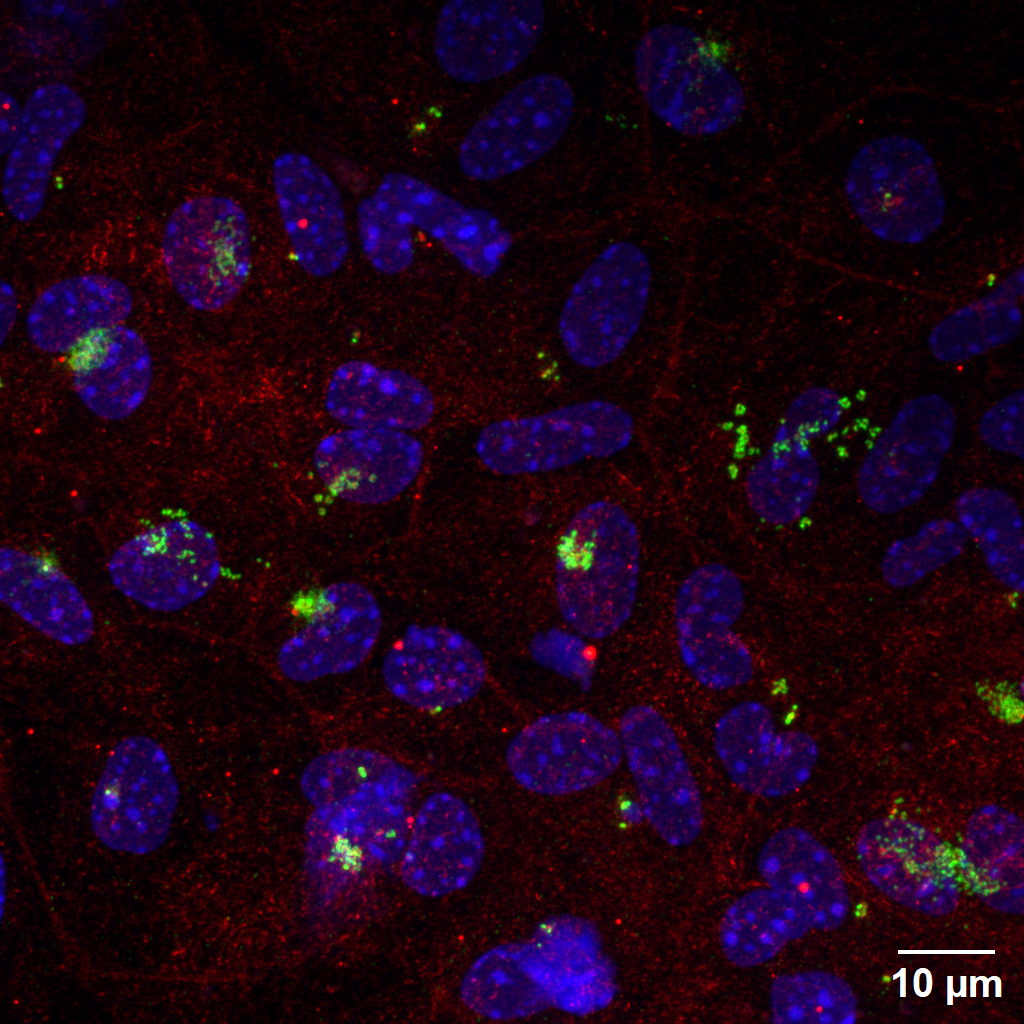

Supplement: Supplementary file 12 — Source data Fig. 8 [file 44319_2025_460_MOESM12_ESM.zip › Figure 8/B/raw images/EC Dif4 EtOH Ad_GFP DAPI_Blue Fop_Green Gas2L1_Red.tif]

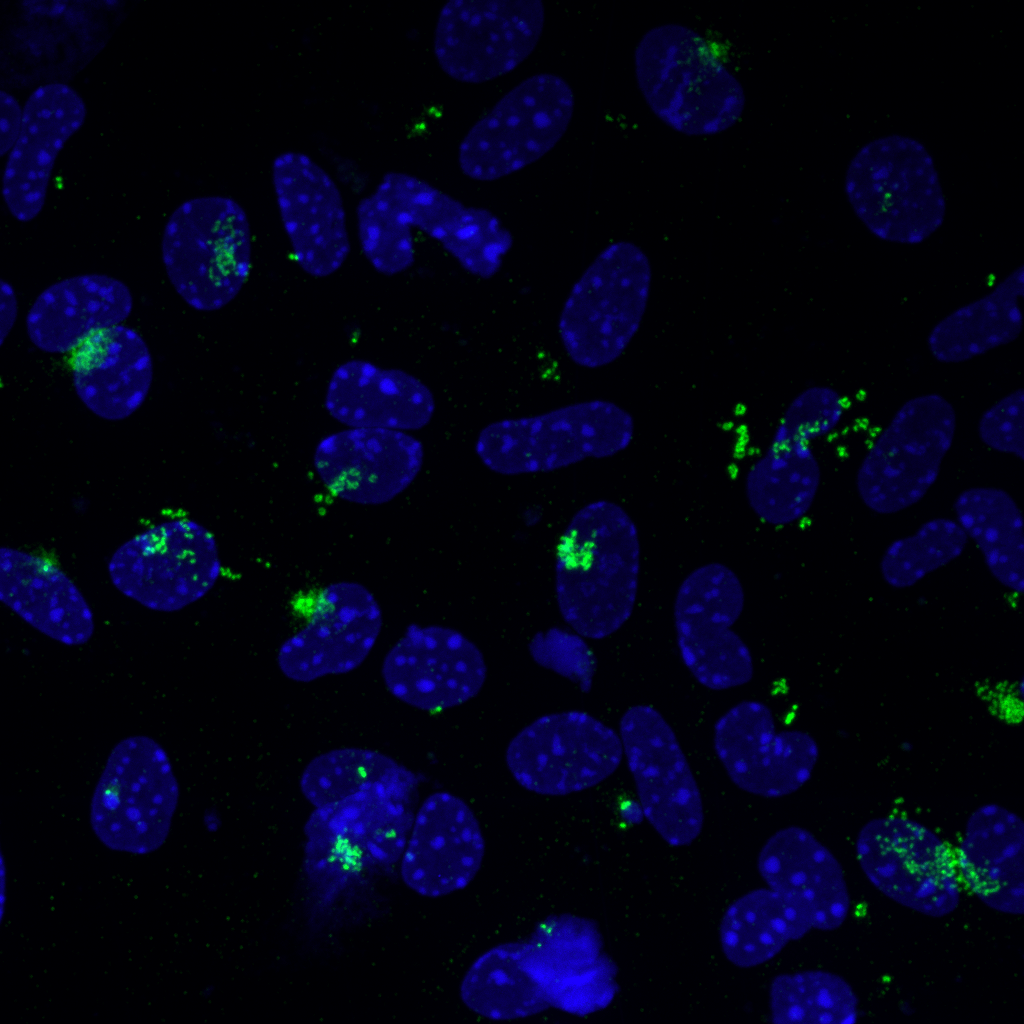

Supplement: Supplementary file 12 — Source data Fig. 8 [file 44319_2025_460_MOESM12_ESM.zip › Figure 8/B/raw images/EC Dif4 EtOH Ad_GFP DAPI_Blue Fop_Green.tif]

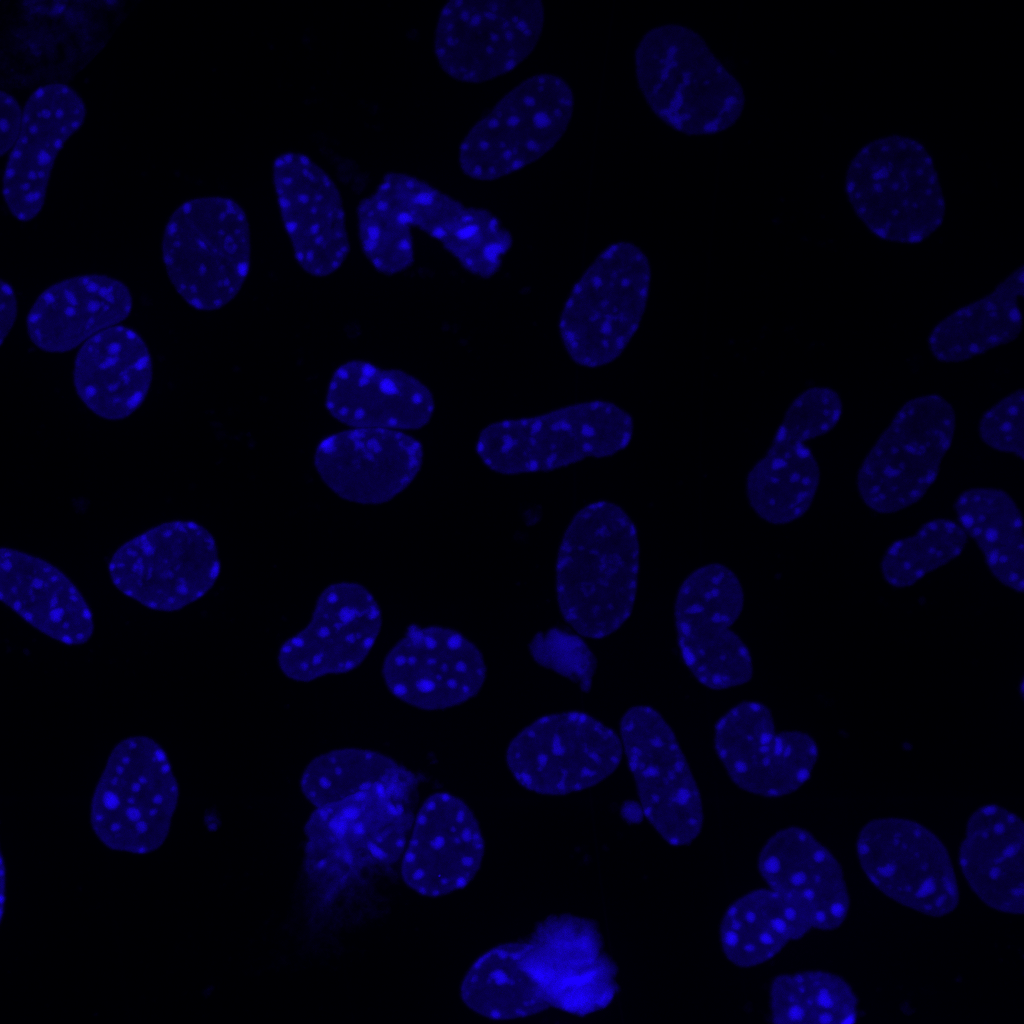

Supplement: Supplementary file 12 — Source data Fig. 8 [file 44319_2025_460_MOESM12_ESM.zip › Figure 8/B/raw images/EC Dif4 EtOH Ad_GFP DAPI_Blue.tif]

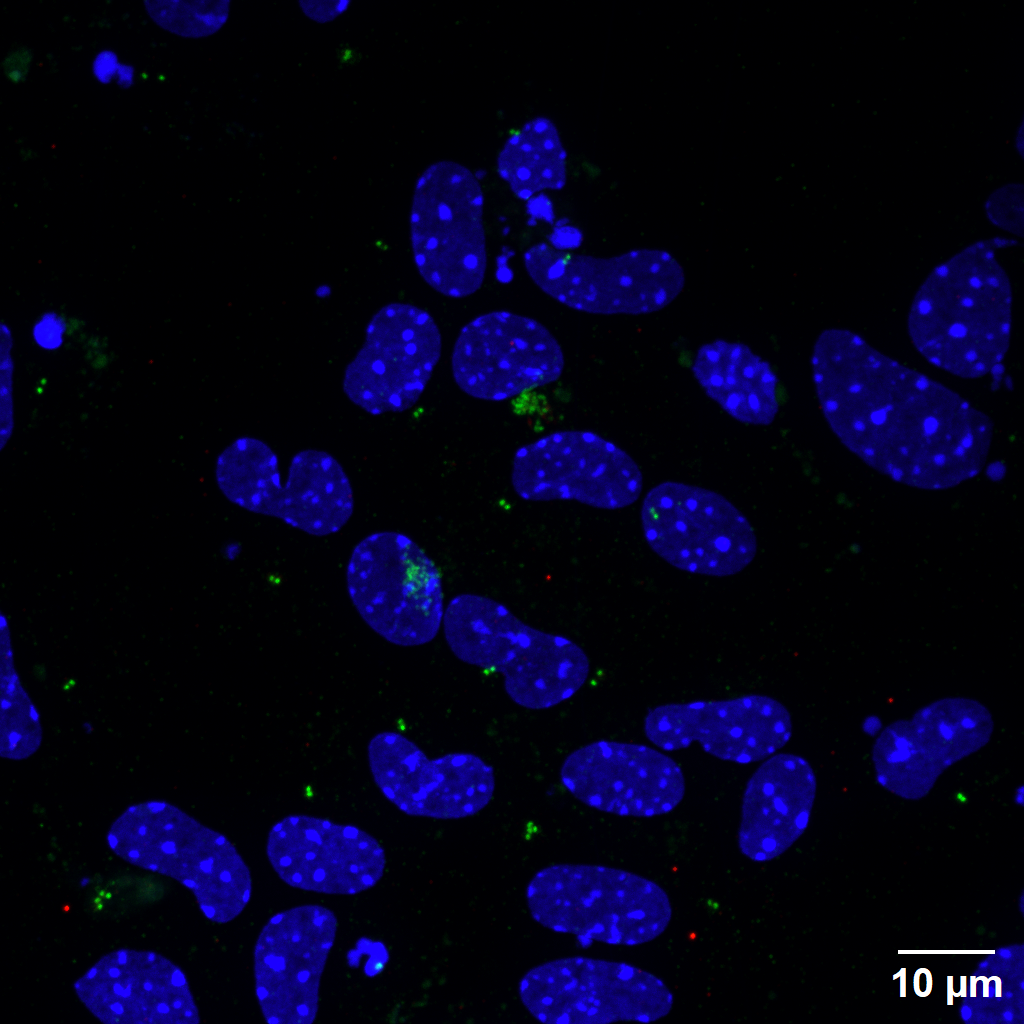

Supplement: Supplementary file 12 — Source data Fig. 8 [file 44319_2025_460_MOESM12_ESM.zip › Figure 8/B/raw images/EC Dif4 EtOH Ad_shGas2L1 DAPI_Blue Fop_Green Gas2L1_Red.tif]

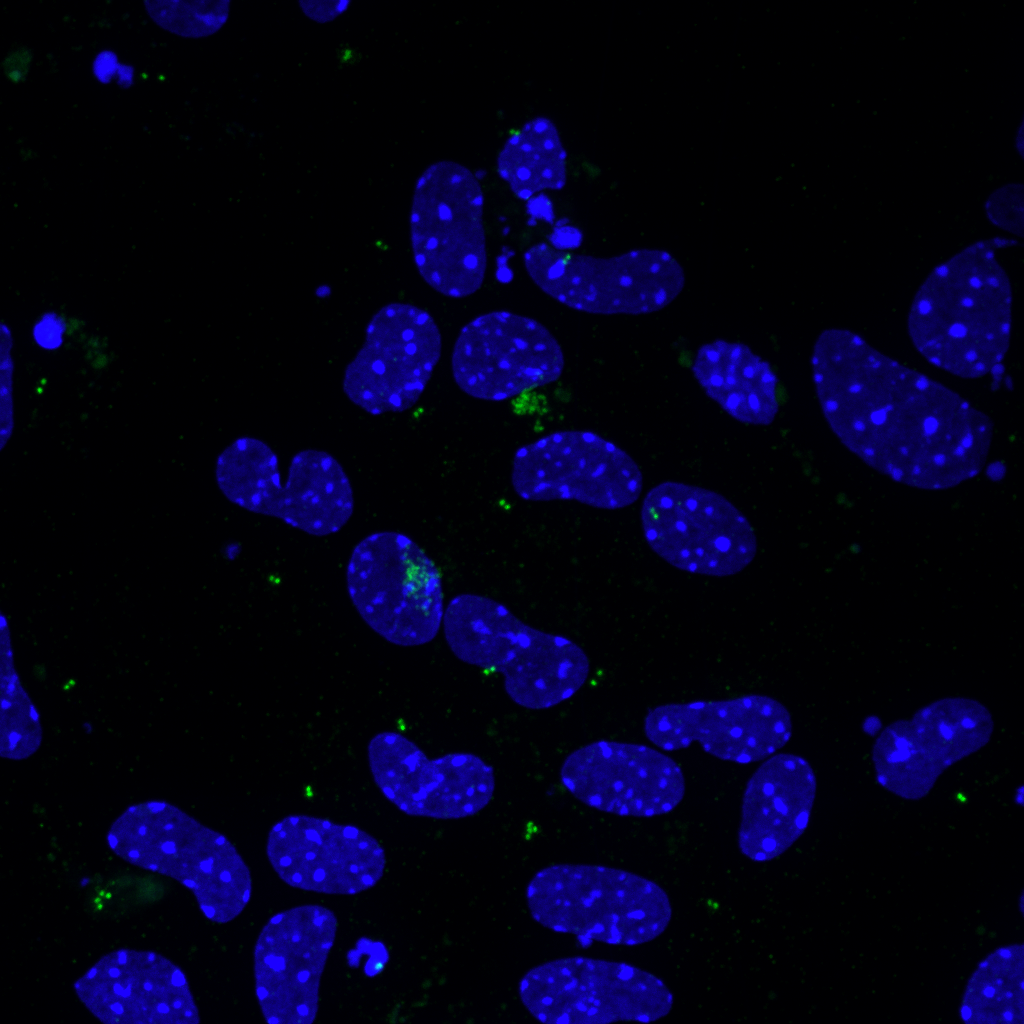

Supplement: Supplementary file 12 — Source data Fig. 8 [file 44319_2025_460_MOESM12_ESM.zip › Figure 8/B/raw images/EC Dif4 EtOH Ad_shGas2L1 DAPI_Blue Fop_Green.tif]

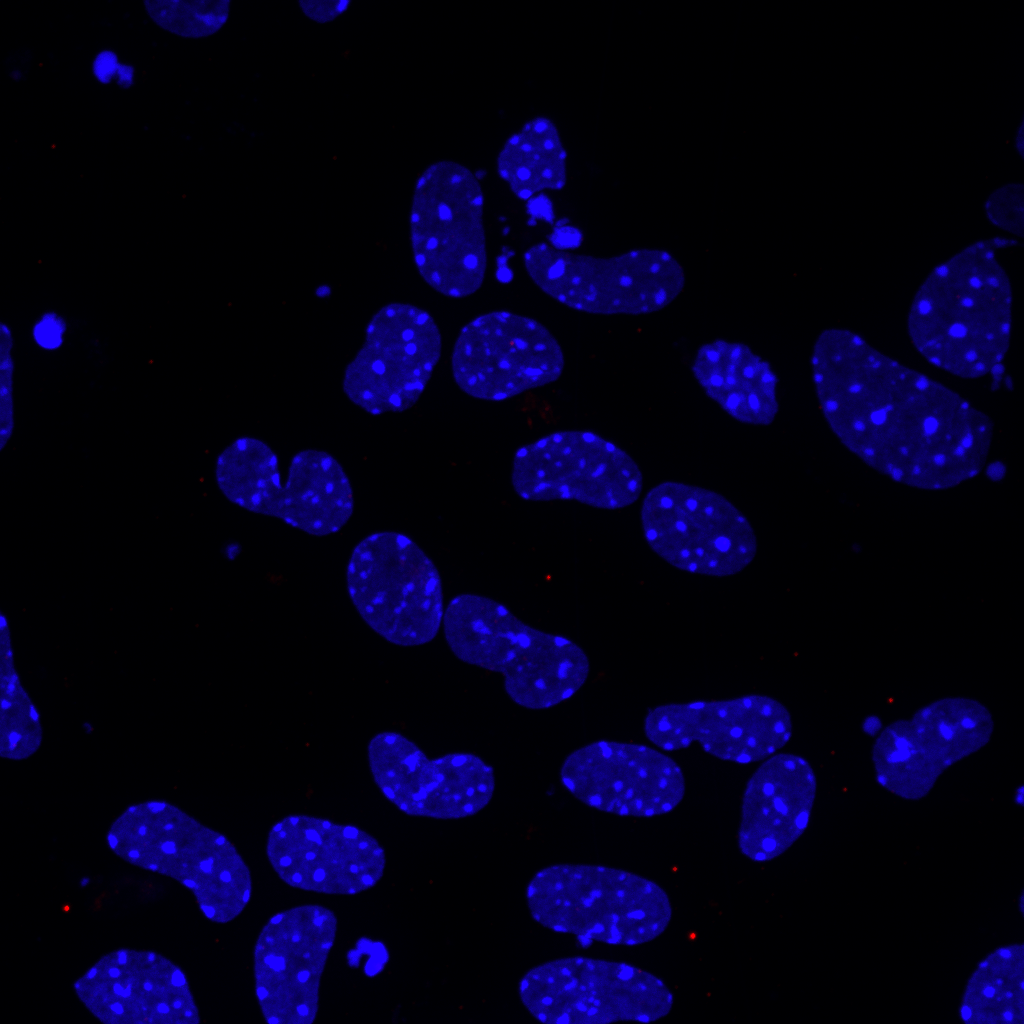

Supplement: Supplementary file 12 — Source data Fig. 8 [file 44319_2025_460_MOESM12_ESM.zip › Figure 8/B/raw images/EC Dif4 EtOH Ad_shGas2L1 DAPI_Blue Gas2L1_Red.tif]

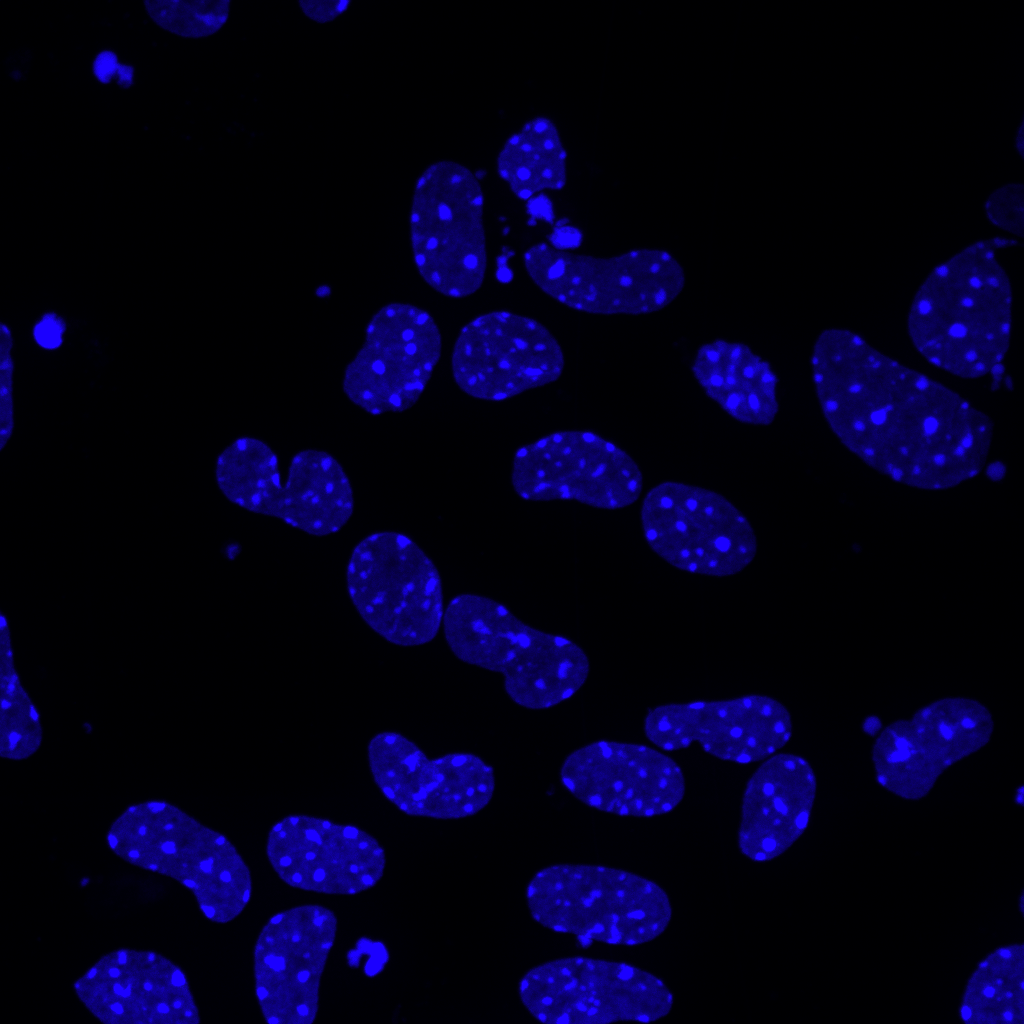

Supplement: Supplementary file 12 — Source data Fig. 8 [file 44319_2025_460_MOESM12_ESM.zip › Figure 8/B/raw images/EC Dif4 EtOH Ad_shGas2L1 DAPI_Blue.tif]

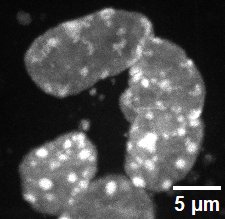

Supplement: Supplementary file 12 — Source data Fig. 8 [file 44319_2025_460_MOESM12_ESM.zip › Figure 8/B/zoom images/EC Dif4 Ad_Gas2L1 DAPI.tif]

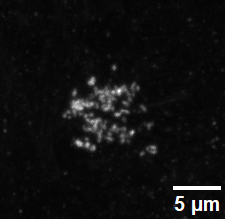

Supplement: Supplementary file 12 — Source data Fig. 8 [file 44319_2025_460_MOESM12_ESM.zip › Figure 8/B/zoom images/EC Dif4 Ad_Gas2L1 FOP.tif]

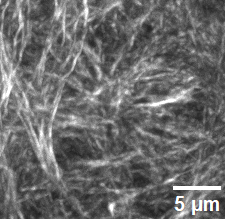

Supplement: Supplementary file 12 — Source data Fig. 8 [file 44319_2025_460_MOESM12_ESM.zip › Figure 8/B/zoom images/EC Dif4 Ad_Gas2L1 Gas2L1.tif]

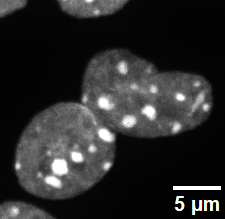

Supplement: Supplementary file 12 — Source data Fig. 8 [file 44319_2025_460_MOESM12_ESM.zip › Figure 8/B/zoom images/EC Dif4 Ad_Gas2L1S482_489_493A DAPI.tif]

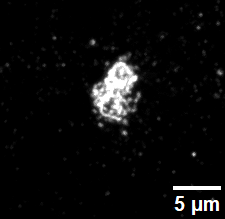

Supplement: Supplementary file 12 — Source data Fig. 8 [file 44319_2025_460_MOESM12_ESM.zip › Figure 8/B/zoom images/EC Dif4 Ad_Gas2L1S482_489_493A FOP.tif]

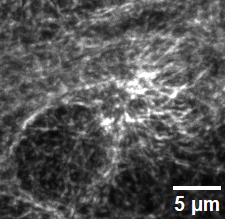

Supplement: Supplementary file 12 — Source data Fig. 8 [file 44319_2025_460_MOESM12_ESM.zip › Figure 8/B/zoom images/EC Dif4 Ad_Gas2L1S482_489_493A Gas2L1.tif]

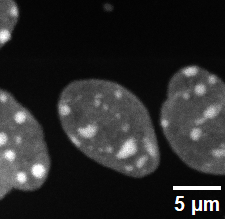

Supplement: Supplementary file 12 — Source data Fig. 8 [file 44319_2025_460_MOESM12_ESM.zip › Figure 8/B/zoom images/EC Dif4 Ad_GFP DAPI.tif]

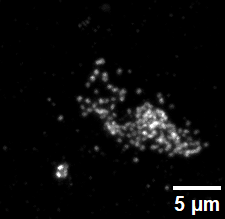

Supplement: Supplementary file 12 — Source data Fig. 8 [file 44319_2025_460_MOESM12_ESM.zip › Figure 8/B/zoom images/EC Dif4 Ad_GFP FOP.tif]

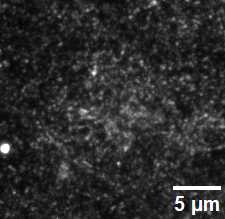

Supplement: Supplementary file 12 — Source data Fig. 8 [file 44319_2025_460_MOESM12_ESM.zip › Figure 8/B/zoom images/EC Dif4 Ad_GFP Gas2L1.tif]

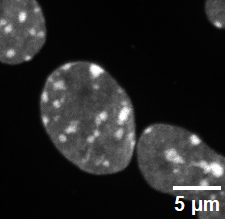

Supplement: Supplementary file 12 — Source data Fig. 8 [file 44319_2025_460_MOESM12_ESM.zip › Figure 8/B/zoom images/EC Dif4 Ad_sh_Gas2L1 DAPI.tif]

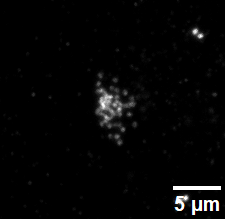

Supplement: Supplementary file 12 — Source data Fig. 8 [file 44319_2025_460_MOESM12_ESM.zip › Figure 8/B/zoom images/EC Dif4 Ad_sh_Gas2L1 FOP.tif]

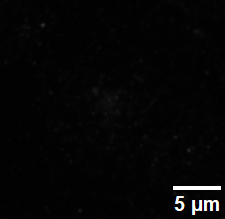

Supplement: Supplementary file 12 — Source data Fig. 8 [file 44319_2025_460_MOESM12_ESM.zip › Figure 8/B/zoom images/EC Dif4 Ad_sh_Gas2L1 Gas2L1.tif]
