# Supplementary material for: mTOR controls ependymal cell differentiation by targeting the alternative cell cycle and centrosomal proteins
Source: EMBO Rep. 2025 Apr 30;26(12):3075–105. doi: 10.1038/s44319-025-00460-2 (PMC12187940; doi:10.1038/s44319-025-00460-2)
Supplement: Supplementary file 13 — Expanded View Figures [file 44319_2025_460_MOESM13_ESM.pdf]

Expanded View Figures

Figure EV1. mTORC1 signaling pathway is active during centriole amplification.

(**A**, **B**) P4 brain lateral ventricular en face, labeling Centrin (centrioles, white), p-rpS6 (red) and GT335 (cilia, green) or p21 (green) in (**A**, **B**), respectively. (**C**) Ependymal cells in respective phases of differentiation characterized by their staining of Centrin (centrioles, white) and GT335 (cilia, green), and p-rpS6 (red). It is worth noting that the expression of p-rpS6 is during the intermediate stages of differentiation. (**D**) Regression plot testing the correlation between apical area and centriole number in mature ependymal cells in Tsc1 cKO at P0, rapamycin injected pups at P4 and their respective controls, R^2 is the correlation coefficient; ns $P = 0.0542$; $^{**}P = 0.0023$; $^{***}P = 0.0001$; $^{****}P < 0,0001$. Data information: In (**D**), $^{**}P < 0.01$, $^{***}P < 0.001$, $^{****}P < 0.0001$ (Pearson's correlation test). n = number of cells >50. Scale bars: 10 µm.

 © The Author(s)

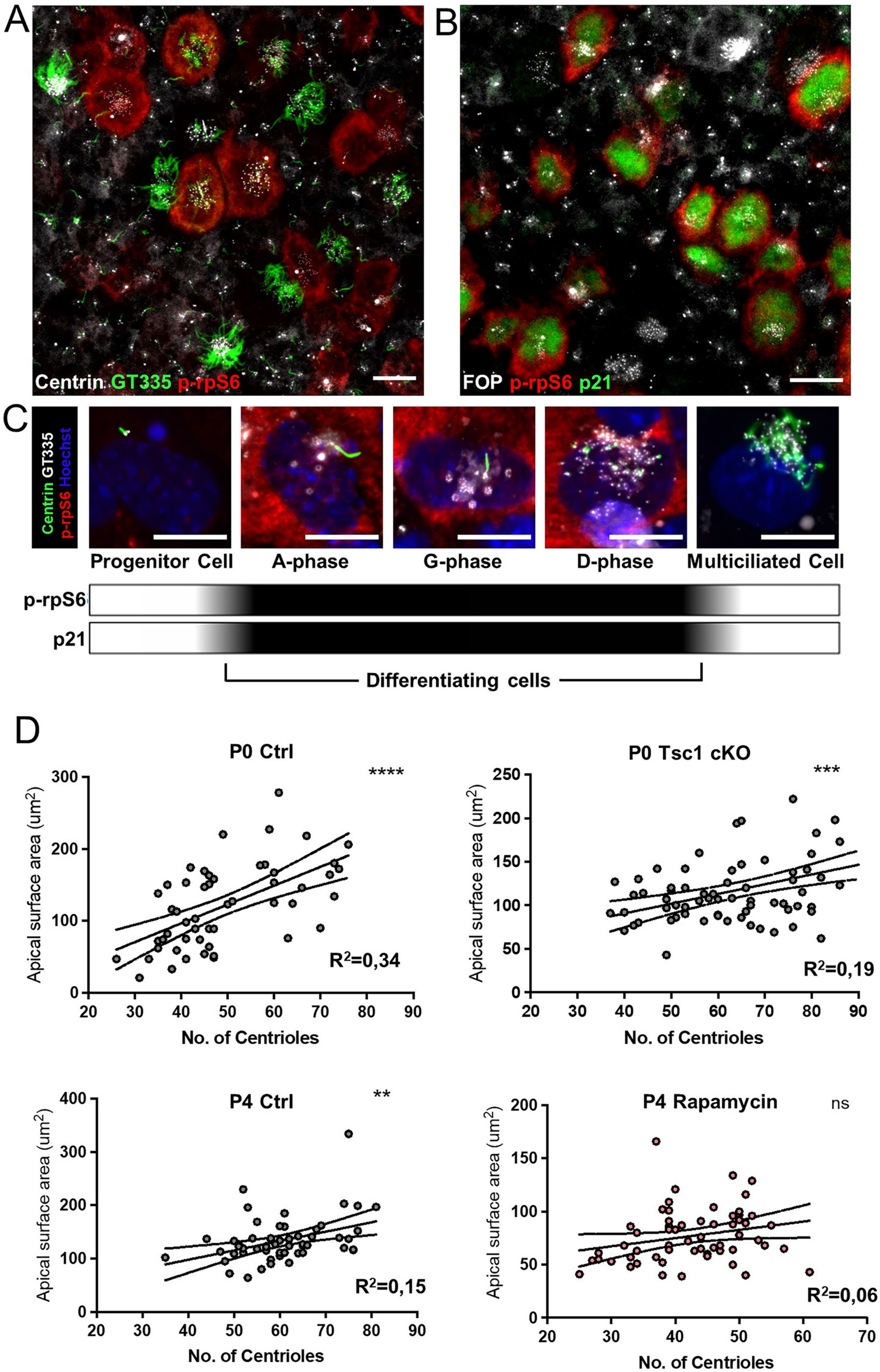
A
Centrin GT335 p-rpS6
B
FOP p-rpS6 p21
C
Centrin GT335 p-rpS6 Hoechst
Progenitor Cell
A-phase
G-phase
D-phase
Multiciliated Cell
p-rpS6
p21
Differentiating cells
D
P0 Ctrl
****
R2=0,34
P0 Tsc1 cKO
***
R2=0,19
P4 Ctrl
**
R2=0,15
P4 Rapamycin
ns
R2=0,06
Apical surface area (um2)
No. of Centrioles

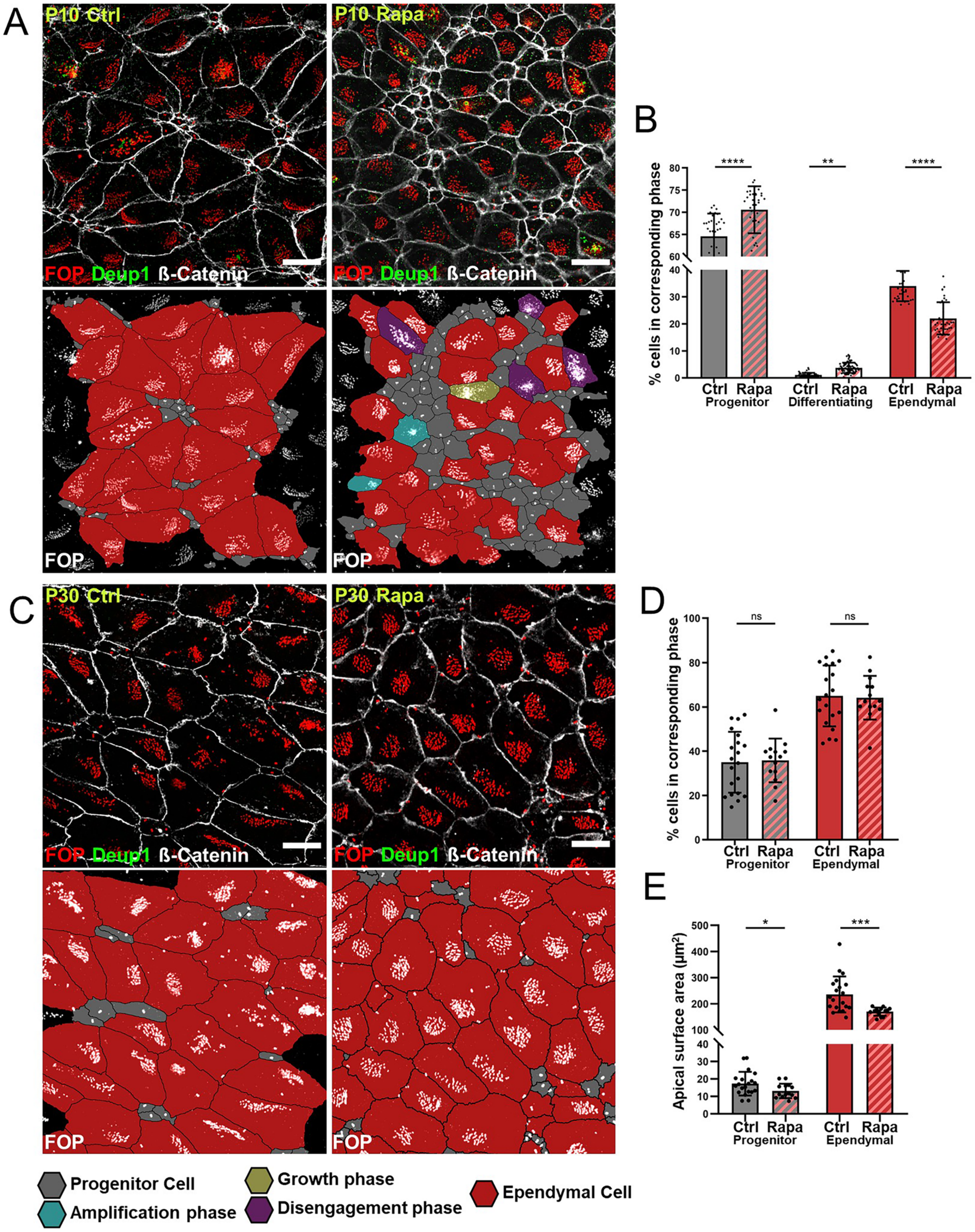

Figure EV2. Long-term effect of rapamycin treatment.

(**A**) Lateral ventricular wall of mice at P10 injected with rapamycin every day between P0 and P4 and labeled with Fop (centrioles, red), Deup (deuterosomes, green) and β-Catenin (cell junction, white). Corresponding segmented images are shown below. (**B**) Percentage of cells at each stage of differentiation at P10; $^{**}P = 0.0022$; $^{****}P < 0.0001$. (**C**) Lateral ventricular wall of mice at P30 injected with rapamycin every day between P0 and P4 and labeled with Fop (centrioles, red), Deup (deuterosomes, green) and β-Catenin (cell junction, white). Corresponding segmented images are shown below. (**D**) Percentage of cells at each stage of differentiation at P30; ns $P = 0.9039$. (**E**) Quantification of the size of the apical surface of progenitor and mature ependymal cells in rapamycin and control conditions at P30; $^{*}P = 0.0470$; $^{***}P = 0.0005$. Scale bars: 8.5 µm. Data information: In (**B**, **D**, **E**), data are presented as mean ± SD. $^{****}P < 0.0001$, $^{***}P < 0.001$, $^{**}P < 0.01$, $^{*}P \leq 0.05$, ns not significant (Student's t test).

 © The Author(s)

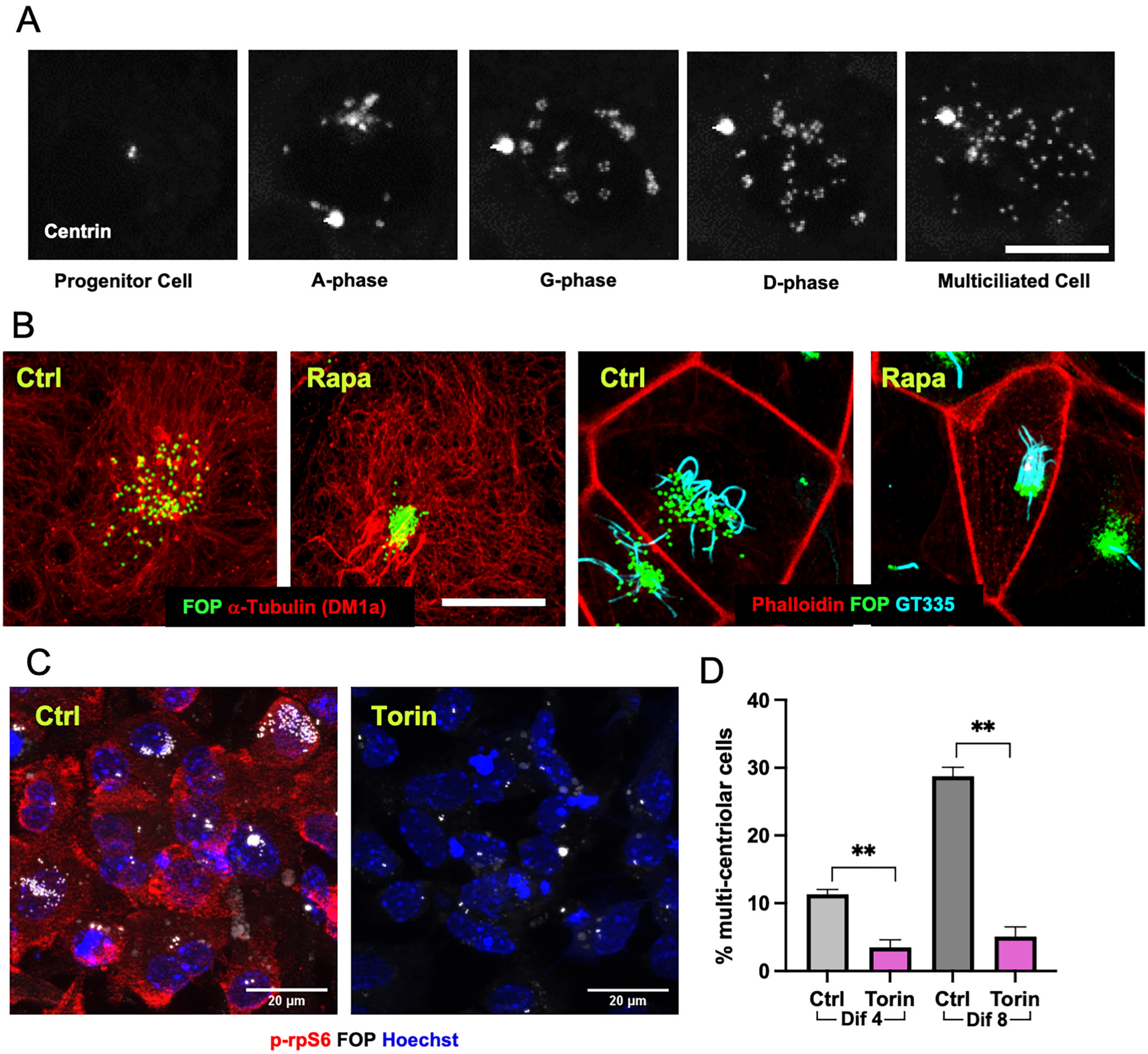

Figure EV3. In vitro stages of ependymal differentiation.

(**A**) Representative images of Cen2GFP+ cells at different stages of basal body formation during ependymal cell differentiation in vitro. (**B**) Representative images of primary cells at div 4 immunolabeled with FOP and α-Tubulin (DM1a) or Phalloidin and GT335 in control and rapamycin conditions. (**C**) Representative images of primary cells at dif 4 in control and Torin conditions. Control is the same as in Fig. EV4D, as the experiments were carried out in parallel. (**D**) Percentage of multicentriolar cells at different time points in the indicated conditions; $**P = 0.0022$. Data information: (**D**) presents data as mean ± SEM. $**P < 0.01$ (Mann–Whitney test); $n = 3$. Scale bars: 10 μm (**A**, **B**), 20 μm (**C**).

© The Author(s)

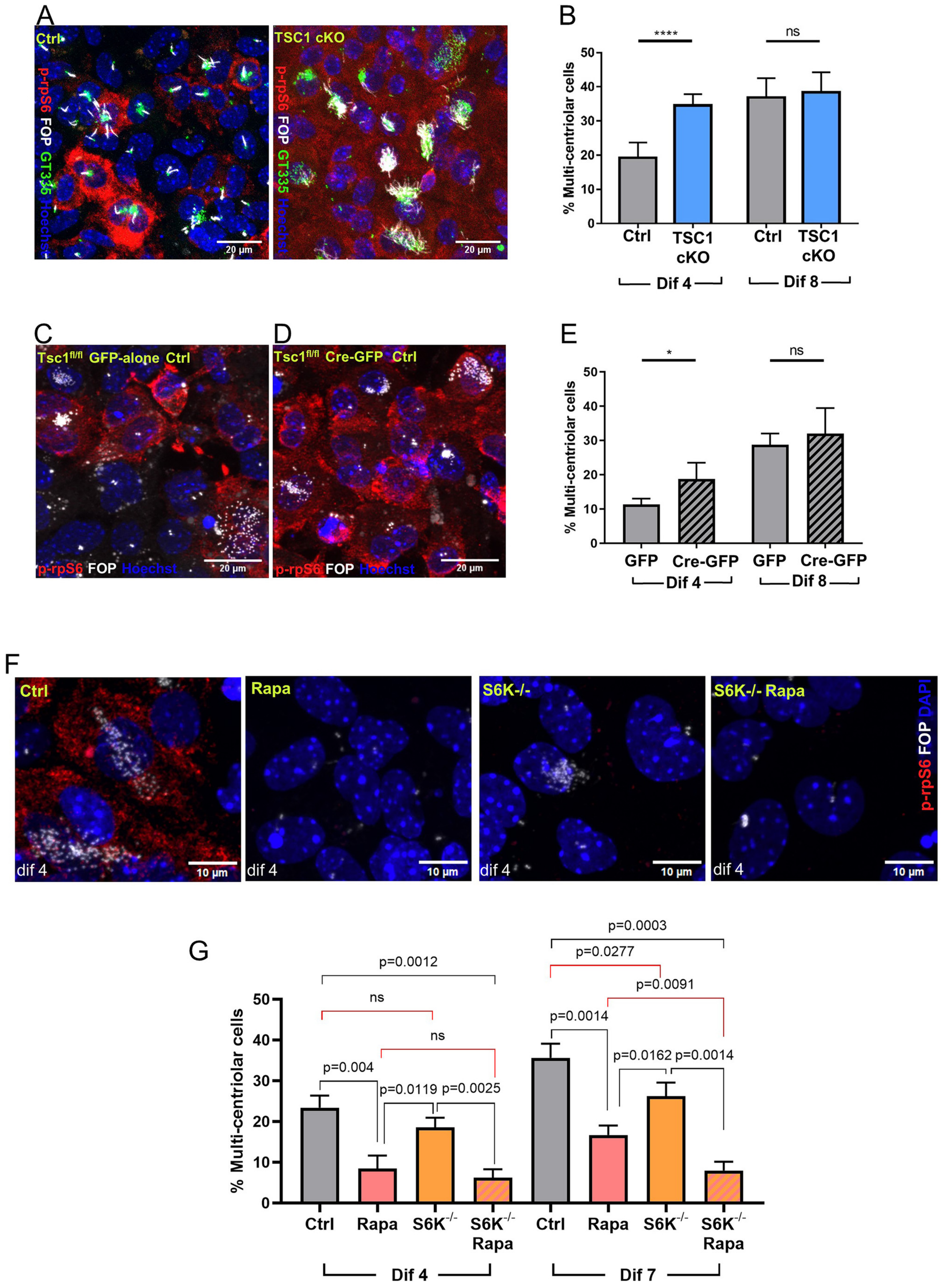
A
Ctrl
p-rpS6 FOP GT335 Hoechst
20 µm
TSC1 cKO
p-rpS6 FOP GT335 Hoechst
20 µm
B
% Multi-centriolar cells
****
ns
Ctrl
TSC1 cKO
Ctrl
TSC1 cKO
Dif 4
Dif 8
C
Tsc1fl/fl GFP-alone Ctrl
p-rpS6 FOP Hoechst
20 µm
D
Tsc1fl/fl Cre-GFP Ctrl
p-rpS6 FOP Hoechst
20 µm
E
% Multi-centriolar cells
*
ns
GFP
Cre-GFP
GFP
Cre-GFP
Dif 4
Dif 8
F
Ctrl
dif 4
10 µm
Rapa
dif 4
10 µm
S6K-/-
dif 4
10 µm
S6K-/- Rapa
dif 4
10 µm
p-rpS6 FOP DAPI
G
% Multi-centriolar cells
p=0.0012
ns
ns
p=0.004
p=0.0119 p=0.0025
p=0.0003
p=0.0277
p=0.0091
p=0.0014
p=0.0162 p=0.0014
Ctrl
Rapa
S6K-/-
S6K-/- Rapa
Ctrl
Rapa
S6K-/-
S6K-/- Rapa
Dif 4
Dif 7

 © The Author(s)

Figure EV4. Inactivation of Tsc1 in Nestin progenitor cells leads to increased ependymal cell differentiation, while S6K inactivation does not abolish rapamycin sensitivity.

(**A**) Representative images of primary cells from control and Tsc1 cKO mice at div 4 immunolabeled with FOP (centrioles), GT335, and pS6 antibodies. (**B**) Percentage of multicentriolar cells at div 4 and div 8; ****$P < 0.0001$; ns $P = 0.8182$. (**C**, **D**) Representative images of primary cells from Tsc1fl/fl mice infected with GFP (C) or Cre-GFP Adenovirus. Cells are immunolabeled with FOP and pS6 antibodies. Control is the same as in Fig. EV3C, as the experiments were carried out in parallel. (**E**) Percentage of multicentriolar cells at div 4 and div 8 in all conditions; *$P = 0.043$; ns $P = 0.6991$. (**F**) Representative pictures of wild-type or $S6K^{-/-}$ primary cells at 4 days in vitro, immunolabelled with pS6 as a marker of mTORC1 and FOP to detect multibasal bodies (MBB) cells in differentiated cells. The top panels are cells treated with vehicle (EtOH), and the bottom panels are cells treated with 20 nM Rapamycin. The arrows show examples of MBB cells. div differentiation in vitro, WT wild type. (**G**) Fold changes of differentiated ependymal cell number by counting cells with multibasal bodies. The bars represent the mean +/− SEM; each dot is one replicate, $n = 6$. P values were determined using a two-sided Student's t test. **$P < 0.01$ and ***$P < 0.001$. Data information: In (**B**, **E**), data are presented as mean ± SD. ****$P < 0.0001$, *$P \leq 0.05$, ns not significant (Student's t test). In (**G**), the bars represent the mean +/− SEM; each dot is one replicate, $n = 6$. P values were determined using a two-sided Student's t test. **$P < 0.01$ and ***$P < 0.001$. Ctrl Control.

© The Author(s)

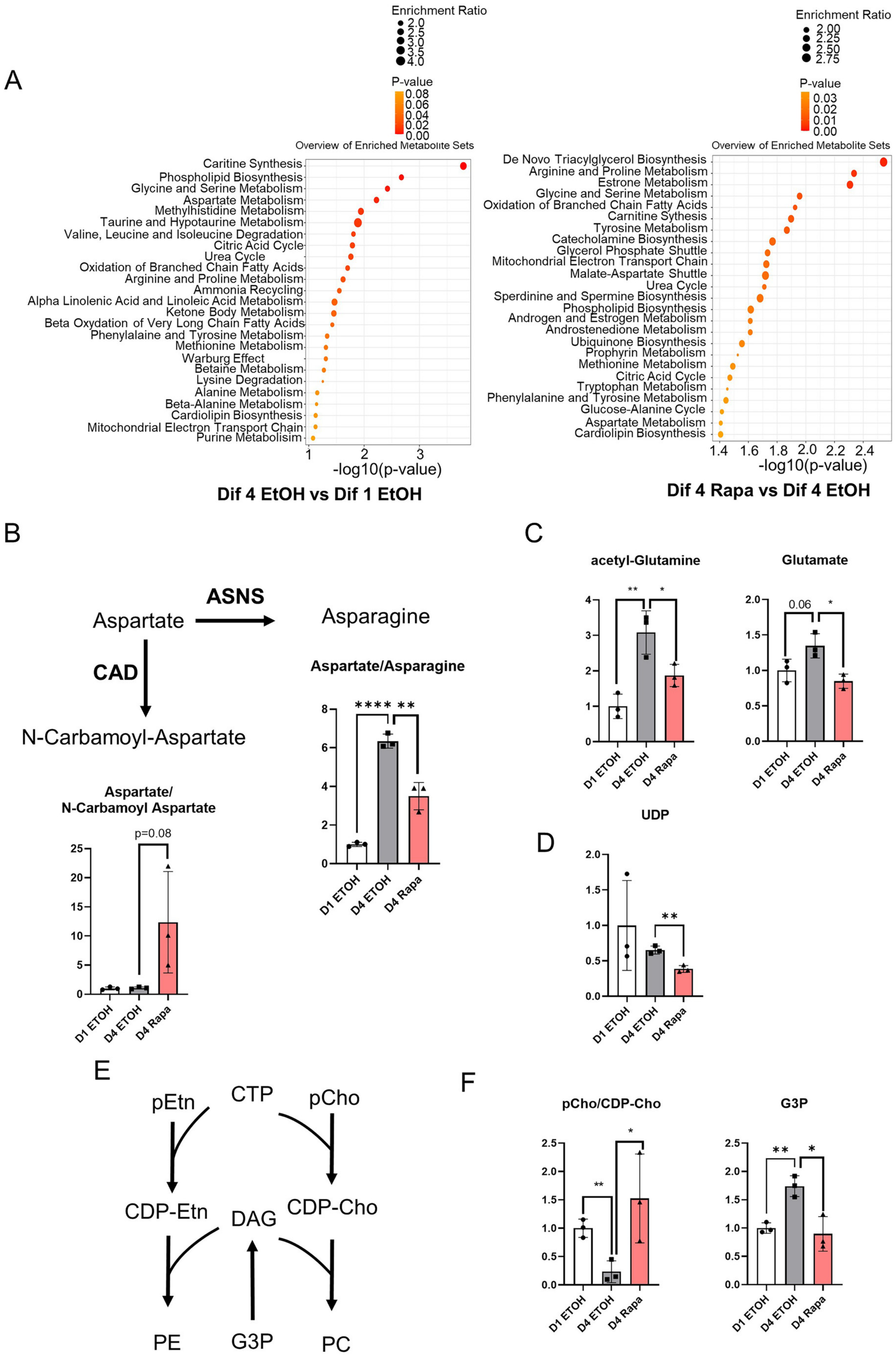
A
Dif 4 EtOH vs Dif 1 EtOH
Dif 4 Rapa vs Dif 4 EtOH
Overview of Enriched Metabolite Sets
Enrichment Ratio
P-value
-log10(p-value)
B
Aspartate
ASNS
Asparagine
CAD
N-Carbamoyl-Aspartate
Aspartate/Asparagine
Aspartate/ N-Carbamoyl Aspartate
p=0.08
C
acetyl-Glutamine
Glutamate
0.06
D
UDP
E
pEtn
CTP
pCho
CDP-Etn
DAG
CDP-Cho
PE
G3P
PC
F
pCho/CDP-Cho
G3P
D1 ETOH
D4 ETOH
D4 Rapa

 © The Author(s)

Figure EV5. Metabolic rewiring by mTORC1.

(**A**) KEGG-based pathway enrichment analyses of the metabolomes of progenitors and ependymal cells (left panel), or ependymal cells treated or not with Rapamycin. (**B**) Summary of the metabolic profiling of the aspartate metabolism in progenitors and ependymal cells treated or not with Rapamycin. (**C**) Levels of the indicated metabolites involved in glutamine metabolism in progenitors and ependymal cells treated or not with Rapamycin. (**D**) Levels of UDP in progenitors and ependymal cells treated or not with Rapamycin. (**E**) Scheme depicting the phospholipids biosynthesis pathways and highlighting the requirement of CTP and DAG for the second and third steps, respectively. (**F**) Ratios of the indicated metabolites and levels of G3P in progenitors and ependymal cells treated or not with Rapamycin. Data information: For (**B**, **C**, **D**, **F**), the bars represent the mean $+/-$ SEM, each dot is one replicate, $n = 3$. Data are presented as mean ± SD. ****$P < 0.0001$, ***$P < 0.001$, **$P < 0.01$, *$P \leq 0.05$ (Student's t test).
